# A Multi-cell, Multi-scale Model of Vertebrate Segmentation and Somite Formation

**DOI:** 10.1371/journal.pcbi.1002155

**Published:** 2011-10-06

**Authors:** Susan D. Hester, Julio M. Belmonte, J. Scott Gens, Sherry G. Clendenon, James A. Glazier

**Affiliations:** Biocomplexity Institute and Department of Physics, Indiana University Bloomington, Bloomington, Indiana, United States of America; University of Auckland, New Zealand

## Abstract

Somitogenesis, the formation of the body's primary segmental structure common to all vertebrate development, requires coordination between biological mechanisms at several scales. Explaining how these mechanisms interact across scales and how events are coordinated in space and time is necessary for a complete understanding of somitogenesis and its evolutionary flexibility. So far, mechanisms of somitogenesis have been studied independently. To test the consistency, integrability and combined explanatory power of current prevailing hypotheses, we built an integrated clock-and-wavefront model including submodels of the intracellular segmentation clock, intercellular segmentation-clock coupling via Delta/Notch signaling, an FGF8 determination front, delayed differentiation, clock-wavefront readout, and differential-cell-cell-adhesion-driven cell sorting. We identify inconsistencies between existing submodels and gaps in the current understanding of somitogenesis mechanisms, and propose novel submodels and extensions of existing submodels where necessary. For reasonable initial conditions, 2D simulations of our model robustly generate spatially and temporally regular somites, realistic dynamic morphologies and spontaneous emergence of anterior-traveling stripes of Lfng. We show that these traveling stripes are pseudo-waves rather than true propagating waves. Our model is flexible enough to generate interspecies-like variation in somite size in response to changes in the PSM growth rate and segmentation-clock period, and in the number and width of Lfng stripes in response to changes in the PSM growth rate, segmentation-clock period and PSM length.

## Author Summary

Recent decades have seen a revolution in experimental techniques that has shifted the focus of experimental biology from behaviors at the micron (cell) scale to those at the nanometer (molecular) scale. An ever-increasing number of studies detail subcellular behaviors, genetic pathways and protein interactions that relate to specific cell functions. This progress, while welcome, sometimes leads us to forget that these components do not exist or function in isolation. To understand their biological importance, in addition to exploring individual components in more detail, we must integrate them into comprehensive models of cells, tissues, organs and organisms. This integration has been incomplete for somitogenesis, an early developmental process that establishes the first signs of segmentation in all vertebrates, patterning the precursors of the vertebrae, ribs, and skeletal muscles of the back and limbs. In this paper, we make significant progress towards a comprehensive model of somitogenesis by combining specialized hypotheses for specific subcomponent mechanisms of somitogenesis into a unified multi-scale model that successfully reproduces many characteristic events seen in the embryo.

## Introduction

**Figure 1:**
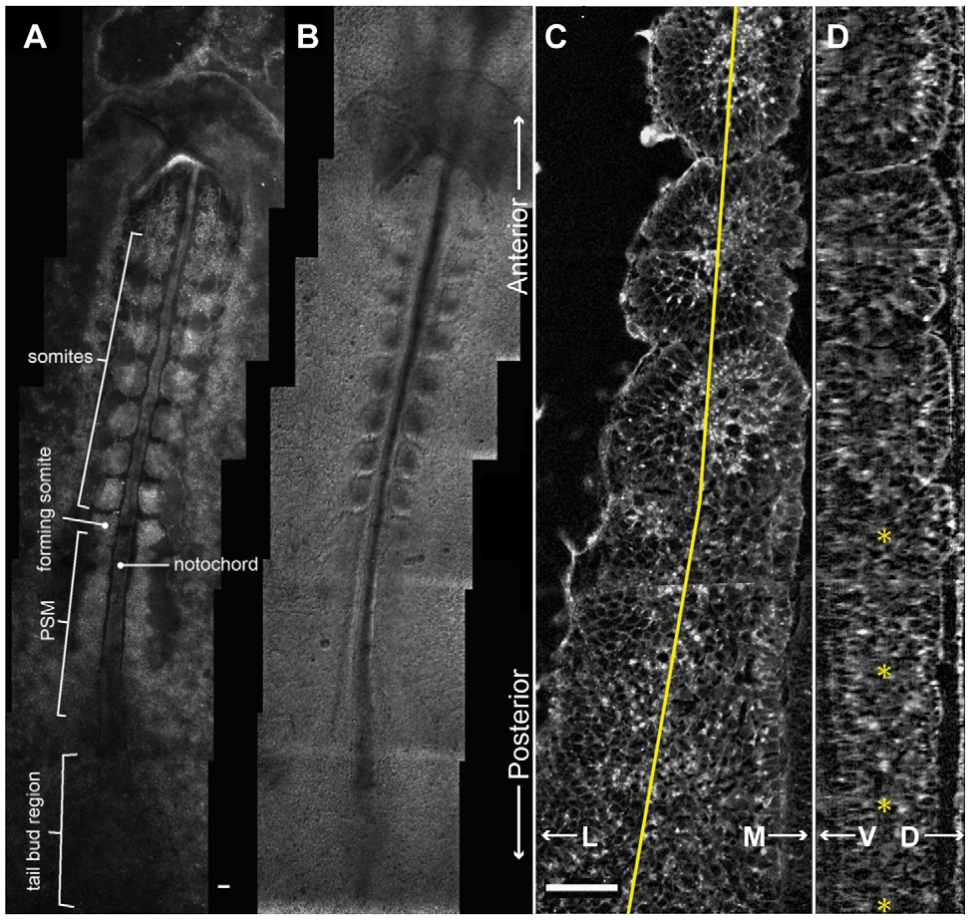
Chick PSM and somites. (**A**) Image of a live HH Stage 10 chick embryo stained with Lens culinaris agglutinin-FITC. (**B**) DIC image of the same embryo, (**C**) Coronal (ML-AP) and (**D**) sagital (DV-AP) slices of a single strip of the PSM and the most recent somites of a chick embryo at HH Stage 10, stained with Lens culinaris agglutinin-FITC. The PSM is relatively flat at the posterior end, and gradually becomes thicker towards the anterior end. We measured PSM DV thickness at the PSM midline (yellow line in (**C**)). Yellow *s in (**D**) indicate points where the thickness was measured. Measured thickness, from posterior (bottom) to anterior (top): 61 µm, 67 µm, 73 µm and 95 µm. The thickness through the center of the forming somite is 98 µm. In all panels, the anterior (head) is at top, posterior (tailbud) at bottom. Scale bars 40 µm.

*Somitogenesis*, the developmental process during which the presomitic mesoderm (*PSM*) lying on either side of the central notochord divides into a series of roughly spherical epithelial *somites* (**Figure 1**), establishes the earliest evident segmentation in vertebrate embryos [1]. Somite formation is regular in both time and space, with a pair of somites (one on either side of the notochord) forming and separating from the anterior of the PSM approximately every 30 minutes in zebrafish, every 90 minutes in chick, and every 120 minutes in mouse. An intricate cellular dance characterizes somite formation, with cells at the interface between a forming somite and the anterior PSM rearranging and pulling apart to form two distinct tissues separated by an *intersomitic gap* [2].

The striking spatio-temporal periodicity and dynamic morphology of somitogenesis depend on mechanisms operating across a range of scales, as well as interactions between scales: genetic and protein oscillations and regulatory networks at the *subcellular* scale [3]; *juxtacrine* (contact-dependent) and *paracrine* (secretion-dependent) cell-cell signaling [4], [5], and differential cell-cell adhesion at the *cellular* and *multicellular* scales [6], [7]; and PSM-spanning gradients [8], [9] and gene expression patterns [10] at the *tissue* scale.

Because somitogenesis involves interactions between many scales as well as coordination between events occurring in time and space, it is both uniquely interesting in its own right and a case study for the development of predictive and informative multi-scale models of development. Existing submodels addressing specific subcomponent mechanisms of somitogenesis have improved our understanding at individual scales and between scales, creating the impression that we are converging on a comprehensive understanding of somitogenesis. We have no assurance, however, that existing submodels are consistent and integrable with one another, or that, combined, they suffice to explain somitogenesis *in toto*. In this paper, we refine and extend current submodels, introduce additional submodels where needed to address interactions between them, and then combine them into an integrated model of somitogenesis. We identify inconsistencies preventing integration of existing submodels, missing or incomplete submodels, and reasonable hypothetical corrections and extensions to existing submodels. Finally, we investigate which experimental phenomena our resulting integrated models can produce.

Studying somitogenesis also provides insight into the evolvability of the vertebrate body plan. We show that our integrated model of somitogenesis is robust and flexible enough that it can describe somitogenesis in animals as different in size, shape and gestation time as chickens, garden snakes, mice and zebrafish. Modeling how mechanisms interact in time and space and across scales clarifies how a single segmentation strategy is flexible enough to generate such variation.

In this paper we focus on segmentation and somite formation in the chick embryo, and the features of somitogenesis that make it robust in the face of perturbations as well as flexible and evolvable enough to produce observed variations between species. We study somitogenesis between roughly HH Stage 8 (4 somite pairs) and HH Stage 16 (26–28 somites pairs). During this period, the PSM is relatively flat and of constant length. We do not model the initiation or termination of somitogenesis, or the formation of the specialized somites that form at the extreme anterior and posterior of the somitic tissues. Our model of chick somitogenesis extends to other vertebrates with minimal modifications.

### The clock-and-wavefront model

**Figure 2:**
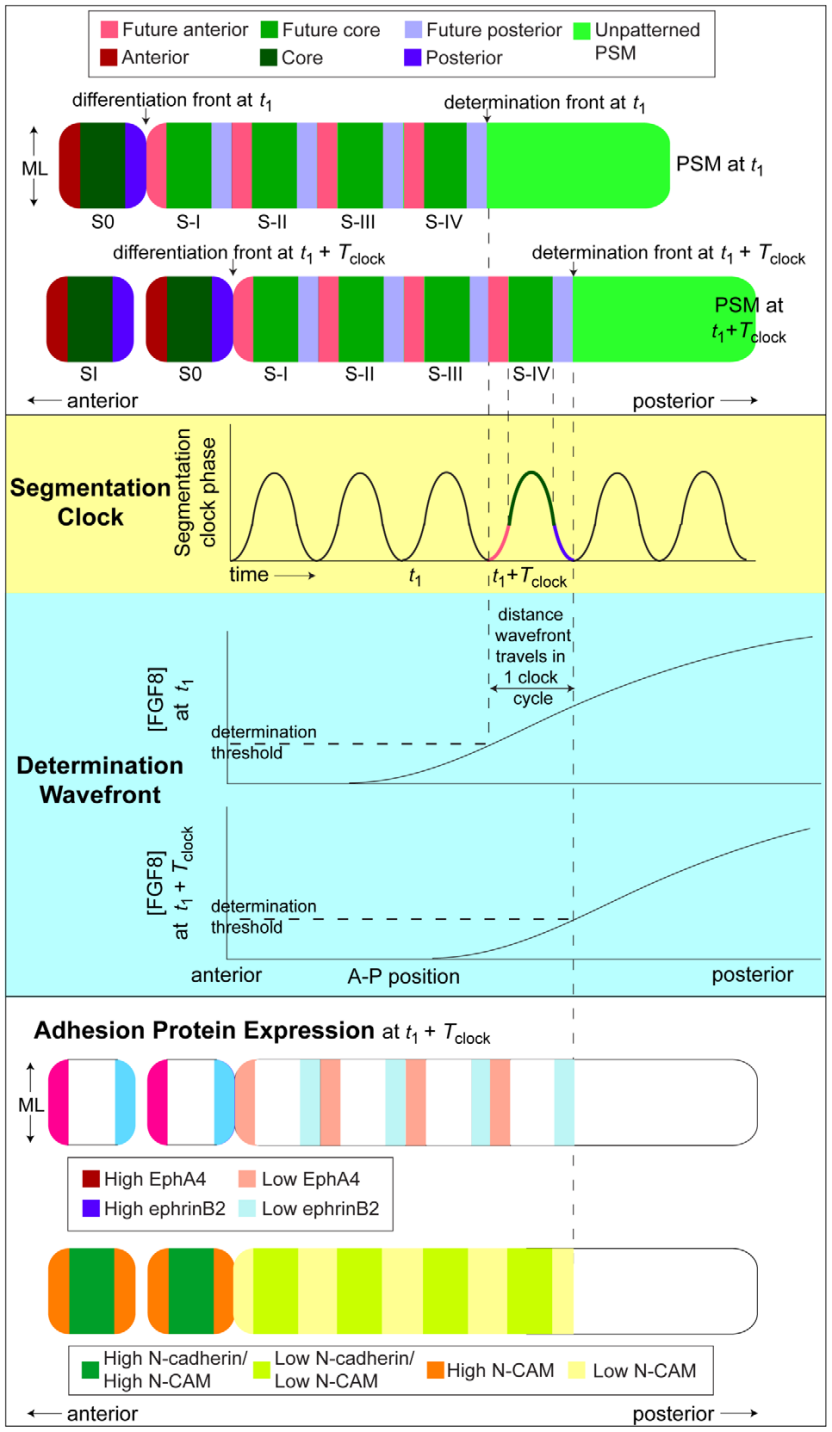
Schematic: A typical clock-and-wavefront model and its relationships to adhesion-protein expression. The AP position of a threshold concentration of temporally-decreasing FGF8 results in a posterior-propagating determination front, anterior to which a cell becomes competent to sense the state of its intracellular segmentation clock. At the determination front, a cell determines its fated somitic cell type (core, anterior or posterior) based on the state of its segmentation clock. Differentiation follows four segmentation clock periods (corresponding to four somite lengths) later. The PSM grows continuously in the posterior direction through addition of cells from the tailbud, maintaining its length. *T*_clock_ is the period of the segmentation clock. (*Below*) The clock-wavefront interaction results in the spatial pattern of adhesion protein expression that creates the differential adhesion between somitic cell types assumed in our computational implementation of the clock-and-wavefront model: EphA4 occurs in the anterior compartment of the forming somite and the anterior of the PSM; ephrinB2 occurs in the posterior compartment of the forming somite; N-CAM occurs throughout the anterior of the PSM and in the somites; and N-cadherin is strong in the core of forming and formed somites.

The *clock-and-wavefront* model, initially proposed by Cooke and Zeeman in 1976, describes a smoothly varying intracellular oscillator (the *segmentation clock*) that interacts with a posterior-propagating front of cell maturation in the PSM (the *wavefront*) to divide the PSM into periodic segments at regular spatio-temporal intervals [11]. Experiments have since borne out the model's central predictions, identifying candidates for both the clock and wavefront components in the PSM. This validation has boosted the model's popularity and led to a family of clock-and-wavefront models at all abstraction levels (ranging from purely qualitative biological models to mathematical descriptions to computational implementations). Recently, Baker *et al* have reviewed the various types of somitogenesis models including the clock-and-wavefront model [12], and have implemented sophisticated 1D mathematical clock-and-wavefront models [13], [14], [15]. Clock-and-wavefront models differ in detail but adhere to the core idea of Cooke and Zeeman that an intracellular segmentation clock and a posteriorly advancing wavefront establish and coordinate the temporal and spatial periodicity of somitogenesis. The integrated model that we will present builds on these concepts. **Figure 2** shows a schematic of the clock-and-wavefront model elements that we use in our integrated model.

The basic clock-and-wavefront model, while powerful, is not a complete explanation of somitogenesis. It lacks molecular explanations for numerous mechanisms observed in somitogenesis, including the origins and behaviors of the clock and wavefront; how the intracellular segmentation clocks interact between cells to maintain synchrony and phase-locking despite molecular noise, cell movement and cell division; how the clock and wavefront interact to induce cell determination and differentiation; how oscillating segmentation-clock molecules cause stable expression and localization of structural proteins like cell adhesion molecules; and, finally, how the distribution of structural molecules leads to the dynamics of segmentation and epithelialization. Various existing submodels address one or more of these aspects: *segmentation-clock submodels* address protein and mRNA oscillations within cells [16], [17]; *synchronization submodels* address crosstalk, synchronization and phase-locking between cells' segmentation clocks [5], [18]; *determination front* and *differentiation submodels* address the spatial progression of PSM maturation and somite formation [8], [13], [19], [20]; *clock-wavefront readout submodels* address the signaling and genetic regulatory events through which the segmentation clock and determination front interact to create a stable segmental pattern of gene expression in the PSM [21], [22]; and *cell adhesion submodels* address the cellular mechanics behind morphological changes during somite formation [7].

We drew on existing hypotheses of the intracellular segmentation-clock network from Goldbeter and Pourquié [16], Delta/Notch cell-cell segmentation-clock synchronization from Lewis [5], an Fgf8 threshold-positioned determination front from Dubrulle *et al.* [8], [20] and differential-adhesion-mediated morphogenesis from Glazier *et al.* [7]. As an example of the discriminatory power of building an integrated model, we found that we had to significantly alter and extend the Goldbeter-Pourquié intracellular segmentation-clock submodel [16] to make it compatible with Delta/Notch coupling and synchronization (based on [5]) between neighboring cells' segmentation clocks. Existing biological clock-and-wavefront readout submodels were insufficiently quantitative to allow creation of corresponding mathematical and computational models, so we developed our own readout submodel based on the available experimental data and previous speculative submodels.

Our integrated computational model, simulated in the Glazier-Graner-Hogeweg (*GGH*)-model-based CompuCell3D (*CC3D*) simulation environment [23], reproduces spatially and temporally regular formation of an unlimited number of somites for biologically reasonable initial conditions and parameter values, somite-to-somite variation in somite shape and border morphology consistent with experiments, and anteriorly traveling medio-lateral (*ML*) stripes of high Lfng and Axin2 protein/mRNA concentration in the PSM. Changing certain model parameters changes the somite size, frequency of formation and shape, giving us insight into which mechanisms may be responsible for observed interspecies variation. Somite size in our model depends on both the segmentation-clock period and the PSM growth rate (which determines the rate of determination front progression), while somite formation frequency depends on the segmentation-clock period. The number of Lfng stripes in our simulated PSM depends on the relationship between the segmentation-clock period, PSM growth rate and PSM length; and the relationship between the PSM growth rate and length depends on the mechanism that determines where and when cells differentiate.

In the following sections, we lay out key experimental observations on somitogenesis that have determined our biological submodels, describe previous submodels and our approach to their refinement and extension, discuss how we combined the refined submodels to build our integrated multi-scale clock-and-wavefront model, and, finally, present our simulation results.

### The presomitic mesoderm (*PSM*)

The vertebrate PSM is a dynamic, morphologically non-uniform tissue that cannot segment using static morphogen gradients. Cells constantly proliferate in the tailbud, exit the tailbud and enter the PSM. Periodically, a group of cells separates from the anterior of the PSM to form a new somite. Cells begin their residence in the PSM when they enter the posterior of the tissue from the tailbud and, as the anterior and posterior boundaries of the PSM move posteriorly, a cell's position within the PSM becomes progressively more anterior. Except at the initiation and termination of somitogenesis, cell addition and subtraction occur at similar rates, maintaining the PSM at a roughly constant length, so the PSM appears to travel posteriorly down the antero-posterior (*AP*) axis of the embryo, leaving a trail of somites in its wake.

In the posterior-most region of the PSM, cells are loosely associated and highly motile; directly anterior to this region, cells are less motile, adhere more strongly to one another and pack more closely; as their position in the PSM becomes more anterior, cells become even less motile and begin to form stable neighbor relationships [2], [6], [24], [25]. Some cell proliferation occurs within the PSM: in zebrafish, an estimated 10–15% of PSM cells divide during a single roughly thirty-minute segmentation-clock oscillation [26].

The posterior-most PSM is flat in the dorso-ventral (*DV*) dimension and widely spread medio-laterally. Moving in the anterior direction, the PSM gradually extends dorso-ventrally (**Figure 1** (**C–D**)); at the same time, it becomes increasingly restricted medially by the notochord and the neural tube (the *midline structures*), and laterally by an enveloping network of fibronectin-rich extracellular matrix (*ECM*) that surrounds the PSM. This ECM thickens and organizes into a tubular structure around the more mature PSM and somites [27], [28]. The PSM is further constrained dorsally and laterally by the epiblast and ventrally by the hypoblast.

In our model, we neglect cell division in the PSM, DV extension and asymmetries, and ML asymmetries, for reasons we discuss later.

### Morphogen gradients in the PSM

At least three signaling molecules form developmentally important gradients in the PSM. *Fibroblast growth factor 8* (*FGF8*) and the *wingless* homolog *Wnt3a* are both present at high concentrations in the tailbud and posterior PSM, and decrease anteriorly [8], [9]. Raldh2, which synthesizes retinoic acid (*RA*), a differentiation promoter, is expressed in newly-formed somites and generates a posteriorly-decreasing retinoid signaling gradient in the anterior PSM [29], [30].

Dubrulle and Pourquié [20] have characterized the mechanism that forms the FGF8 gradient: cells transcribe *fgf8* mRNA while residing in the tailbud and cease transcription once they enter the PSM, but continue FGF8 translation for as long as *fgf8* mRNA is present. Because cells move anteriorly relative to the PSM boundaries as they age, *fgf8* mRNA decay establishes an anteriorly-decreasing gradient that the FGF8 protein concentration mirrors [20]. Aulehla and colleagues [9] independently suggested that a related mechanism forms the Wnt3a signaling gradient in the PSM: *wnt3a* mRNA is expressed only in the tailbud and Wnt3a protein translation ceases in cells as they enter the PSM, so protein decay establishes a posterior-anterior signaling gradient. Finally, the RA and FGF8 signaling pathways are mutually antagonistic. Their coupling influences the shape of both the FGF8 and RA signaling gradients in the PSM [30]. For a recent review of signaling gradients in the PSM, see [31].

In the posterior and mid-PSM, strong FGF8 signaling maintains cells in an immature, undifferentiated state [8]. Permissively weak FGF8 signaling induces cell-type fate determination, which in chick occurs about four segmentation-clock periods (corresponding to four somite lengths) prior to differentiation into a somitic cell type [8]. FGF8 signaling also modulates motility in the PSM: strong FGF8 signaling leads to greater cell motility, and vice versa, leading to graded motility that is greatest in the posterior PSM [24].

In the present work, we model the formation of the FGF8 and Wnt3a gradients by *fgf8* mRNA and Wnt3a protein decay, and connect the signaling gradients to cells' intracellular segmentation-clock networks (discussed in the following sections). We omit detailed models of RA signaling for reasons that we discuss later. We also defer consideration of interference and reinforcement between morphogens, and signaling-induced changes in cell motility to future work.

### mRNA and protein oscillations in the PSM

Since oscillations were first observed in *c-hairy1* mRNA (downstream of Notch signaling) in chick PSM [10], entire cohorts of mRNA and protein oscillations have been observed in the PSM [3], [9], [32]. Her1 and Her7 oscillations downstream of Delta/Notch signaling are prominent in zebrafish [26], [33], [34], [35]. In mouse, gene expression downstream of FGF, Wnt and Delta/Notch signaling oscillates with the same period. Gene-expression oscillations downstream of FGF and Delta/Notch share a phase, and are half a period out of phase with gene-expression oscillations downstream of Wnt [3], [9].

These oscillations can occur cell-autonomously, persisting even in dissociated PSM cells [10]. Within the PSM, Delta/Notch signaling couples, synchronizes and maintains synchrony among neighboring PSM cells against noise from cell proliferation, stochastic gene expression and cell movement [26], [34], [35]. PSM cells initiate and synchronize their segmentation clocks during their sojourn in the tailbud and posterior-most PSM, before entering the more anterior region of the PSM modeled here [34].

Local synchronization of oscillations between neighboring PSM cells at the same AP position is crucial to segmentation [35]. However, PSM cells at different AP positions do not oscillate in phase. Instead, the phases of the oscillators display distinctive dynamic patterns across the PSM. In chick, lunatic fringe (*Lfng*) is initially expressed in the posterior PSM as a broad ML stripe that travels anteriorly, gradually slowing and narrowing before finally arresting in the anterior PSM at the location of the next presumptive somite. Other oscillating molecules in the segmentation-clock network display similar patterns [9], [10], [32], [36].

### Single-cell intracellular segmentation-clock network submodel

Goldbeter and Pourquié [16] developed a detailed model of the mouse/chick segmentation-clock network in a single cell, including independent FGF, Wnt and Delta/Notch oscillator loops. Each oscillator loop pathway includes negative feedback in which a downstream target of the signaling pathway inhibits the signaling that promotes its own transcription. In the FGF loop, Dusp6 inhibits the activation of ERK, an early player in the cascade leading to Dusp6 activation. Axin2 works in complex with Gsk3β to promote phosphorylation and subsequent degradation of β-catenin, a component of canonical Wnt signaling that upregulates transcription of Axin2. Finally, Lfng inhibits Notch signaling, while Notch signaling upregulates Lfng. Two connections couple the pathway loops: an unknown transcription factor in the FGF pathway (designated Xa and thought to be a member of the ETS family) upregulates Axin2 in the Wnt pathway, and uncomplexed Gskβ from the Wnt pathway inhibits Notch signaling [16]. If the coupling terms are omitted, simulations of the uncoupled FGF, Wnt and Notch loops oscillate autonomously, each with a different frequency. With the inter-loop coupling proposed by Goldbeter and Pourquié [16], the simulated oscillations phase-lock, with Lfng (in the Notch pathway) and Dusp6 (in the FGF pathway) oscillating in phase with one another and out of phase with Axin2 (in the Wnt pathway), as observed experimentally [3], [9].

### Delta/Notch segmentation-clock synchronization submodel

Motivated by the observation that synchronizing oscillations in the posterior PSM requires Delta/Notch signaling, Lewis [5] developed a biological model of the zebrafish segmentation clock composed of a single intracellular oscillatory network loop in the Delta/Notch signaling pathway that couples between adjacent cells via juxtacrine Delta/Notch signaling (**Figure S1**). Lewis developed mathematical and computational models of the network using a set of ODEs with terms representing the time delays for mRNA transcription and mRNA/protein transport in and out of the nucleus. Simulations of the model oscillate with the appropriate period and synchronize two initially unsynchronized cells [5]. Tiedemann *et al.* [37] developed a mathematical model of Lewis' network using coupled ODEs without delay terms by introducing compartmentalization of mRNA and protein in the cytoplasm and nucleus, and demonstrated that a static 2D array of Delta/Notch-coupled Lewis oscillators synchronized when initiated with random phases. We take the Lewis Delta/Notch mechanism for oscillator coupling and synchronization as the prototype for intercellular segmentation-clock coupling in our model.

### Extended three-loop segmentation-clock submodel with Delta/Notch coupling

We incorporated the Goldbeter-Pourquié description of the segmentation clock into our integrated model rather than choosing a simpler model (such as a phase oscillator, simple sine wave or single negative feedback loop) for two related reasons: (1) a primary aim in this work is to integrate current models of somitogenesis mechanisms at different scales; and (2) the Goldbeter-Pourquié model allowed us to explicitly model the connections between the segmentation clock and local FGF, Wnt and Delta signaling, and between the expression of oscillating clock molecules and the eventual differentiated states of cells (discussed later). To extend the Goldbeter- Pourquié model network to multiple cells, however, required us to include Delta/Notch coupling and synchronization between neighboring cells.

In a single cell, uninfluenced by outside factors, the loop-to-loop coupling mechanisms in the Goldbeter-Pourquié model maintain the appropriate phase relationships between the oscillators [16]. They are not, however, sufficient to explain observed behaviors of multiple cells. As currently understood, cell-cell synchronization occurs through the Notch pathway. Coupling between pathways in the Goldbeter-Pourquié model is directional: the FGF pathway feeds forward to the Wnt pathway, which feeds forward to the Notch pathway, with no feedback from the Notch pathway back to the FGF or Wnt pathways. Thus, the FGF and Wnt oscillators in Notch-coupled Goldbeter-Pourquié networks cannot entrain within or between cells. Experimentally-observed FGF- and Wnt-oscillator entrainment requires at least one additional coupling within the intracellular segmentation-clock network to allow the Notch oscillator to entrain the FGF and Wnt oscillators, or additional or modified juxtacrine signaling to entrain the FGF oscillators between cells.

We believe that experiments, while not conclusive, do suggest a feedback coupling from the Notch oscillator to the FGF oscillator. Hes7, a cycling gene downstream of Notch, regulates cyclic expression of Dusp4, a downstream FGF signaling inhibitor exhibiting the same set of behaviors as Dusp6 in the segmentation clock [38]. We introduced a generic Hes7-like inhibitory *Dusp modification factor (DMF)* into our submodel of the Notch signaling cascade, allowing the Notch loop to influence the FGF loop. Free Gsk3β generally inhibits Notch signaling [39], so adding DMF and Delta downstream of Notch also required us to model Gsk3β phosphorylation of the intracellular domain of cleaved Notch (*NICD*) in place of the direct inhibition of Lfng by Gsk3β previously modeled by Goldbeter and Pourquié.

**Figure 3:**
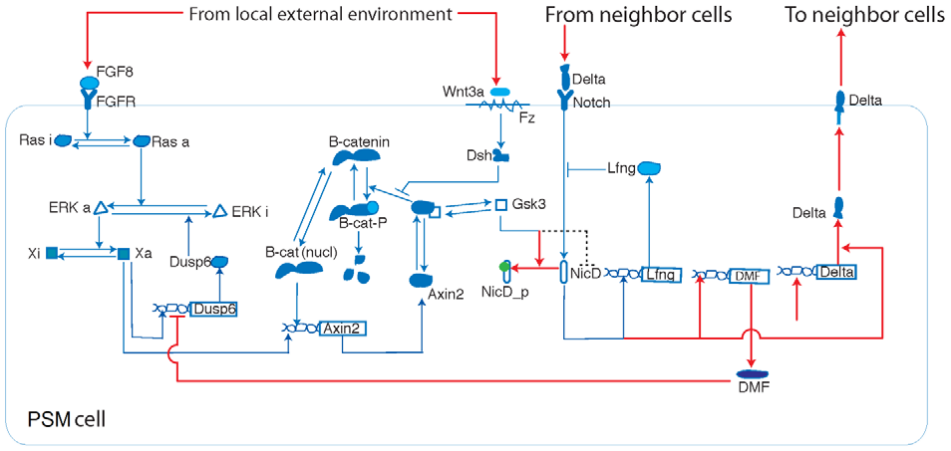
Schematic: Extended three-oscillator, externally-coupled biological sub-model for the segmentation-clock network. We adapted and extended the Goldbeter and Pourquié segmentation-clock biological model to include Delta signaling and to allow the experimentally observed phase locking between the FGF, Wnt and Notch loops in multiple coupled cells. Red lines show connections/processes in our biological model that are not in the Goldbeter-Pourquié biological model and dotted lines show connections in the Goldbeter-Pourquié biological model not used in our biological model. For more information, see **INTRODUCTION**: **Extended three-loop segmentation clock model with Delta/Notch coupling** and **METHODS** **: Segmentation clock** and **Coupling the segmentation clock to the morphogen fields**.

In our integrated model, the segmentation-clock network in each cell connects to FGF8 and Wnt3a signaling from the local environment and Delta signaling from the cell's immediate neighbors. **Figure 3** shows our segmentation-clock submodel and indicates our modifications of the original Goldbeter-Pourquié model.

### Patterns of adhesion proteins in the anterior PSM and somites

During and after the period in which cells at the anterior of PSM are reorganizing to form a new somite, they express a variety of cell-surface adhesion molecules that modify cell-cell interactions. These include homophilic N-CAM and N-cadherin, and heterorepulsive EphA4 and ephrinB2 (**Figure 2**). EphA4 and ephrinB2 are a complementary pair of surface receptors that are expressed in distinct ML bands in the forming somite and anterior PSM: EphA4 is expressed in the anterior compartments of forming somites and in the anterior tip of the PSM, while ephrinB2 is expressed in the posterior compartments of forming somites [22], [40], [41], [42] (see **Figure 2**). When juxtaposed, cells from these two populations induce bidirectional signals that change cell morphology, leading to an effective retraction and “repulsion” between the signaling cells. The precise mechanism behind Eph/ephrin-mediated repulsion is unknown, but is likely due to mutually-induced collapse of the cortical actin cytoskeleton [43].

N-CAM and N-cadherin are homophilic trans-membrane adhesion receptors that contribute to cell-cell adhesion. As a somite forms, N-cadherin expression increases and localizes predominantly to the apical surfaces of the epithelialized cells that form the outer layer of cells in the somite (contrary to the norm in most tissues, the apical surfaces face the somite core and the basal surfaces face the exterior of the somite) [6]. N-cadherin disruption leads to fragmentation of somites and separation of the anterior and posterior somite compartments [44]. N-CAM, which results in weaker, less specific adhesion than N-cadherin, is expressed relatively uniformly throughout the anterior PSM and the somites [6] (see **Figure 2**).

### Submodel of adhesion-driven cell sorting at somite borders

While several previous models describe mechanisms that lead to segmental patterns of gene expression in the PSM, very few explain how patterned gene expression produces morphological somite boundaries. Because the complex clock-wavefront interaction at cell determination is noisy, initial determination can result in significant misplacement of determined and differentiated cells. Forming repeatable somites of a specified size and shape that are separated by clean intersomitic gaps, as observed *in vivo*, requires a mechanism to refine the initial spatial distribution of cell types. One possible correction mechanism would be that cells remain labile after initial determination and can re-determine or re-differentiate in response to the predominant types of their neighbors. Another would be that misplaced cells undergo apoptosis in response to being surrounded by cells of a different type. Such mechanisms are potentially fast and work no matter how far a misplaced cell is from its appropriate location. However, neither of these mechanisms accounts for observed migrations of individual cells across the compartment boundaries within a forming somite or across a forming intersomitic boundary [2].

Glazier *et al.* [7] proposed that somite boundaries arise spontaneously through cell sorting due to intrinsic random cell motility and patterns of differential cell-cell adhesion resulting from adhesion-mediating molecules at cells' membranes. In their model, mutual repulsion between strongly EphA4- and ephrinB2-expressing cells leads to somite border and intersomitic gap formation. The anterior (EphA4) and posterior (ephrinB2) somite compartments adhere strongly to a core of highly adhesive cells with high concentrations of N-CAM and N-cadherin [7]. Simulations of Glazier *et al.*'s differential adhesion model reproduce many of the characteristics of somitogenesis *in vivo*, including somite compartment separation in the absence of N-cadherin and adhesion-induced-cell-migration correction of indistinct somite boundaries [7].

Our adhesion- and motility-based submodel of somite formation is a simplification of the Glazier *et al.* model [7]. While Glazier *et al.* used six somitic cell types, our differential cell-cell adhesion submodel uses three, representing cells with high EphA4 and high N-CAM, high ephrinB2 and high N-CAM, and high N-CAM and N-cadherin concentrations at their membranes. Furthermore, whereas Glazier *et al.* did not address the issues of cell-type determination prior to differentiation or the mechanisms that initially establish the spatial pattern of adhesion-protein expression, we combine the differential adhesion submodel with submodels of these mechanisms to address both of these issues.

### FGF8 determination front submodel

Based on the FGF8 concentration gradient in the PSM, the ability of FGF8 to maintain PSM cells in an immature state and the apparent determination of cell fates about four segmentation-clock periods prior to morphological differentiation, Dubrulle *et al.* [8] developed a biological model in which the advancing FGF8 gradient serves as the determination front in the clock-and-wavefront model. According to this model, below a threshold concentration of FGF8, cells become competent to respond to the states of their segmentation clocks, so cells posterior to the position of the FGF8 threshold are undetermined and cells anterior to the position of the FGF8 threshold have determined somitic fates (see **Figure 2**).

### Time-delayed and positional differentiation-front submodels

*In vivo*, cell differentiation involves continuous changes in cell properties, behaviors and interactions over a finite amount of time. We simplify differentiation in our biological model by describing it as occurring in two discreet, instantaneous steps. First, at determination, cells in our model begin to weakly exhibit the adhesion characteristics of their determined types, roughly approximating the early accumulation of adhesion-altering proteins at the membranes of biological cells and allowing some adhesion-mediated maintenance of future intrasomitic compartments prior to full differentiation. Some time later, at differentiation, cells undergo a second, more drastic change and assume the adhesion characteristics of their final differentiated states, which then drive somite formation, determine somite shape and maintain intrasomitic compartments.

The determination front model assumes that a second mechanism triggers differentiation some time after determination (approximately four segmentation-clock periods later in chick). In the course of our analysis, we considered two types of biological model for the delay between determination and differentiation.

In a *cell-autonomous delay model*, an intracellular “timer” counts down the time between cell determination and differentiation independent of the cell's external environment. Such a timer could represent, *e.g.*, the time a cell takes to express and manufacture adhesion proteins and localize them to the cell membrane, or the time the local FGF8 and/or Wnt3a concentrations take to fall below additional threshold concentrations that permit full differentiation. Short FGF8 [45] and Wnt3a [9], [46] diffusion lengths make local FGF8 and Wnt3a signaling nearly cell-autonomous; *fgf8* mRNA and/or Wnt3a protein decay effectively constitute the intracellular timer in such a scenario. We model both a generic intracellular timer that does not rely on any particular countdown mechanism and a second FGF8 differentiation concentration threshold. The choice of timer does not significantly affect our results (data not shown).

In a *positional differentiation front* model, the position of the differentiation front anterior to the determination front results from a separate signaling threshold mechanism that triggers determined cells to undergo full differentiation. The likeliest candidate for such a positional differentiation signal is RA originating in the somites and anterior tip of the PSM [45], [47], [48]. RA, which diffuses from the somites and anterior tip of the PSM into more posterior regions, has a concentration that depends on the distance from the anterior tip of the PSM and is independent of cells' history or their distance from the tailbud, except to the extent that distance from the tailbud and distance from the anterior end of the PSM are correlated. *In vivo*, mutually antagonistic opposing gradients of FGF8 and RA probably cooperate in positioning the differentiation front [45]. We do not explicitly model RA and RA-FGF8 interaction in the present work, choosing instead to model the simplest-case scenario involving morphogen-segmentation-clock interaction and threshold-positioned developmental fronts. Instead of explicitly modeling the RA concentration field when considering the positional differentiation front model, we make the simplifying assumption that once the PSM reaches its full length, a differentiation front begins at the anterior tip of the PSM and moves posteriorly at a constant speed equal to the rate of PSM growth, thus maintaining the PSM at a constant length. This assumption produces a differentiation front essentially indistinguishable from that for a simple two-gradient model that includes RA explicitly (data not shown). However, we would need to include RA if we wished to model the effects of RA perturbation experiments. We discuss additional possible impacts of this simplification in the **Methods** and **Discussion** sections.

### Clock-wavefront readout submodel

**Figure 4:**
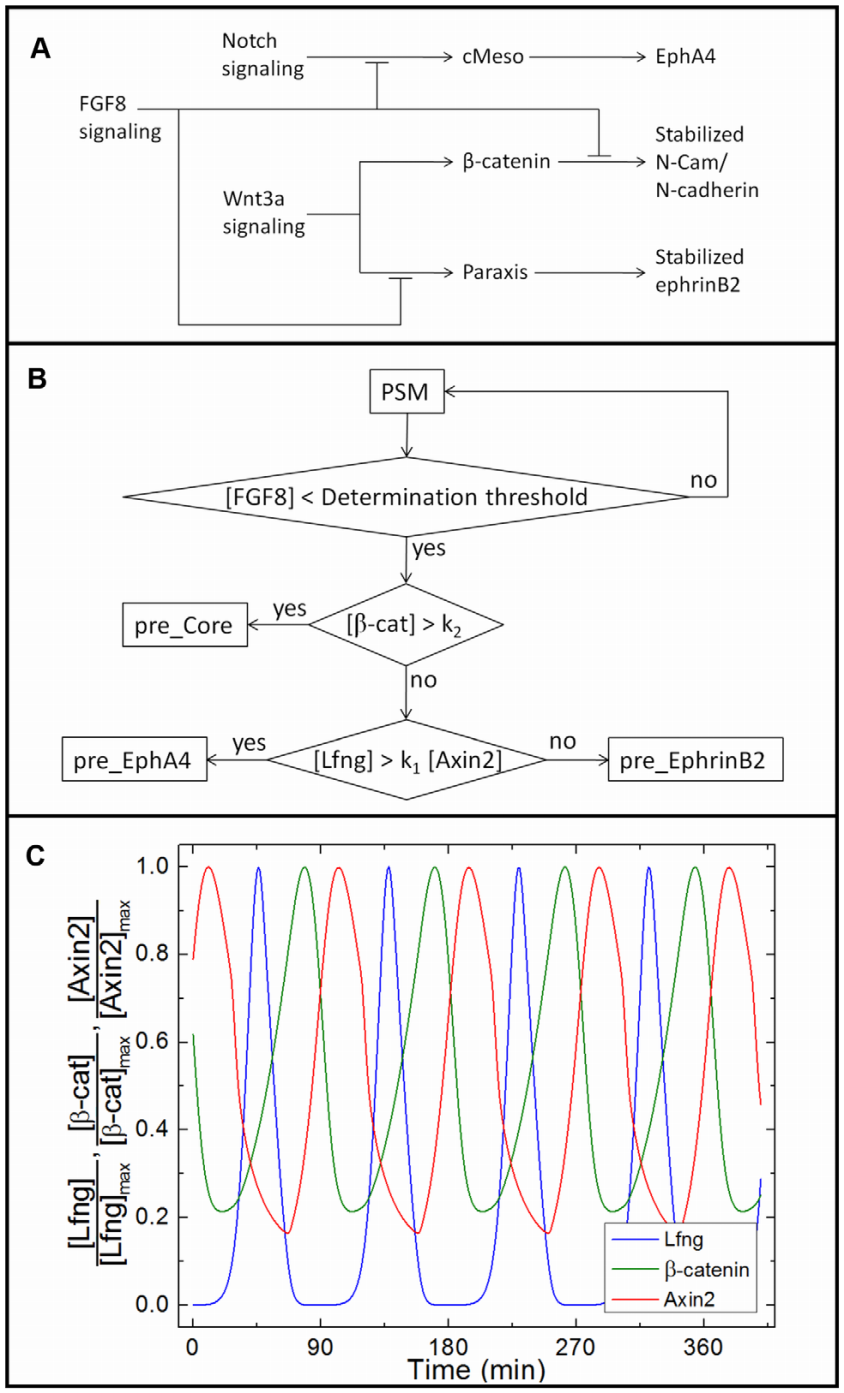
Clock-wavefront readout at the determination front. (**A**) Our biological submodel of the clock-wavefront readout network. Notch signaling regulates EphA4 through cMeso (Mesp2), cytoplasmic β-catenin in the Wnt3a pathway stabilizes N-CAM and N-cadherin at the plasma membrane, and functional ephrinB2 signaling requires Paraxis, downstream of Wnt3a signaling. When FGF8 signaling decreases below a threshold, it releases the inhibition of cMeso, Paraxis and N-Cam/N-cadherin, leading to expression of adhesion proteins on the cell membrane. (**B**) A schematic of the Boolean cell-type determination network submodel implemented in our computational model. The computational submodel is a simplified implementation of the biological submodel in (**A**). In our current computational model, *k_1_* = 21.28 and *k_2_* = 0.406 nM. (**C**) Time series of Lfng, β-catenin and Axin2 oscillations in a simulated **PSM cell** at the determination-front concentrations of FGF8 and Wnt3a ([FGF8] = 13.9 nM, [Wnt3a] = 0.55 nM). When the external FGF8 concentration falls below the determination threshold, the relative and absolute concentrations of Lfng, β-catenin and Axin2 determine the fate of the **cell** in our computational model according to the determination submodel in (**B**). For more information see **INTRODUCTION** **: Clock-wavefront read-out model** and **METHODS** **: Clock-wavefront model**.

An explanatory clock-and-wavefront model of somitogenesis requires a mechanism by which the segmentation clock and advancing wavefront interact to induce cell determination and subsequent differentiation. In our case, the biological clock-wavefront readout submodel must translate the concentrations of oscillatory segmentation-clock players at the time a cell first experiences below-threshold FGF8 signaling into stable patterns of eventual EphA4, ephrinB2, N-CAM and/or N-cadherin expression (see **Figure 2**). In our clock-wavefront readout submodel, Notch signaling regulates EphA4 through cMeso (Mesp2), cytoplasmic β-catenin in the Wnt3a pathway stabilizes N-CAM and N-cadherin at the plasma membrane, and functional ephrinB2 signaling requires Paraxis, downstream of Wnt3a signaling (**Figure 4** (**A**)).

Mesp2 (cMeso in chick) is a basic helix-loop-helix (*bHLH*) transcription factor whose localization in the anterior PSM is controlled by Notch signaling [49], [50]. A ML stripe of Mesp2 mRNA and protein appears one somite length posterior to the anterior tip of the PSM, marking the location where the next somite border will form [51]. Intersomitic border formation and normal intrasomitic compartmentalization both require Mesp2 [49], [50], [51], [52], [53], [54]. FGF8 signaling inhibits Mesp2 expression [24], which is consistent with an FGF8 threshold-as-wavefront model in which Mesp2 is an important mediator between the clock, wavefront and final determination of EphA4-expressing cells.

Cytoplasmically available β-catenin directly affects N-cadherin- and N-CAM-mediated cell-cell adhesion: β-catenin recruited from the cytoplasm by a membrane-associated complex stabilizes N-cadherin and N-CAM at the plasma membrane [55], and high levels of β-catenin saturate β-catenin binding to cadherin at the plasma membrane and increase cell-cell adhesion *in vivo* [56]. As one of the cycling components in the segmentation clock (see **Figure 3**), cytoplasmic β-catenin is thus an attractive potential link between the segmentation clock and N-cadherin/N-CAM expression.

AP compartmentalization and epithelialization of somites require the bHLH transcription factor Paraxis [57], [58], which is expressed in the anterior-most PSM and somites [8], [59]. Paraxis is a target of β-catenin-dependent Wnt signaling [60], and FGF8 signaling restricts its expression to the anterior of the PSM [8], making it a potential player in the clock-wavefront interaction. In Paraxis-null mice, ephrinB2 transcription in the somites is diffuse rather than restricted to the posterior of the somites [58]. The PSM of these mice partially segments, but intersomitic gaps do not form and the outer cells of the somites do not entirely epithelialize [57]. These characteristics suggest that the Paraxis-null somitic phenotype is a result of disrupted Eph-ephrin signaling, which is involved in epithelialization and gap formation [22], [40], [41], and which serves to segregate EphA4- and ephrinB2-expressing cell populations [7], [61].

### Modeled cell types

Certain terms we will use to describe our models differ slightly from their normal biological definitions. A ***cell type*** in our model denotes a collection of model **cells** that share a unique set of properties, interactions and dynamics. We will refer to model **cells**, **tissues**, **structures** and **cell-types** using **bold type**. **Cells** in our model occupy space (as opposed to being points), are deformable and motile (unless specified otherwise), and have variable adhesivity to other **cells**. The **cells** in our model are nonpolar and, with the exception of **Source cells**, have a constant volume and do not divide.

**Cell types** in our model reflect the simplification that differentiation happens in two steps. At determination, **cells** assume **cell types** with adhesion properties that are intermediate between undetermined **PSM** and differentiated **somitic cell types**. At differentiation, **cells** assume **cell types** with appropriate adhesion properties to form and maintain **somites**.

Our model has ten **cell types**: **Medium**, **Wall**, and **Source cells** do not correspond to actual biological cells, but represent the environment and structures surrounding the PSM; **PSM cells** represent undetermined cells in the modeled region of the PSM; **pre_EphA4**, **pre_ephrinB2**, and **pre_Core cells** represent cells with determined somitic cell types; and **EphA4**, **ephrinB2** and **Core cells** represent the somitic cell types. **Cells** of different **cell types** can differ in size, motility, adhesion to other **cells**, subcellular properties, contribution and response to signaling, and, ultimately, their roles in **PSM** and **somite** dynamics.

(0) **Medium** represents ECM and fluid in the tissue that is not explicitly modeled, and occupies any space that is not otherwise occupied by **cells**.
(1) **Wall cells** are arranged in immobile columns on either side of the **PSM**, representing the medial and lateral structures and extracellular material constraining the PSM to form a single anterior-posterior band. **Wall cells** disappear at the differentiation front to allow relaxation of **somite** boundaries and because they are no longer necessary to constrain **PSM** growth. **Wall cells** are the only **cells** in the simulation that have inflexible shapes and do not move.
(2) **Source cells** represent the addition of new cells to the modeled region of the PSM from the posterior-most PSM and tailbud. **Source cells** grow and divide at a constant rate to produce **PSM cells**; they are the only **cells** in the model that divide. Each **Source cell** contains a segmentation-clock network and high concentrations of *fgf8* mRNA, FGF8 and Wnt3a, which its progeny inherit. **Cells** in the posterior-most layer (those in contact with **Medium** at the posterior end) are, by default, **Source cells**: **Source cells** that fall out of contact with **Medium** at the posterior end of the **PSM** become **PSM cells**, and **PSM cells** that come into contact with **Medium** at the posterior end of the **PSM** become **Source** cells.

The remaining **cell types** represent biological cells in the PSM and somites.

(3) **PSM cells** represent cells in the modeled region of the PSM that are posterior to the determination front, and therefore do not have assigned somitic cell-type fates. They have higher motility and weaker cell-cell adhesion than other **cells** in the model. **PSM cells** each contain a segmentation-clock network and are under the influence of FGF8, Wnt3a and Delta signaling from the local field environment and surrounding **cells**. **PSM cells** do not transcribe *fgf8* mRNA or translate new Wnt3a protein, but do produce and secrete FGF8 from existing intracellular *fgf8* mRNA and signal with existing Wnt3a. **PSM cells** will become **pre_EphA4**, **pre_ephrinB2** or **pre_Core cells**, depending on the state of their intracellular segmentation clocks when they reach the determination front.

**pre_EphA4**, **pre_ephrinB2** and **pre_Core cells**, the *determined* ***cells***, represent cells in the PSM that are anterior to the determination front and posterior to the differentiation front (and so have been assigned fated somitic cells types but have not fully differentiated into their fated cell types). They are similar to **PSM cells**, with slightly lower motility and **cell-cell** adhesion strengths similar to those of **PSM cells**. Segmentation-clock networks in determined **cells** no longer oscillate, but do continue Delta signaling to neighboring **PSM cells**. In the differentiation signaling threshold submodel, determined **cells** differentiate in response to the differentiation threshold concentration of FGF8, but otherwise they do not respond to external signaling. Determined **cells** no longer secrete FGF8 or Wnt3a. Our results are not significantly influenced by discontinuing the segmentation-clock oscillations or FGF8, Wnt3a and Delta/Notch signaling in determined **cells** (data not shown).

(4) **pre_EphA4 cells** represent PSM cells that are fated to express high concentrations of membrane-bound EphA4 and to localize to the anterior compartment of the somite. They adhere slightly more strongly to **EphA4 cells** and other **pre_EphA4 cells** than to **cells** of other **cell types**. They will differentiate into **EphA4 cells** upon reaching the differentiation wavefront.
(5) **pre_ephrinB2 cells** represent PSM cells that are fated to express high concentrations of membrane-bound ephrinB2 and to localize to the posterior compartment of the somite. They adhere slightly more strongly to **ephrinB2 cells** and other **pre_ephrinB2 cells** than to **cells** of other **cell types**. They will differentiate into **ephrinB2 cells** upon reaching the differentiation wavefront.
(6) **pre_Core cells** represent PSM cells that are fated to express high concentrations of stabilized N-CAM and N-cadherin and relatively low concentrations of EphA4 or ephrinB2 at their membranes, and to localize to the center of the somite. They adhere slightly more strongly to other **pre_Core cells** than to **cells** of other **cell types**. They will differentiate into **Core cells** upon reaching the differentiation front.

**Cells** with the **somitic** **cell types** **EphA4**, **ephrinB2** and **Core** represent cells in the PSM and somites that are anterior to the differentiation front. They do not have segmentation-clock oscillations, nor do they secrete FGF8 or respond to FGF8, Wnt3a or Delta/Notch signaling. Their adhesive properties, which drive somite formation and maintenance, differ drastically from those of other **cell types**.

(7) **EphA4 cells** represent cells with high concentrations of EphA4 at their membranes, which make up the anterior compartments of the somites. They adhere weakly to **pre_EphA4 cells**, moderately to **Core cells** and other **EphA4 cells**, and strongly repulse **ephrinB2 cells**.
(8) **ephrinB2 cells** represent cells with high concentrations of ephrinB2 at their membranes, which make up the posterior compartments of the somites. They adhere weakly to **pre_ephrinB2 cells**, moderately to **Core cells** and other **ephrinB2 cells**, and strongly repulse **EphA4 cells**.
(9) **Core** cells represent cells with high concentrations of stabilized N-cadherin and N-CAM and relatively low concentrations of EphA4 or ephrinB2 at their membranes, which make up the centers of the somites. **Core cells** adhere moderately to **EphA4** and **ephrinB2 cells**, and strongly to other **Core cells**.

**Table 1** shows the relative degrees of adhesion and repulsion between **cells** of different **cell types** in our biological model.

**Table 1:** Strengths of adhesion and repulsion between cell types in our biological model.

Cell type	Medium	Wall	Source	PSM	pre_EphA4	pre_ephrinB2	pre_Core	EphA4	ephrinB2	Core
**Medium**	–	–	N	MR	MR	MR	MR	wr	wr	MR
**Wall**		–	MR	MR	MR	MR	MR	MR	MR	MR
**Source**			wa	wa	wa	wa	wa	wa	wa	wa
**PSM**				wa	wa	wa	wa	wa	wa	wa
**pre_EphA4**					wa	wa	wa	MA	wa	wa
**pre_ephrinB2**						MA	wa	wa	MA	wa
**pre_Core**							MA	wa	wa	wa
**EphA4**								MA	**SR**	MA
**ephrinB2**									MA	MA
**Core**										**SA**

N = Neutral; wa = Weak Adhesion; MA = Moderate Adhesion; **SA** = Strong Adhesion; wr = Weak Repulsion; MR = Moderate Repulsion; **SR** = Strong Repulsion; – = not applicable. For more information see **INTRODUCTION** **: Modeled cell types**.

### Two-dimensional model of the PSM

We model one column of the PSM as a two-dimensional AP-by-ML strip of motile, non-proliferating **cells**. The modeled **PSM** is ten average **cell** diameters wide so it forms **somites** containing approximately 100 **cells**, corresponding to the roughly 100 cells in a 2D mid-plane AP-by-ML cross-section of a somite in the chick embryo (see **Figure 1**).

We neglect the DV extension of the anterior PSM (and consequently the rounding of the somites in this direction), as well as the ML asymmetries in signaling and morphology that result from the presence of the midline structures. While DV extension and ML asymmetries are significant in biological somitogenesis, in particular affecting the epithelialization of forming somites, their effects are sufficiently weak that treating the PSM and somites as two-dimensional and medio-laterally symmetric is reasonable at the level of detail of our model.

**Figure 5:**
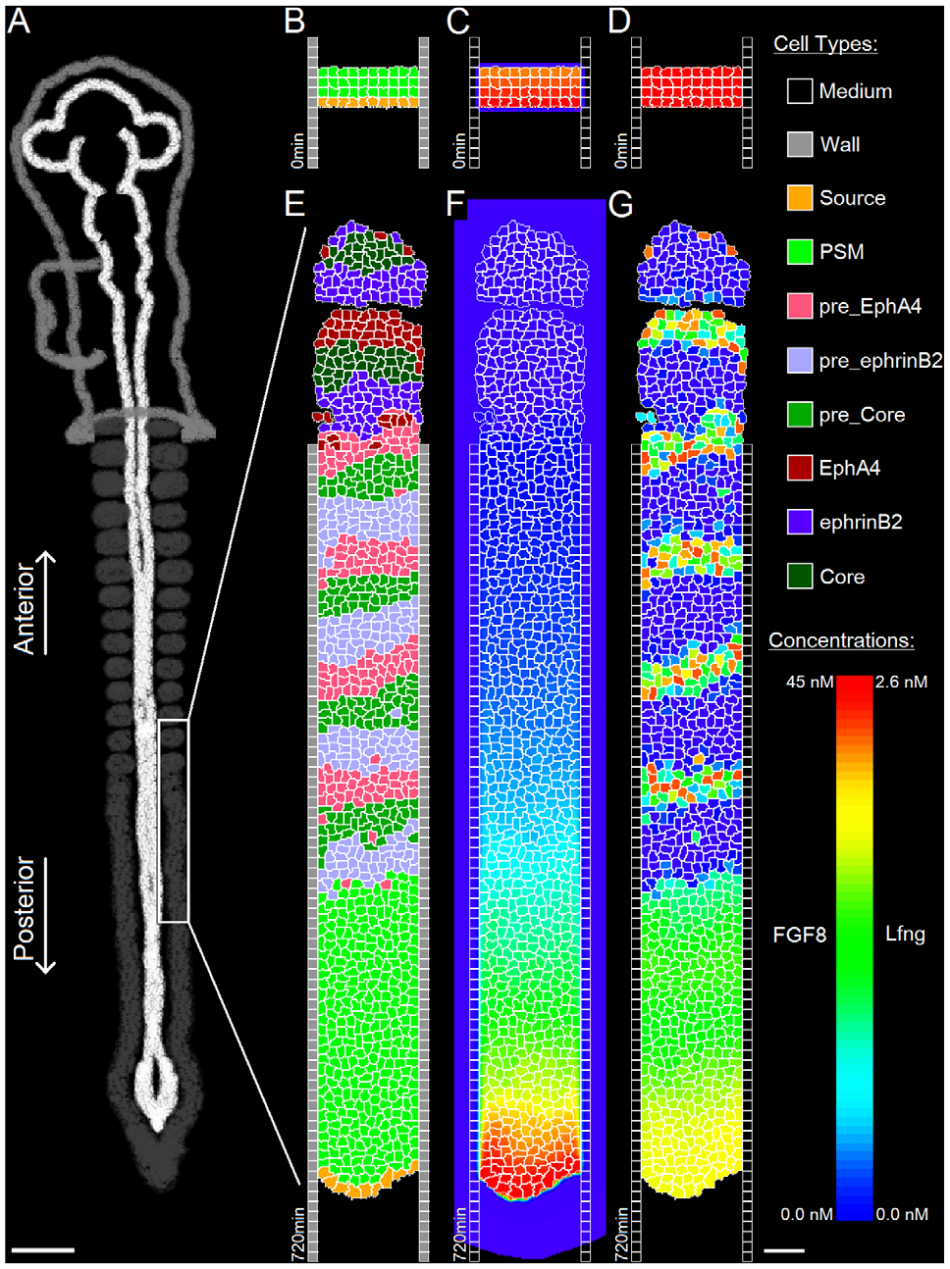
Initial conditions of our model. (**A**) Sketch of an experimental image of a chick embryo at HH stage 10 (dorsal view). Anterior end to the top and posterior end to the bottom. The modeled tissue extends approximately eight somite lengths posterior to the differentiation front. **Cells** in the modeled region have little intercellular ECM, so they contact each other directly. They adhere to one another and have limited motility. They do not transcribe *fgf8* or *wnt3a* mRNA, though they translate FGF8 and Wnt3a protein from the temporally decaying mRNA. Each **PSM** cell contains a segmentation-clock network submodel that couples the clock submodels in neighboring **cells** via contact-dependent Delta/Notch signaling. (**B**, **C**, **D**) Initial model conditions, visualizing (**B**) **cell** types, (**C**) [FGF8] and (**D**) [Lfng]. Not shown: initially, the constraining **walls** extend the full AP length of the simulation. (**E**, **F**, **G**) The modeled **PSM** after reaching its full length (at 720 min), visualizing (**E**) **cell** types, (**F**) [FGF8] and (**G**) [Lfng]. The patterns present in the full-length **PSM** arise spontaneously from the model's behavior. The first, ill-formed **somite** to the anterior of the full-length **PSM** results from the model's non-biological initial conditions. Parameters are the same as in the reference simulation (**Figure 7**). In (**B**–**G**) white color indicates **cell** boundaries. Scale bars: (**A**) 330 µm (**B**–**G**) 40 µm. For more information see **INTRODUCTION** **: Two-dimensional model of the PSM** and **METHODS** **: Initial conditions**.

We model the PSM beginning seven or eight somite lengths posterior to the most recent somite, where cells adhere moderately to each other and pack closely with little intercellular space [2], [6], [24], [25], and neighboring cells' segmentation-clock oscillations have already synchronized [34] (**Figure 5**). As we are primarily interested in somitogenesis rather than PSM formation in this paper, we do not model the tailbud or the elaborate cell migration paths by which cells leave the tailbud to enter the extreme posterior of the PSM [62], [63], [64]. We assume that PSM growth is due to addition of cells from the posterior, and omit division of **PSM cells** from the current model. To represent the steady addition of cells to the modeled PSM region, we define a layer of non-biological **Source cells** at the posterior end of the modeled **PSM** that grow and divide at a constant rate to produce new **PSM cells**.

We neglect AP changes in cell motility in the PSM, but do consider the effects of changing uniform cell motility. We assume cells to be isotropic. We plan to develop a more detailed three-dimensional model in future, including cell division in the PSM, greater consideration of the effects of the surrounding ECM and midline structures including ML and DV asymmetries, and graded cell motility and cell-cell adhesion in the PSM.

## Methods

### Experimental images

We fixed chick embryos at 36 hours of development using 4% paraformaldehyde in PBS pH 7.4 at 4°C for 24 h. We blocked and permeabilized the samples for at least one hour at room temperature in 0.5% Triton-X 100, 1% BSA, 2% serum, in PBS pH 7.4. We then labeled them using lens culinaris agglutinin-FITC (Vector Labs, Burlingame, CA) at a dilution of 1∶200 overnight at room temperature in the same buffer we used for blocking and permeablization. We then cleared the samples and acquired images with a Leica SP2 MP microscope using a 63× NA 1.2 water-immersion objective. We rendered image volumes using Voxx software [65].

### CompuCell3D (CC3D)

We implemented our computational models as simulations using CompuCell3D (*CC3D*) (available for download at http://compucell3d.org), an open-source software package designed to simulate multi-cell Glazier-Graner-Hogeweg (*GGH*) [66] models of cell behaviors in conjunction with intracellular genetic network or reaction-kinetic models and extracellular partial-differential-equation (*PDE*) models of tissue-level morphogen concentrations [67], [68], [69]. Instructions for downloading and running our simulation source code are given in **Text S2**.

### Glazier-Graner-Hogeweg (*GGH*) computational model of cell motility, adhesion and division

The GGH computational model represents space as a regular lattice of sites (or pixels). A GGH *generalized cell* may represent a biological cell, a subcellular compartment, a cluster of cells, or a piece of non-cellular material or surrounding medium. The **cells** from our biological model are simulated as GGH generalized cells. Each generalized cell is an extended domain of sites on a *cell lattice* that share a common index (referred to as the *cell index*, 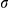).The cell-lattice configuration corresponds to an *effective energy* (*H*), defined so that simulated **cells** have the desired properties, behaviors and interactions, implemented via constraint terms in *H*. The effective energy in GGH simulations is not the actual energy of the biological cells and tissue being modeled but a simple way to specify the factors that govern **cell** properties, behaviors and dynamics in the simulated biological model. In our biological model, **cells** have volumes and surface areas, and interact via adhesion and repulsion, so that *H* is given by the following equation:
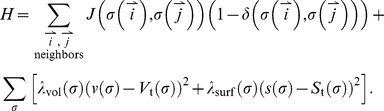
(1)


The first sum, over all pairs of neighboring lattice sites 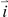 and 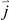, calculates the *boundary* or *contact energy* between neighboring **cells**. 
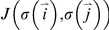
 is the boundary energy per unit contact area for **cells** 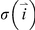 and 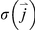 occupying sites 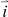 and 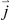, respectively, and the delta function restricts the contact energy contribution to **cell**-**cell** interfaces. We specify 
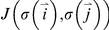
 as a matrix according to the **cell types** of 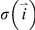 and 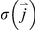. Higher (more positive) contact energies between **cells** result in greater repulsion between the **cells** and lower (more negative) contact energies between **cells** result in greater adhesion between the **cells**.

The second sum in (Eq. 1), over all **cells**, calculates the effective energies due to the volume and surface-area constraints. Deviations of the volume or surface area of **cell** 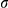 from its target values (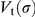 or 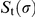, respectively), increase the effective energy, penalizing these deviations. On average, a **cell** will occupy a number of pixels in the cell lattice slightly smaller than its target volume due to surface tensions from the contact energies (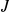). The parameters 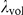 and 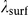 behave like Young's moduli, with higher values reducing fluctuations of a **cell**'s volume or surface area about its target values.

**Cell** dynamics in the GGH model provide a much simplified representation of cytoskeletally-driven cell motility using a stochastic modified Metropolis algorithm consisting of a series of index-copy attempts. Before each attempt, the algorithm randomly selects a target site, 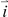, and a neighboring source site 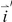. If different **cells** occupy those sites the algorithm sets 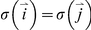 with probability 
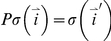
, given by the Boltzmann acceptance function:
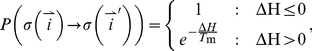
(2)where 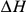 is the change in the effective energy if the copy occurs and 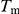 is a global parameter describing cell membrane fluctuations that we will discuss momentarily. A Monte Carlo Step (*MCS*) is defined as *N* index-copy attempts, where *N* is the number of sites in the cell lattice, and sets the natural unit of time in the computational model.

The average value of the ratio 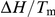 for a given **cell** determines the amplitude of fluctuations in the **cell** boundaries that are a simplified representation of the cytoskeletal fluctuations that drive cell motility. High 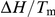 results in rigid, barely- or non-motile **cells** and little **cell** rearrangement. For low 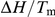, large fluctuations allow a high degree of **cell** motility and rearrangement. For extremely low 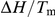, **cells** may fragment in the absence of a constraint sufficient to maintain the integrity of the borders between them. Because 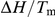 is a ratio, we can achieve appropriate **cell** motilities by varying either 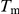 or 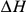. Variations in 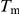 allow us to explore the impact of global changes in cytoskeletal fluctuations (*e.g.*, to mimic an experiment using cytochalasin). By changing 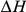, we can influence the relative motility of the **cell types** or of individual **cells** by varying, for example, the parameter 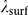, the target surface areas (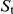) or the contact energies (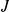) between **cells**.

The Metropolis algorithm evolves the cell-lattice configuration to simultaneously satisfy the constraints, to the extent to which they are compatible, with perfect damping (*i.e.*, average velocities are proportional to applied forces).

 A potential index copy that increases the effective energy, *e.g.*, by increasing deviations from target values for **cell** volume or surface area or juxtaposing mutually repulsive **cells**, is improbable. Thus, the pattern evolves in a manner consistent with the biologically-relevant “guidelines” incorporated in the effective energy: **cells** maintain surface areas and volumes close to their target values, mutually adhesive **cells** (with low **cell-cell** contact energy) stick together, mutually repulsive **cells** separate, *etc*… Thus, the average time-evolution of the cell lattice corresponds to that achievable deterministically using finite-element or center-model methodologies with perfect damping.

For a further introduction to GGH modeling, see [23].

### GGH cell types

A GGH **cell type** distinguishes **cells** that share a unique set of behavioral mechanisms, parameters and submodels. Same-**type** **cells** may have additional parameters and variables which differ between **cells** of that **type**. The GGH **cell types** in our model correspond to the **cell types** in our biological model (see **INTRODUCTION**). We will discuss their properties and parameters later.

### Chemical fields and the GGH computational model

We represent chemical concentrations as pixelized chemical fields using a *field lattice* with the same pixel size as the cell lattice. In our mathematical models, we use PDEs to represent **cell** interactions with the chemical fields (secretion and absorption), diffusion and decay. Our simulations use the diffusion solvers packaged with CC3D, sometimes in combination with custom-coded descriptions of **cell**-specific secretion or absorption.

In our two-dimensional computational models, we allow chemicals to diffuse freely through **cells** (including **Wall cells** and **Medium**) and neglect advection due to **cell** movement. This approximation is common in tissue simulations (*e.g.*, those in [70], [71]), and is a less severe approximation than our reduction from 3D to 2D. Moreover, real PSM cells are mesenchymal and have some intercellular space and ECM through which signaling molecules diffuse. We can think of our modeled **cells** as representing both the biological cells and the immediately surrounding external material/space through which diffusion occurs. Because the model of the segmentation clock is insensitive to FGF8 concentration, we would not expect a significant change in our results with excluded volume diffusion, even with a different FGF8 diffusion length.

### Python scripting and custom simulation modules in CC3D

We implement our computational models as simulations using Python scripts containing custom modules written as classes (see Supporting Information **Text S2**). Typically, each simulation class implements a single submodel or process, *e.g.*, a subcellular network, **cell** growth and division, or **cell** secretion; or a set of related submodels or processes, *e.g.*, paracrine or juxtacrine signaling and subcellular signaling pathways. A simulation class can assign properties or attributes to **cells** (***cell*** *attributes*) that are accessible from other modules, *e.g.*, a Boolean variable indicating whether a **cell** behavior is active, or an entire implementation of a subcellular genetic or reaction-kinetic network.

A separate simulation configuration script (in either Python or CC3DML) registers the modules, defines **cell types** and default **cell-type**-dependent contact energies, designates chemical fields, sets GGH-related parameters and boundary conditions, and specifies initial conditions (see **Text S2**).

For further information on CC3D simulation software, see [23], or refer to the CompuCell3D website (http://compucell3d.org).

### Time and length scales

The natural length and time scales in GGH computational models and simulations are pixels and MCS, respectively. We relate these to biologically-relevant units in such a way that events in time and space in our model correspond to those *in vivo*. Specifically, we use **cell** diffusion, morphogen diffusion, **cell** diameter, **somite** size and the segmentation-clock period to convert between “model time” and “biological time” in the following way: 1 pixel in the simulations corresponds to 1.43 µm (1 µm = 0.7 pixels) and 1 MCS corresponds to 0.015 min (6000 MCS = 90 min, the duration of one somite cycle).

### Cell types in our computational model

The **cell types** in our computational model correspond to those in our biological model (see **INTRODUCTION**). To develop our computational model, we assigned to each **cell type** GGH parameters for target volume (or surface area in two dimensions), target surface area (or boundary length in two dimensions), and volume and surface-area constraint parameters 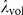 and 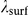. **Table 2** gives these parameters, along with other characteristics of our computational-model **cell types**.

**Table 2:** Characteristics and behaviors of model **cell** types.

	Parameters			Behaviors					
Cell type	Diameter (µm)	Surface (µm)*	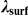	Diffusion constant (µm^2^/min)	Grow/Divide	SecreteFGF8	Clock network	Delta signal	Delta respond
**Medium**	–	–	–	–	–	–	–	–	–
**Wall**	10	40	–	–	N	N	N	N	N
**Source**	10–20	40–80	15	–	Y	Y	Y	Y	Y
**PSM**	10	40	15	1.08	N	Y	Y	Y	Y
**pre_EphA4**	10	40	15	1.01	N	Y	N	Y	N
**pre_ephrinB2**	10	40	15	1.01	N	Y	N	Y	N
**pre_Core**	10	40	15	0.98	N	Y	N	Y	N
**EphA4**	10	40	15	1.02	N	N	N	N	N
**ephrinB2**	10	40	15	1.02	N	N	N	N	N
**Core**	10	40	15	0.95	N	N	N	N	N

N = behavior lacking; Y = behavior present; – = not applicable.

*Because our model is two-dimensional, the Surface is actually a cell boundary length, with units of length, not area. For more information see **METHODS** **: Cell types in our computational model**.

We also specified the *contact energy matrix*, in which we designated the GGH contact energies that represent the adhesive and repulsive interactions between **cell types** (**Table 3**). We estimated GGH contact energies to approximate the relative adhesion and repulsion strengths between biological cells with different concentrations of adhesion molecules at their membranes. While we did not perform an exhaustive search over all possible contact energies, a modest exploration of contact energies did not significantly affect the resulting **cell** behaviors, provided that the contact energies maintained the hierarchy we show in **Table 3**. Careful study of the effects of contact energies in our previous study of the morphological dynamics of somitogenesis [7] increases our confidence that the exact contact energies are not crucial.

**Table 3:** GGH contact energies between **cell** types for reference simulation.

Cell type	Medium	PSM	Source	pre_EphA4	pre_ephrinB2	pre_Core	EphA4	ephrinB2	Core	Wall
**Medium**	0	15	0	15	15	15	5	5	15	0
**PSM**		−20	−20	−20	−20	−20	−20	−20	−20	30
**Source**			−20	−20	−20	−20	−20	−20	−20	30
**pre_EphA4**				−25	−20	−20	−25	−20	−20	30
**pre_ephrinB2**					−25	−20	−20	−25	−20	30
**pre_Core**						−35	−20	−20	−20	30
**EphA4**							−25	80	−25	30
**ephrinB2**								−25	−25	30
**Core**									−40	30
**Wall**										–

Positive contact energies represent repulsive interactions; negative contact energies represent adhesive interactions. Larger contact energy magnitudes indicate stronger interactions. For more information see **METHODS** **: Cell types in our computational model** and **Differentiation**.

### Cell motility

In contrast to many other models [18], [37], [72], [73], our **cells** have explicit shapes and degrees of movement. We take advantage of the latter to study the effect of cell motility in somitogenesis. In our model we vary the motility of **cells** by varying the degree of **cell** membrane fluctuation, which is regulated by the parameter 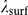 (larger 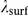 leads to higher average 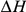, which reduces **cell** motility). This choice has a biological motivation; motility in biological cells is associated with the degree of membrane ruffling and higher 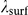 decreases **cell**-boundary ruffling amplitude [74]. The resulting effect on **cells'** diffusion rates is shown in **Table 8**.

### Initial Conditions

We model somitogenesis beginning after the formation of the first four somites, when the PSM has already grown to the length it will maintain through the subsequent formation of 22–24 additional somites. To avoid biasing the evolution of the model with a pre-imposed pattern, however, we initialize the model with only four layers of **cells** between two columns of confining **Wall cells** and allow the **PSM** to grow to its full length from those initial conditions (**Figure 5**).

We initially define two columns of **Wall cells** that run the length of the simulation and represent the medial and lateral structures confining the growth of the PSM, three layers of **PSM cells** spanning the ML space between the **Wall** columns and, posterior to them, a single layer of **Source cells**. We initialize the **Source cells** with concentrations of 5 nM *fgf8* mRNA and 45 nM FGF8, and the first three layers of **PSM cells** with 4.8 nM *fgf8* mRNA and 43.2 nM FGF8, 4.6 nM *fgf8* mRNA and 41.4 nM FGF8, and 4.4 nM *fgf8* mRNA and 39.6 nM FGF, moving from the posterior layer to the anterior layer, to emulate the progressive decay of *fgf8* mRNA and FGF8 in these **cells** after leaving the tailbud. We initialize the segmentation-clock networks within the **Source** and **PSM cells** with identical phases; we do not impose synchrony on the **Source cells**, but allow them to interact with their neighbors. Our initial conditions result in the formation of a single ill-formed **somite** once the model **PSM** reaches its full length, after which normal **somites** form (see **Figure 5**).

### Morphogen gradients

**Source** and **PSM cells** in our biological model secrete FGF8 and Wnt3a proteins that diffuse and decay in space. In our computational model, each **Source** and **PSM cell** has an internal concentration of *fgf8* mRNA (attached as a **cell** attribute in CC3D) that determines the **cell**'s FGF8 secretion rate. **Source cells** have a constant concentration of *fgf8* mRNA, *mfgf*_0_, that **PSM cells** inherit from their parent **Source cells**. In **PSM cells**, *fgf8* mRNA decays exponentially in time with a decay constant 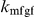:
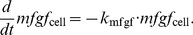
(3)


**Figure S2** (**A**) shows the evolving cellular *fgf8* mRNA concentration averaged in the ML direction versus AP position.

*In vivo*, cells translate *fgf8* mRNA into FGF8 protein before secreting FGF8 into the intercellular space, where it binds to receptors on that and other cells' membranes to induce the intracellular FGF signaling cascade. We simplify this process in our computational model, first by setting **PSM** and **Source cells**' FGF8 secretion rates directly proportional to their intracellular concentrations of *fgf8* mRNA:
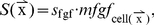
(4)where 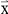 is a field lattice site corresponding to a cell lattice site occupied by the **cell**, and each **PSM** and **Source cell** secretes FGF8 from every pixel it occupies. Second, **cells** in our model do not impede diffusion (**cells** and FGF8 co-occupy space), nor is FGF8 consumed during signaling, so that the local FGF8 concentration obeys the two-dimensional diffusion equation with secretion from (Eq. 4):
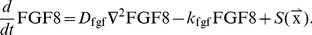
(5)


Finally, we simplify FGF8 signaling in our mathematical and computational models by not modeling the interaction between extracellular FGF8 and cellular transmembrane FGF receptor proteins. Biologically, cells in the PSM generally express FGFR1 [4]; in constructing our computational model, we assume that intracellular FGF signaling is proportional to the local FGF8 concentration and is not affected by the concentration of FGFR1 on a cell's surface. These assumptions lead to FGF8 signaling (Eqs. 3–5) similar to that modeled in one dimension by Baker *et al.* [13]. Because the intracellular segmentation clock is the only cellular property influenced by FGF8 in the posterior **PSM**, where FGF8 concentration is very high, and because the model clock's response to FGF8 saturates at a very low FGF8 concentration (discussed in the next subsection), we are not concerned with possible effects of FGF receptor saturation. In our computational model, the FGF8 signaling experienced by a **cell** is the FGF8 concentration at the **cell**'s geometric center.

At the simulation level, a custom simulation class handles *fgf8* mRNA decay and **cell**-by-**cell** secretion, and the basic CC3D diffusion solver handles FGF8 diffusion and decay outside of **cells**. FGF8 diffuses freely (**cells**, including **Wall** and **Medium**, do not impede diffusion) with Neumann boundary conditions. Because FGF8 has a diffusion length shorter than a **cell** diameter (for the diffusion and decay constants estimated in [45]) and the FGF8 concentration field has decayed to zero near the simulation boundaries, the choice of global simulation boundary conditions does not significantly affect simulation results. **Figure S2** (**B**) shows the FGF8 field for a typical unperturbed simulation.

We used FGF8 diffusion and decay constants estimated by Goldbeter *et al.* [45]. We chose the initial intracellular *fgf8* mRNA concentration and *fgf8* mRNA decay rate, which determine the amplitude and shape of the AP FGF8 gradient, and the determination FGF8 concentration to position the determination front roughly eight somite lengths anterior to the **Source cells** (**Table 4**). Our model produces roughly exponential morphogen gradients to reflect observations *in vivo* [20]. However, using linear gradients with appropriate clock-wavefront interaction parameters rather than exponential gradients does not significantly alter our results (data not shown).

**Table 4:** Parameters for FGF8 and Wnt3a fields in reference simulation.

Parameter	Value
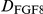	0.6 µm^2^/min
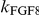	0.2 min^−1^
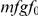	5.0 nM
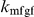	0.005 min^−1^
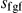	1.83 min^−1^
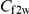	0.32

The biological values of the first three parameters were estimated from Goldbeter *et al.*, 2007, while the others were estimated by the simulation. For more information see **METHODS** **: Morphogen gradients**.

**Cells** carry their *fgf8* mRNA concentrations with them and secrete FGF8 at all sites within the **cell**, so the FGF8 source term [4] in the apparently deterministic diffusion equation (Eq. 5) is stochastic, reflecting the stochasticity of **cell** motion and addition of **cells** to the **PSM** by the daughter **cells** of **Source cells**. Thus, the *fgf8* mRNA and FGF8 fields are noisy. Noise in the *fgf8* mRNA and FGF8 fields increases with increasing **cell** motility, as expected, though diffusive smoothing decreases the noise in the FGF8 field compared to that of the *fgf8* mRNA field for all **cell** motilities (**Table 5**). We reiterate that the AP gradient arises from **cell**-autonomous intracellular *fgf8* mRNA decay rather than diffusion of FGF8, which has a diffusion length much smaller than the length of the tissue, a phenomenon that has developmental and evolutionary implications (discussed later).

**Table 5:** Noise in FGF8 and *fgf8* mRNA fields for different cell motilities.

	Low cell motility	Reference cell motility	High cell motility
**Noise in [** ***fgf8*** ** mRNA]**	3.49%	4.78%	8.50%
**Noise in [FGF8]**	2.11%	3.96%	6.38%

Noise calculated as the standard percent deviation of the simulation data from the best-fit exponential function averaged over all times. Low motility: 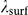 = 25, D = 0.86 µm^2^/min. Reference motility: 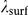 = 15, D = 1.08 µm^2^/min. High motility: 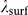 = 5, D = 1.76 µm^2^/min. For more information see **METHODS** **: Morphogen gradients**.

To reduce computation time and because no experimental evidence suggests a more complex Wnt3a profile, we simplify our biological model of independent Wnt3a decay by setting each **cell**'s Wnt3a concentration proportional to its level of *fgf8* mRNA:
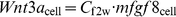
(6)We neglect Wnt3a secretion and diffusion both for computational simplicity, and because its very short diffusion length [9], [46] would effectively restrict it to the secreting **cell**'s immediate neighborhood. Setting the Wnt3a concentration proportional to the FGF8 protein concentration does not affect our results (data not shown). We make a similar simplification regarding Wnt3a to the one we make for FGF8: we take Wnt3a signaling to be proportional to the cellular concentration of Wnt3a, ignoring signal-receptor interaction and Wnt3a receptor saturation.

### Segmentation clock

In our ODE-based mathematical submodel of the segmentation clock, we modified the ODEs of Goldbeter and Pourquié's mathematical segmentation-clock model to reflect our changes to their biological model (see **Figure 3**). Supporting material **TextS1** presents the full set of ODEs in our segmentation-clock mathematical submodel. We neglect the numerous sources of intracellular fluctuations in real biological reaction networks, some of which we could implement at the computation level, *e.g.*, using a Gillespie method, both because they are computationally expensive to simulate and because the stochastic GGH model creates stochastic fluctuations that are large compared to the errors due to the ODE approximation. The lowest molecular concentrations dealt with in the extended clock model are of the order of 10^−4^ nM, corresponding to an average intermolecular distance of about 0.25 µm, two orders of magnitude lower than the average diameter of PSM cells (10 µm). In addition, such low concentrations occur for only a few molecular species and during only a fraction of the clock period, so we need not model the clock using stochastic methods at our current level of detail (see [75] for a discussion of when stochastic methods are necessary).

At the simulation level, we wrote a C++ class (**Oscillator**) to integrate the segmentation-clock network equations in each **cell** (**Text S2**). **Oscillator** stores current values for the molecular species and uses a fourth-order Runge-Kutta solver to integrate the equations for given values of local FGF8 signaling, local Wnt3a signaling and juxtacrine Delta signaling at each time step. We Python-wrapped **Oscillator** using *Simplified Wrapper and Interface Generator*, or *SWIG* (http://www.swig.org), to make it accessible in Python. Within a custom CC3D simulation class, we attached an instance of **Oscillator** to each **PSM cell** as a **cell** attribute. The class handles inputs from each **cell's** local environment (including surrounding **cells**) to the **cell's** instance of **Oscillator**, integrating the network equations and storing the values of the oscillating molecular species' concentrations for access by other simulation classes.

In a simulation of a single self-coupled **cell** (*i.e.*, the **cell** perceives incoming Delta signaling equal to its outgoing Delta signaling), our computational segmentation-clock submodel (**Figure 3**, **Text S1** and **Table S1**) produces oscillations in Lfng, Axin2 and Dusp6 with the qualitative phase relationships seen *in vivo*. When we simulate multiple segmentation-clock networks with different initial phases coupled via Delta/Notch signaling, they phase lock with the same phase, while maintaining the desired intracellular FGF-Wnt-Notch phase relationships (**Figure S3**). We do not study the effects of intercellular variability among segmentation clocks: the segmentation-clock submodels in each **cell** in our current integrated computational model all have identical parameters. We will present alternate networks, parameter sensitivity analyses and the effects of intercellular variability in segmentation-clock parameters in future work.

### Coupling the segmentation clock to the morphogen fields

At the biological level, FGF8 and Wnt3a interact with the FGFR1 and Frizzled receptors, respectively, leading to intracellular signal transduction in the FGF and Wnt pathways and driving the segmentation clock within a cell (see **Figure 3**). Thus, the local concentrations of FGF8 and Wnt3a potentially affect the amplitudes and/or oscillation periods of cells' segmentation clocks.

In our mathematical submodel of segmentation-clock-morphogen interaction, we simplify Wnt3a signaling by assuming that the concentration of disheveled (*Dsh*), which interferes with β-catenin phosphorylation as a downstream effect of signaling through Frizzled, is proportional to the degree of Wnt3a signaling. We further simplify Wnt3a and FGF8 signaling at the computational level, where we take the degree of FGF8 signaling within a **cell** to be equal to the FGF8 concentration at the center of the **cell** and the degree of Wnt3a signaling to be cell-autonomous.

**Figure 6:**
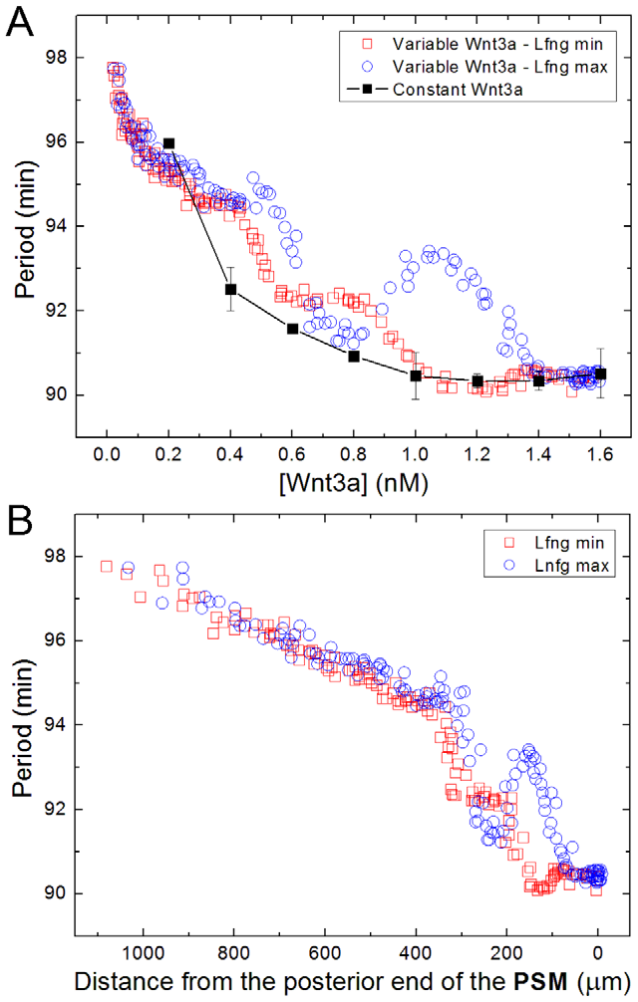
Segmentation-clock period versus Wnt3a concentration in simulated PSM. (**A**) Segmentation-clock period versus Wnt3a concentration in the simulated **PSM** (red squares and blue circles) and for **cells** with a constant Wnt3a concentration (connected black squares with error bars). (**B**) Segmentation-clock period as a function of **cell** position along the AP axis, measured by the anterior distance from the posterior (right) end of the simulated **PSM**. Slower oscillations in the anterior (left) simulated **PSM** are consistent with similar observations *in vivo* [77]. Red squares indicate the period measured between times of minimum Lfng concentration and blue circles indicate the period measured between times of maximum Lfng concentration. Parameters are the same as in the reference simulation (**Figure 7**). For more information see **METHODS** **: Coupling segmentation clock to the morphogen fields**.

We find that the period of segmentation-clock oscillations increases with decreasing Wnt3a both in the simulated **PSM** and in sets of simulations of **cells** exposed to different, but constant, concentrations of Wnt3a (**Figure 6** (**A**)). In the case of the simulated **PSM**, this effect results in an anteriorly-increasing segmentation-clock period (**Figure 6** (**B**)), consistent with observations *in vivo* of segmentation-clock oscillations slowing within cells as they approach the anterior of the PSM [76], [77]. In the regime where the model segmentation-clock network produces stable oscillations (for all but very low FGF8 and/or Wnt3a concentrations), the segmentation-clock period is independent of the FGF8 concentration (not shown), which is also consistent with experimental observations that the segmentation-clock period appears FGF8-independent [76].

### Clock-wavefront readout

In our biological model, three proteins serve as intermediaries between the segmentation-clock-wavefront interaction and eventual expression of adhesion proteins prior to and during somite formation: cMeso (Mesp2), downstream of Notch; cytoplasmic β-catenin moderated by Wnt3a signaling; and Paraxis, downstream of Wnt3a/β-catenin signaling (see **Figure 4** (**A**)). Because the exact regulation of Mesp2 and Paraxis is unknown, we do not explicitly mathematically or computationally model their expression. We correlate the activity of each one with the concentration of an oscillatory component within our segmentation-clock submodel that is plausibly under similar regulation. We correlate cMeso (Mesp2) activity with Lfng concentration, as both are downstream of active Notch signaling and both repress Notch signaling (see **Figure 3** and **Figure 4**). Similarly, we correlate Paraxis activity with Axin2 concentration because both are downstream targets of Wnt/β-catenin signaling (see **Figure 3** and **Figure 4**). We have dual motivations for this strategy. It is consistent with our simplified biological submodel, and the choice of the Lfng/Axin2/β-catenin trio allows us to take advantage of a convenient characteristic of the time-series behavior of the segmentation-clock submodel.

For external FGF8 and Wnt3a concentrations close to their values at the determination front, a **PSM cell**'s Lfng, Axin2 and β-catenin concentrations form temporally distinct peaks, allowing us to express our biological submodel of determination as a simple Boolean readout that assigns each **PSM cell** a determined **cell type** at the determination front (**Figure 4**). When the FGF8 concentration drops below the determination threshold, we determine whether the **cell** belongs in the core of the **somite** by comparing the concentration of β-catenin to a semi-arbitrary threshold for N-cadherin stabilization. Above this threshold, the **cell** chooses a **Core cell** fate; otherwise, the **cell** chooses a **peripheral cell** fate. **Peripheral cells** with [Lfng]>*k_1_*[Axin2] choose anterior compartment (**EphA4**) fates, while those with [Lfng]≤*k_1_*[Axin2] choose posterior compartment (**ephrinB2**) fates (see **Figure 2** and **Figure 4** (**B**)).

### Differentiation

We implement our simplified submodel of two-step differentiation by reassigning new **cell types** to **cells** in the modeled **PSM** twice in the course of a simulation. At determination, we reassign **PSM cells** to **pre_EphA4**, **pre_ephrinB2** or **pre_Core cell types** according to the criteria shown in **Figure 4** (**B**). Determined **cell types** have adhesion properties that are intermediate between **PSM** and **somitic cell types** (see **Table 3**). Later, at differentiation, the determined **cells** change their **cell types** to **EphA4**, **ephrinB2** or **Core**, which have the adhesion properties that drive **somite** formation and maintenance (see **Table 3**).

In the reference simulations (and where not otherwise stated), we implement the cell-autonomous delay submodel of differentiation by attaching a “ticker” attribute to each **cell** at determination and incrementally increasing its value until four segmentation-clock periods have elapsed, at which time we reassign the **cell** a **somitic** cell type. Setting a second FGF8 concentration threshold to position the differentiation front four somite lengths anterior to the determination front in lieu of a differentiation “ticker” does not significantly alter our results (not shown).

To explore the dynamic patterns of Lfng expression in the simulated **PSM**, we compared the cell-autonomous delay submodel to the positional differentiation-front model. Instead of modeling the RA concentration field explicitly to implement the positional differentiation-front submodel, we make the simplifying assumption that once the **PSM** reaches its full length, the differentiation front begins at the anterior tip of the **PSM** and advances at a constant speed equal to the **PSM** growth rate, maintaining the **PSM** at a constant length. We make this simplification because modeling the detailed mechanisms that position the differentiation front would not impact the simulated Lfng expression patterns at the level of detail we consider in our investigations.

## Results

### Reference simulations reproduce key features of wild-type somitogenesis *in vivo*

Simulations of our integrated model produce a single irregular **somite** (reflecting the initial conditions, see **Figure 5**) after the **PSM** first reaches its full length. Then, at the frequency of the segmentation clock (as measured in the posterior **PSM** where the frequency is greatest), our model forms an unlimited series of **somites** with consistent size, shape, and anterior, core and posterior compartments. **Videos S1**, **S2** and **S3** show a typical reference simulation visualizing **cell types**, extracellular FGF8 concentration and intracellular Lfng concentration, respectively. **Video S4** shows an image of a longer-than-typical simulation, demonstrating the reference model's ability to steadily produce an unlimited number of **somites**.

**Figure 7:**
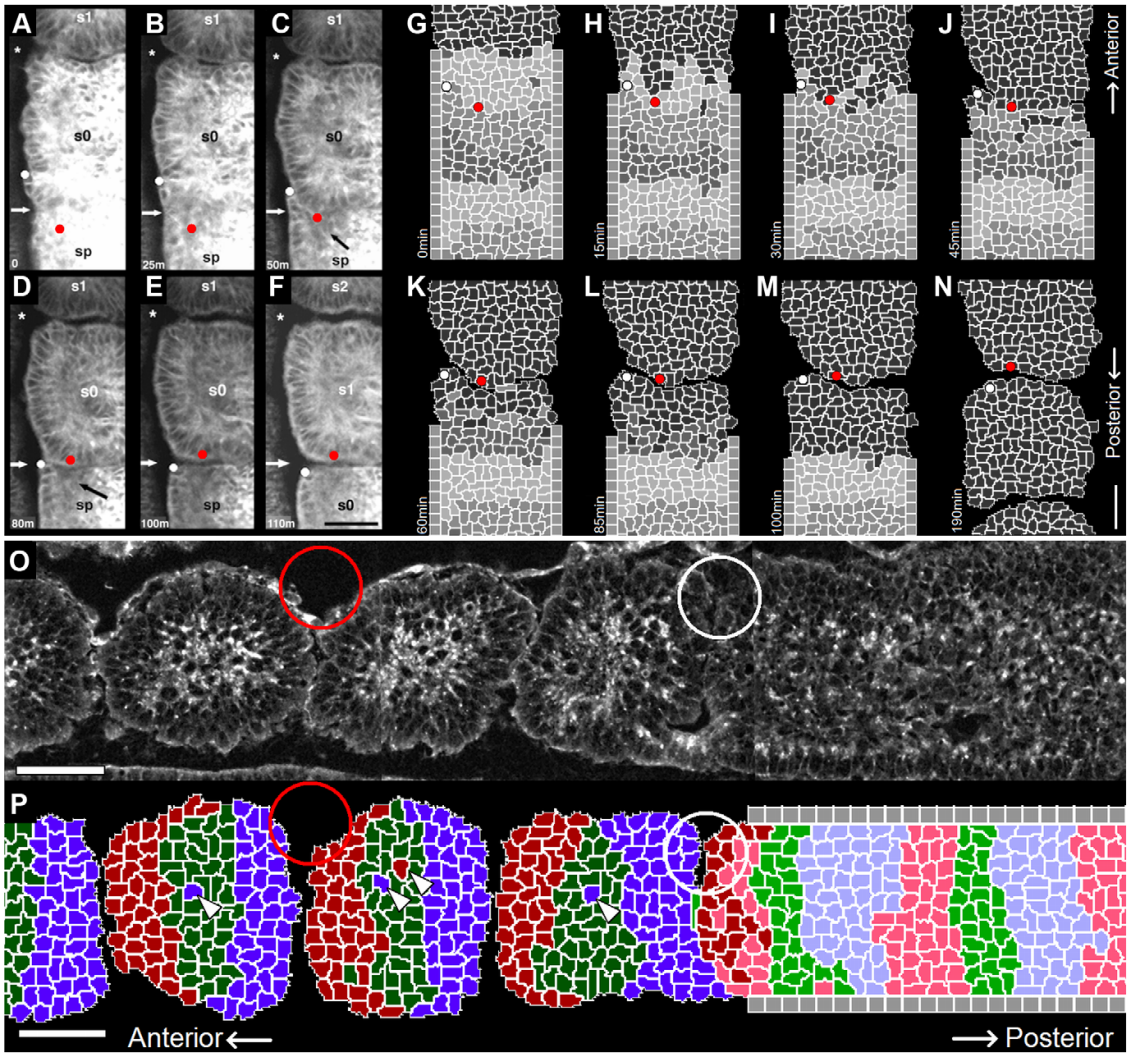
Comparison of reference simulation results with *in vivo* observations. (**A–F**) Experimental images from Kulesa and Fraser [2], taken at 0, 25, 50, 80, 100 and 110 minutes (reproduced with authorization). Scale bar 50 µm. (**G**–**M**) Snapshots of a simulation reproducing the “ball and socket” morphology described by Kulesa and Fraser [2], taken at 0, 15, 30, 45, 60, 85, 100 and 190 minutes. Scale bar 40 µm. Initially, a “sleeve” of cells that will eventually be posterior to the forming border cradles presumptive somite cells that will eventually be anterior to the forming border (**A–C**, **G–J**). As the intersomitic border continues to develop, these two populations of cells move relative to each other to position themselves on the appropriate sides of the border (**D–E**, **K–M**). The “sleeve” then retracts, leading to a rounded intersomitic border (**F**, **N**). The white and red dots in the simulations correspond to those in the experimental images. (**O**) Confocal image of one half of the PSM in a live chick embryo at HH Stage 10, stained with the cell-surface lipid label BODIPY ceramide. (**P**) Simulation detail at the corresponding time point. Simulated morphology closely resembles that observed *in vivo*, including the initially narrow gap separating adjacent somites (white circles), the block-like shape of the newly forming somite, the gradual rounding of more mature somites, and the resulting notch-like intersomitic clefts at the medial and lateral edges of maturing somites (red circles). Another notable feature of the simulation is the persistence of misplaced **cell** types after differentiation (white arrow heads). Model **cell** type colors are identical to those in **Figure 5**. Scale bars 50 µm. Reference simulation parameters: segmentation-clock period = 90 min; **PSM** growth rate = 1.63 µm/min; **Table 4** (FGF8 and Wnt3a); **Table 3** (cell-cell adhesion); **Table 2** (**cell** sizes and motility); and **Table S1** (segmentation clock).

Simulated (*in silico*) avian **PSM** and **somite** tissue morphologies closely resemble those *in vivo* (**Figure 7**). In both, somites are initially block-like and gradually round up as they mature. A gap that is narrow at the center line and more pronounced and notch-shaped at the medial and lateral edges separates adjacent somites. The notch widens as the somites mature.

Because our model omits epithelialization, ML asymmetry, recruitment of **Core cells** to the **somite** border and three-dimensional effects, the detailed organization and shape of **cells** in the simulated **somites** differ slightly from somitic cells *in vivo*. Segmentation, border formation and border maturation in the simulations, which include only adhesion-related changes in **cell** morphologies, however, closely resemble those *in vivo*, suggesting that our model captures the primary mechanisms of somitogenesis and that these omitted effects play more limited roles in the modeled stages of somitogenesis. Our model's ability to control and assay the importance of individual biological mechanisms is particularly useful for understanding the mechanisms of somitogenesis, because such control is lacking experimentally, *e.g.*, separating adhesion from epithelialization *in vivo* is difficult because Eph-ephrin signaling, which is responsible for cell-cell repulsion at the somite border, is also implicated in epithelialization [22].

In chick embryos, the cells belonging to the forming somite and the anterior PSM intermingle across the presumptive intersomitic border, so the intersomitic border does not initially form a smooth ML line. Kulesa and Fraser [2] reported that PSM cells at the ML edges and center of the presumptive intersomitic border are initially several cell diameters anterior to the eventual intersomitic border, while presumptive somite cells between the ML edges and center of the forming border are initially posterior to the eventual intersomitic border, forming a distinct “W” shape. As the border matures, the two cell populations separate from one another, causing the border to smooth and flatten (**Figure 7** (**A**–**F**)).

In our model, intrinsic noise due to **cell** motility initially generates **cell** mixing across the presumptive somite border. As a rule, this mixing is laterally homogeneous, sometimes resulting in the border shape described by Kulesa and Fraser [2] and sometimes resulting in other patterns (see **Video S1**). Adhesion-driven **cell** sorting and border smoothing follow determination, forming clear intersomitic gaps and rounded **somites** despite initial intermingling of **cells** across the border. In cases where the initial pattern of mixing across the border resembles that described by Kulesa and Fraser, the ensuing morphological events are also similar (**Figure 7** (**G**–**N**)), suggesting that, while our model does not incorporate all of the mechanisms responsible for the initial pattern of cell determination/differentiation at the presumptive intersomitic gap *in vivo*, it does include plausible mechanisms for producing the ensuing cell migrations.

**Figure 8:**
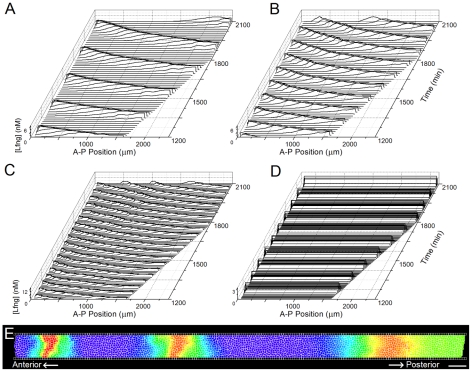
Anteriorly traveling Lfng stripes and segmentation-clock period. (**A**–**C**) Lfng expression versus AP position and time for different segmentation-clock periods. (**A**) Increasing the segmentation-clock period to 180 min from the reference simulation period of 90 min decreases the spatial and temporal frequency of Lfng stripes compared to the reference simulation (**B**). (**C**) Decreasing the segmentation-clock period to 45 min increases the spatial and temporal frequency of Lfng stripes compared to the reference simulation ([Lfng] axis rescaled for clarity). (**D**) For a uniform Wnt3a concentration of 0.5 nM, **cells**' segmentation-clocks oscillate in phase with a period of 90 min. (**E**) Lfng concentration in a simulation with a segmentation-clock period of 45 min. The distance between the center and anterior (left) peaks is shorter than the distance between the center and posterior (right) peaks. Scale bar 40 µm. Parameters, when not otherwise noted, are equal to those in the reference simulation (**Figure 7**). The color scale is the same as that in **Figure 5** (red indicates high concentration of Lfng and blue low concentrations of Lfng). We increase or decrease the segmentation-clock period by varying how long we integrate the segmentation-clock ODEs during each time step; by doing so, we easily vary the clock period relative to other processes in the simulation without altering parameters within the segmentation-clock submodel or changing the clock response to FGF8, Wnt3a or Delta/Notch signaling. For more information see **RESULTS** **: Reference simulations reproduce key features of wild-type somitogenesis** ***in vivo***, **The number of high Lfng concentration stripes in the simulated PSM depends on the segmentation-clock period, PSM growth rate and PSM length** and **Somites form** ***in silico*** **in the absence of traveling gene expression stripes**.

Glazier *et al.* [7] studied the effect of noisy initial border specification on somitogenesis in an adhesion-driven model with six model somitic cell types and achieved similar results (Figures 8 and 9 of [7]). Whereas Glazier *et al.* specified noisy presumptive borders as an initial condition, in our model noise arises from **cell** mixing prior to differentiation. Our model's ability to reproduce realistic dynamic border morphology with only three **somitic cell types** in place of the six somitic cell types used by Glazier *et al.* indicates that adhesion-mediated cell sorting can explain somite gap formation and somite rounding even in the simplest case of repulsive anterior and posterior compartments and a highly adhesive core connecting the two within a somite, strengthening the argument for a differential adhesion-driven model of somite formation.

**Figure 9:**
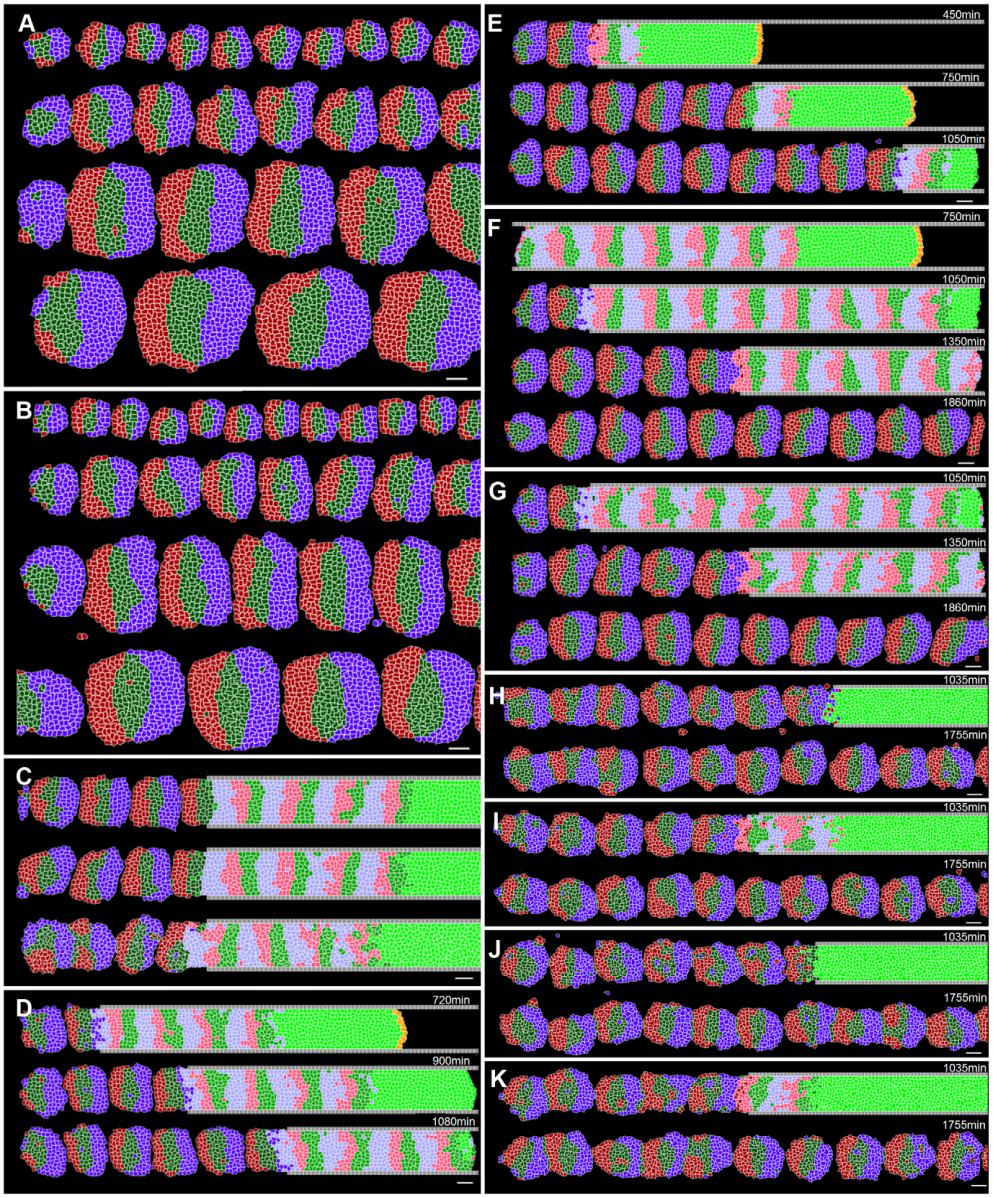
Results of *in silico* perturbation experiments. (**A**) *In silico* somite formation for different segmentation clock periods. From top to bottom, *T*_clock_ = 67.5 min, 90 min (reference), 135 min, 180 min. (**B**) *In silico* somite formation for different **PSM** growth rates. From top to bottom, growth rate = 1.08 µm/min, 1.63 µm/min (reference), 2.04 µm/min, 2.72 µm/min. In (**A**) and (**B**), well-formed smaller **somites** (top of each panel) require decreased **cell** motility (for **PSM cells**, *λ*_surf_ = 20 and *D*_cell_ = 0.945 µm^2^/min in (**A**); *λ*_surf_ = 25 in (**B**)); larger **somites** form for reference motility parameters. In each case, we adjust the ML dimension to produce roughly circular **somites**. Segmentation and **somite** separation, however, succeed both for smaller and larger ML widths (data not shown). (**C**) *In silico* somite formation for different values of **cell** motility parameter *λ*_surf_. From top to bottom, low **cell** motility (*λ*_surf_ = 25, *D*_cell_ = 0.86 µm^2^/min), reference motility (*λ*_surf_ = 15, *D*_cell_ = 1.08 µm^2^/min), high motility (*λ*_surf_ = 5, *D*_cell_ = 1.76 µm^2^/min). For low motility, **somites** round up slowly and there is little **somite** shape variation compared to reference simulations. For high motility, excessive mixing of **cell** types across presumptive **somite** borders leads to fused **somites**. (**D**) *In silico* somitogenesis with a uniform Wnt3a concentration. When [Wnt3a] is uniform throughout the **PSM**, traveling Lfng stripes do not form, but segmentation is normal, demonstrating that traveling stripes of high protein concentration are not necessary for somitogenesis in our model. (**E**) *In silico* somitogenesis for shorter-than-normal determination-differentiation delay (90 min); from top to bottom, *t* = 450 min, 750 min, 1050 min. (**F**) *In-silico* somitogenesis for longer-than-normal determination-differentiation delay (720 min); from top to bottom, *t* = 750 min, 1050 min, 1350 min, 1860 min. (**G**) *In silico* somitogenesis for long determination-differentiation delay (720 min) and less pronounced **cell** adhesion changes at determination. Modified contact energies: *J_pre_EphA4,pre_EphA4_* = −22; *J_pre_ephrinB2,pre_ephrinB2_* = −22; *J_pre_Core,pre_Core_* = −25; *J_pre_EphA4,EphA4_* = −22; *J_pre_ephrinB2,ephrinB2_* = −22. Increased mixing of determined **cell** types is corrected by **cell** sorting after differentiation. (**H–K**) *In silico* somitogenesis for delayed adhesion changes after determination with and without a period of intermediate adhesion before differentiation. (**H**) 180-min determination-differentiation delay and no intermediate adhesion. (**I**) 360-min determination-differentiation delay with a 180-min period of intermediate adhesion after 180 min of unchanged adhesion. (**J**) 225-min determination-differentiation delay and no intermediate adhesion. (**K**) 360-min determination-differentiation delay with a 135-min period of intermediate adhesion after 225 min of unchanged adhesion. For a determination-differentiation delay of 180 min or greater and no period of intermediate adhesion, the excessive mixing of determined **cell** types across their original borders leads to fused **somites** and a greater-than-normal occurrence of stranded **Core cells** in the intersomitic gaps (**H**, **J**). A period of intermediate adhesion after such a period of **cell** mixing partially corrects resulting defects (**I**, **K**). With and without a period of intermediate adhesion, defect severity increases with increasing periods of **cell** mixing. All panels: anterior to the left, scale bars 40 µm, **cell-type** colors same as **Figure 5**, parameters have reference values (**Figure 7**) unless otherwise noted. For greater detail and resolution, see **Supporting Figures S5, S6, S7, S8, S9, S10, S11**.

One feature of our simulations that has not been extensively studied *in vivo* is the persistence of dislocated cell types well into somitogenesis (**Figure 7** (**P**)). This feature distinguishes cell-sorting-driven correction of “fuzzy borders” from possible mechanisms that either re-differentiate or kill misplaced cells, and thus stands as a prediction of the submodel of differential-adhesion-mediated cell-sorting as the primary border-correction mechanism.

The model also produces traveling stripes of Lfng expression that are similar to those observed *in vivo*. In the presence of a Wnt3a gradient, stripes of high Lfng concentration appear to form in the posterior **PSM** and travel in the anterior direction, narrowing in the AP direction as they do (**Figure 8** (**E**)). Lfng stripes did not occur for constant Wnt3a signaling, in either the presence or absence of an FGF8 gradient (**Figure 8** (**D**)). We would expect this lack of response to variations in FGF8 given the insensitivity of the segmentation-clock period to the FGF8 concentration.

Our results are consistent with descriptions of the characteristic traveling stripes of gene expression as pseudo-waves arising from an AP gradient of the segmentation-clock periods in the PSM [37], [76], [78], rather than as propagating waves or the result of a conserved phase offset. **PSM cells** in the computational model inherit their segmentation-clock phases from their parent **Source cells**. Because our reference simulations begin with the segmentation clocks in all **Source cells** in phase with one another, in the absence of any additional influences all **cells'** segmentation clocks would oscillate in phase and Lfng concentration would be spatially uniform throughout the **PSM** (as is the case for constant Wnt3a signaling, see **Figure 8** (**D**)). Thus, the pseudo-waves of Lfng in our simulations must result from interactions with factors external to individual segmentation-clock networks. This factor is the Wnt3a signal which, while external to the segmentation-clock network, is internal to the **cell**. Simulations in which the segmentation clocks run cell-autonomously without Delta/Notch coupling between neighboring **cells** produce traveling Lfng expression stripes similar to those in simulations with **cell-cell** coupling, although noisier in the anterior since they lack **cell-cell** Delta/Notch coupling to compensate for **cell** mixing (**Video S5**). Such cell-autonomous stripe behavior could not occur for our initial conditions were the stripes propagating waves. Our integrated model differs from models of travelling waves [37], [72], [78], [79] where the period-/phase-altering factor is either completely external to the cells or imposed as a gradient of an internal parameter.

Because our integrated model combines multiple submodels embodying biological assumptions at different scales, it has many parameters (the regulatory network has about a hundred). Although an exhaustive sensitivity analysis for all parameters would be extremely time-consuming, we investigated the effects of significant variations of the main parameters and processes. We summarize our results in **Table 9**. Here, we describe the results of a series of *in silico* experiments in which we varied key and novel parameters, keeping fixed most of the parameter values that we have directly imported from previous studies, *e.g.*, the transcription/translation rates in the Goldbeter-Pourquié model [16].

### The segmentation-clock period and PSM growth rate regulate somite size

Species vary greatly in their size and number of somites (**Table 6**). The clock-and-wavefront model can produce variation in somite size by varying the period of the segmentation clock and/or the speed of the advancing wavefront. Because the clock-and-wavefront model can continue to produce somites indefinitely, the number of somites is determined primarily by factors in the tailbud external to the PSM.

**Table 6:** Properties of somitogenesis during the modeled stages by species.

	Zebrafish	Mouse	Chicken	Corn snake
**AP somite length**	50 µm	115 µm	150 µm	40 µm
**Segmentation-clock period**	30 min	120 min	90 min	100 min
**PSM growth rate (AP)**	2.49 µm/min	0.96 µm/min	1.66 µm/min	0.47 µm/min
**PSM length**	600 µm	1100 µm	1400 µm	1200 µm
**Number of somites**	31	65	55	315±10%

Values estimated from Figure 4 in [77]. The AP somite length and PSM growth rate are for stage HH 12+ (17 out of 52 somite pairs) in chicken and at the stages corresponding to the same fraction of total somites formed in zebrafish, mouse and corn snake. For more information see **RESULTS** **: The segmentation-clock period and PSM growth rate regulate somite size** and **The number of high Lfng concentration stripes in the simulated PSM depends on the segmentation-clock period, PSM growth rate and PSM length**.

We manipulated the period of the segmentation clock in our simulations by varying how frequently we updated the clock per unit time (*i.e.*, the number of clock iterations per MCS); we chose this method in order to predictably alter the segmentation-clock period without changing internal clock parameters or the clock's response to FGF8, Wnt3a or Delta/Notch signaling. Characteristics such as the clock's sensitivity to Wnt3a signaling and association constants between interacting clock components influence the clock period in a more biologically realistic way, but are less predictable in their additional influences on clock behavior. We manipulated the progression of the FGF8 determination front by altering the rate of **PSM** growth.

Predictably, decreasing the segmentation-clock period decreases **somite** size and increasing the segmentation-clock period increases **somite** size (**Figure 9** (**A**) and **Figure S5**) [19]. If the rate of wavefront progression is unchanged, then the wavefront will travel a smaller distance during a shorter segmentation-clock period and a larger distance during a longer segmentation-clock period.

Because the FGF8 gradient is produced by gradual intracellular fgf8 mRNA decay, rather than extracellular protein diffusion, increasing the rate of **PSM** growth “stretches” the shape of the FGF8 gradient (see **Figure S4**) and thus decreases the speed of the advancing determination wavefront. If the segmentation-clock period is unchanged, then a slowly progressing wavefront will travel a smaller distance during a segmentation-clock period and result in smaller **somites**, and a quickly progressing wavefront will travel a greater distance over a segmentation-clock period and result in larger **somites** (**Figure 9** (**B**) and **Figure S6**).

**Figure 10:**
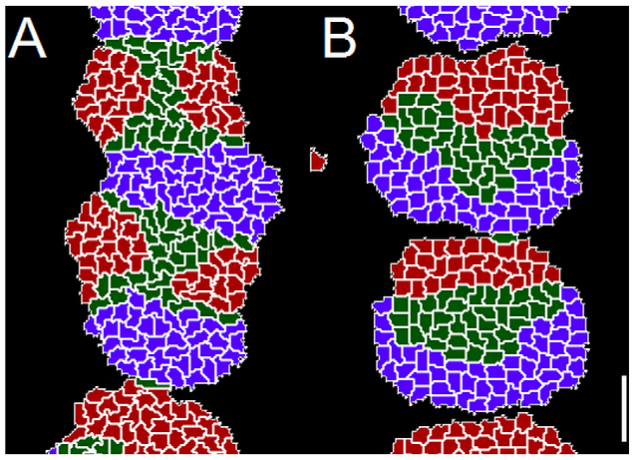
Somitogenesis defects. (**A**) A group of **Core cells** breaks through an adjacent EphA4 or ephrinB2 compartment, leading to fused **somites**. **Somite** fusing is a defective phenotype that does not occur in normal *in vivo* or *in silico* somitogenesis. (**B**) A single **Core cell** is stranded in the naturally acellular perisomitic ECM. Such stranded cells occasionally occur in normal *in vivo* somitogenesis. **Cell** colors are the same as in **Figure 5**. Scale bar 40 µm. For more information see **RESULTS** **: The segmentation-clock period and PSM growth rate regulate somite size**.

Well-defined larger **somites** formed without further adjustments to the reference simulation parameters, but formation of smaller **somites** with well-defined borders and clean intersomitic gaps required smaller **cell** motility than the reference simulation. **Cell** mixing easily disrupts extremely narrow compartments of **pre_EphA4**, **pre_ephrinB2** and **pre_ Core cells**, interfering with future apposition of **EphA4** and **ephrinB2 cell** compartments after differentiation. If a continuous cluster of **pre_Core cells** breaks through an adjacent compartment of **pre_EphA4** or **pre_ephrinB2 cells**, adhesion-mediated border correction, gap formation and compartment maintenance fail and the **somites** fuse (**Figure 10** (**A**)). For large **somites**, if a single **pre_Core cell** or a small cluster of **pre_Core cells** breaks through a neighboring compartment into the future intersomitic gap, then the two **somites** will be joined by the **Core cells**, which will prevent complete formation of the intersomitic gap, but the two joined **somites** will otherwise form normally and maintain their intrasomitic compartments (**Figure 10** (**B**)). For small **somites**, even a single breakthrough **pre_Core cell** may be enough to fuse adjacent **somites**, allowing us to predict that cell motility will be lower compared to the segmentation-clock period in species with very small somites than in species with large somites.

Our ability to control simulated **somite** size and formation rate by adjusting distinct mechanisms *in silico* allows us to explore the plasticity and robustness of the modeled somitogenesis mechanisms and provides a convenient tool for exploring the development and evolution of interspecies variability.

### The number of high Lfng concentration stripes in the simulated PSM depends on the segmentation-clock period, PSM growth rate and PSM length

In addition to affecting **somite** size, varying the segmentation-clock period and morphogen distribution changes the patterns of segmentation-clock protein and mRNA concentrations across the **PSM** *in silico*. *In vivo*, the number of high-Lfng-concentration stripes simultaneously present in the PSM varies between species, ranging from one or two in chick to as many as nine in the corn snake [77].

To explore the relationship between segmentation-clock period, PSM growth rate and number of Lfng stripes in the PSM, we first visualized Lfng concentration in simulations with a fixed rate of **PSM** growth and varying segmentation-clock periods (see **Figure 8**). As expected, faster segmentation clocks produce greater numbers of stripes than slower clocks. When multiple stripes are present simultaneously, the distance between stripes narrows as the stripes move anteriorly down the **PSM**, as seen in experiments. In conjunction with the segmentation-clock period gradient along the **PSM** (increasing anteriorly along the tissue), this observation supports the methods Gomez *et al.* and Giudicelli *et al.* [77], [80] used to calculate the segmentation-clock period at different positions in experimental tissues.

Next, we varied the **PSM** growth rate while holding the segmentation-clock period fixed. We did so in simulations implementing both the cell-autonomous differentiation and positional differentiation front submodels.

**Figure 11:**
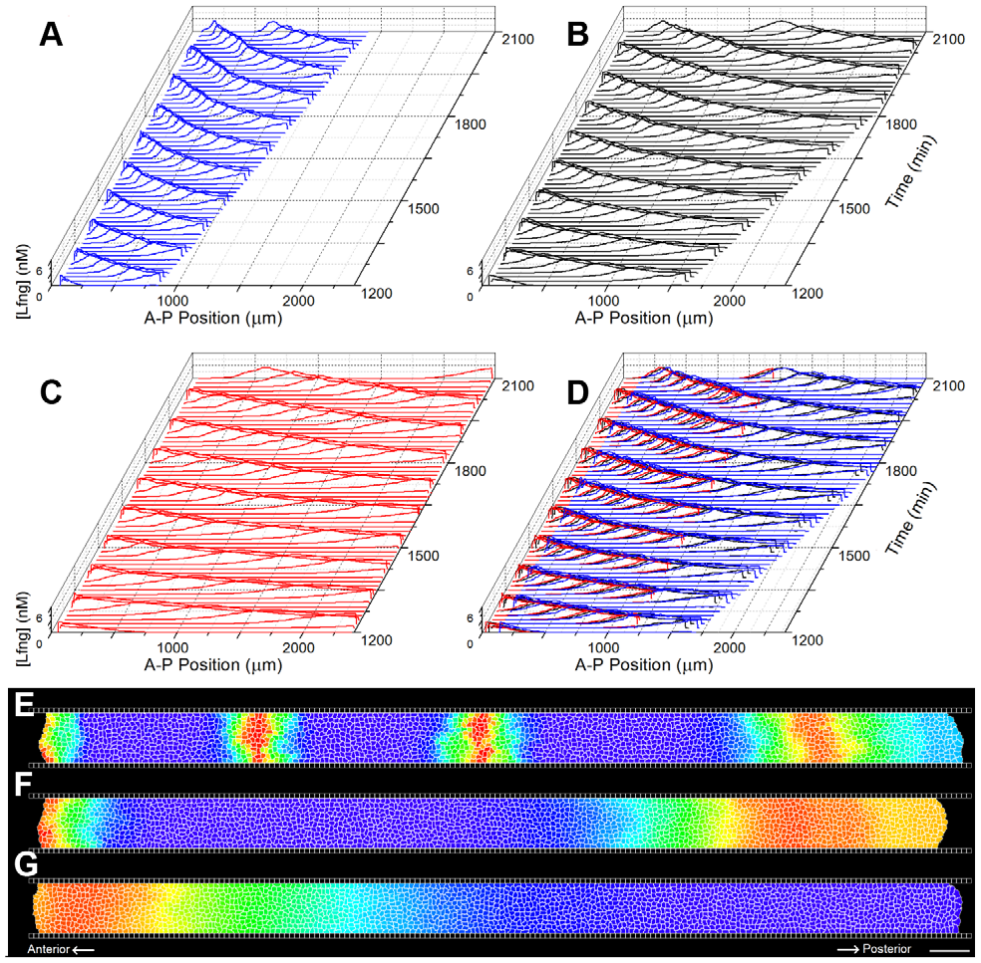
Lfng expression in simulated PSM for different PSM growth rates. (**A–D**)The number of *in silico* Lfng stripes in the **PSM** is independent of the **PSM** growth rate for fixed segmentation-clock period and minimum (anterior) concentration of FGF8. Faster/slower **PSM** growth stretches/compresses the Wnt3a profile, stretching/compressing the Lfng concentration stripes. (**A**) Slow **PSM** growth rate ( = 0.82 µm/min). (**B**) Reference simulation (**PSM** growth rate = 1.63 µm/min). (**C**) Fast **PSM** growth rate ( = 3.27 µm/min). (**D**) Rescaling the length of the **PSM** in (**A**) and (**C**) to match the reference simulation in (**B**) demonstrates that the three cases are equivalent after accounting for the expansion or compression of the Wnt3a gradient. (**E–G**) The number of *in silico* Lfng concentration stripes in the **PSM** depends on the **PSM** growth rate for a fixed segmentation-clock period and **PSM** length. When the **PSM** length, rather than the minimum (anterior) FGF8 concentration, is fixed, faster/slower **PSM** growth decreases/increases the change in Wnt3a concentration between the posterior and anterior ends, decreasing/increasing the number of Lfng concentration stripes in the **PSM**. (**E**) Slow **PSM** growth rate ( = 0.82 µm/min). (**F**) Reference simulation (**PSM** growth rate = 1.63 µm/min). (**G**) Fast **PSM** growth rate ( = 3.27 µm/min). Anterior to the left. Scale bar 80 µm. The color scale is the same as that in **Figure 5** (red indicates high concentration of Lfng and blue low concentrations of Lfng). Parameters, when not otherwise noted, are equal to those in the reference simulation (**Figure 7**). For more information see **RESULTS** **: The number of high Lfng concentration stripes in the simulated PSM depends on the segmentation-clock period**.

Because the shapes of the FGF8 and Wnt3a concentration gradients depend on intracellular mRNA decay rather than on the protein diffusion lengths [9], [20], [45], if the parameters governing FGF8 and Wnt3a production and decay are unchanged, faster simulated **PSM** growth stretches the Wnt3a and FGF8 concentration gradients along the AP axis relative to the reference simulation, while slower **PSM** growth compresses the FGF8 and Wnt3a concentration gradients. In a cell-autonomous differentiation model, the minimum (anterior) concentration of Wnt3a (which is cell-autonomous in our model and nearly cell-autonomous *in vivo*) is unchanged from the reference simulation, so when the AP position is normalized by the **PSM** length, the Wnt3a concentration gradient is unchanged (**Figure S4**). Changing the AP Wnt3a concentration gradient correspondingly changes the patterns of Lfng concentration: faster **PSM** growth broadens stripes of high Lfng concentration and slower **PSM** growth narrows them, leaving the number of Lfng stripes unchanged (**Figure 11** (**A–D**)).

When we fix the length of the **PSM** and allow the minimum (anterior) concentrations of FGF8 and Wnt3a to vary with the **PSM** growth rate, slower **PSM** growth increases the number of Lfng stripes compared to the reference simulation and faster **PSM** growth decreases the number of Lfng stripes compared to the reference simulation (**Figure 11** (**E–G**)), consistent with the *in vivo* observations of Gomez *et al.* [77] that the number of Lfng stripes in the PSM depends on the segmentation-clock period relative to the PSM growth rate and that PSM length is comparable between organisms with significantly different growth rates (see **Table 6**).

Gomez *et al.* estimated that the number of high Lfng concentration stripes should be proportional to the ratio of the length of the PSM to the somite size, given a conserved relationship between the segmentation-clock period and the AP position as a fraction of the total PSM length. In our model, in which the **PSM** increases its AP length exclusively through **cell** addition at the posterior end rather than through uniform expansion of the PSM due to cell proliferation throughout the tissue, the relationship between the fractional AP position and the segmentation-clock period is not conserved when the **PSM** length is constant and the **PSM** growth rate is varied. However, even without changing the parameters governing FGF8 and Wnt3a production, diffusion or decay (*i.e.*, without changing the relationship between the absolute AP position and the segmentation-clock period), our model predicts that the number of high Lfng concentration stripes in the PSM will increase with the ratio of PSM length to somite size.

These observations, summarized in **Table 7**, have implications regarding how the differentiation front is positioned *in vivo*. If the FGF8 concentration defines the differentiation front independently of other factors, then the length of the PSM depends only on the PSM growth rate, and the number of Lfng stripes will be independent of the growth rate. Additional FGF8 antagonists or differentiation promoters, *e.g.*, RA, however, might allow both slow and fast PSM growth to produce a PSM of the same length, in which case a more slowly growing PSM will have more simultaneous Lfng stripes (**Figure 11**).

**Table 7:** Dependence of the number of Lfng stripes in the modeled **PSM** on the **PSM** growth rate and segmentation-clock period.

PSM length	PSM growth rate	Minimum (anterior) FGF8 and Wnt3a	Segmentation-clock frequency	Number of simultaneous Lfng stripes
Reference	Reference	Reference	High	Increased
Reference	Reference	Reference	Low	Decreased
Variable	High	Reference	Reference	Reference
Variable	Low	Reference	Reference	Reference
Reference	High	Variable	Reference	Decreased
Reference	Low	Variable	Reference	Increased

“Reference” indicates that the value is the same as in the reference simulation; “Variable” indicates that the value is free to change in response to changes in other factors; “High” and “Low” indicate imposed changes; “Increased” and “Decreased” indicate results for imposed changes. All are relative to the values in the reference simulation.

For more information see **RESULTS** **: The number of high Lfng concentration stripes in the simulated PSM depends on the segmentation-clock period, PSM growth rate and PSM length**.

Simulations in which the **PSM** length is fixed are more consistent with *in vivo* observations that Lfng stripe number depends on the PSM growth rate [77] than simulations in which differentiation is cell-autonomous or nearly cell-autonomous, suggesting that at least one additional signal besides FGF8, probably RA, positions the differentiation front and maintains a constant PSM length.

We will investigate the interplay between external signaling, PSM growth rate and the segmentation clock in more detail in future work.

### Cell motility affects somite border formation and morphology

In avians, experimentally-observed cell motility is much greater in the tailbud and posterior PSM than in the more anterior regions of the PSM, and is graded from high in the posterior PSM to low in the anterior PSM [25]. We simplified our model by assuming that all **PSM cells** in the modeled region have the same motility. To examine the effect of global **cell** motility on **somite** formation in our model and to assess whether a refined model should include variable **cell** motility, we varied the parameter *λ*_surf_, which changes the motility of the **cells**, as measured by their diffusion rate *D*_cell_ (see **Table 8** and **METHODS**). In all simulations, **PSM cells** subdiffuse, *i.e.*, their mean square displacement versus time 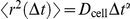, has an exponent *α* lower than that expected for a pure random walk (*α* = 1).

**Table 8:** Measured diffusion of **PSM cells** for different values of 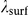.

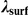	Cell diffusion constant (*D*_cell_)	Diffusion exponent (*α*)
5	1.76 µm^2^/min	0.91
15	1.08 µm^2^/min	0.79
25	0.86 µm^2^/min	0.73

For more information see **METHODS** **: Cell motility** and **RESULTS** **: Cell motility affects somite border formation and morphology**.

**Table 9:** Summary of simulation results for different mechanisms.

	Parameters						Results				
Case number	Adhesion strength of determined cell types	Determination-differentiation mechanism	FGF8 threshold	Distance between differentiation and determination	Wnt3a	PSM cell motility	Delta/Notch coupling	Intersomitic gap formation	Border correction	Compartment borders	Traveling Lfng stripes
1	Weak	Time delay	–	1 period	Gradient	Low	Y	Complete	N	Rough	Y
2	Weak	Time delay	–	1 period	Gradient	Reference	Y	Good	Y	Rough	Y
3	Weak	Time delay	–	1 period	Gradient	High	Y	70% fused	N	Very rough	Y
4	Weak	Time delay	–	4 periods	Gradient	Low	Y	Complete	Some	Rough	Y
5	Weak	Time delay	–	4 periods	Gradient	Reference	Y	Good	Y	Rough	Y
6	Weak	Time delay	–	4 periods	Gradient	High	Y	80% fused	N	Very rough	Y
7	Weak	Time delay	–	8 periods	Gradient	Low	Y	Complete	Some	Rough	Y
8	Weak	Time delay	–	8 periods	Gradient	Reference	Y	Good	Y	Rough	Y
9	Weak	Time delay	–	8 periods	Gradient	High	Y	90% fused	N	Very rough	Y
10	Strong	Time delay	–	1 period	Gradient	Reference	Y	Good	Some	Rough	Y
11	Strong	Time delay	–	4 periods	Gradient	Low	Y	Complete	N	Rough	Y
**Ref**	**Strong**	**Time delay**	**–**	**4 periods**	**Gradient**	**Reference**	**Y**	**Good**	**Some**	**Rough**	**Y**
12	Strong	Time delay	–	4 periods	Gradient	High	Y	40% fused	Some	Very rough	Y
13	Strong	Time delay	–	8 periods	Gradient	Reference	Y	Complete	N	Rough	Y
14	Strong	Time delay	–	8 periods	Gradient	High	Y	70% fused	N	Very rough	Y
15	Strong	Time delay	–	4 periods	Constant	Low	Y	Complete	N	Smooth	N
16	Strong	Time delay	–	4 periods	Constant	Reference	Y	Complete	N	Rough	N
17	Strong	Time delay	–	4 periods	Constant	High	Y	60% fused	N	Very rough	N
18	Strong	FGF8 threshold	8.2 nM	1 somite length	Gradient	Low	Y	Complete	Some	Rough	Y
19	Strong	FGF8 threshold	8.2 nM	1 somite length	Gradient	Reference	Y	Good	Some	Rough	Y
20	Strong	FGF8 threshold	8.2 nM	1 somite length	Gradient	High	Y	60% fused	N	Very rough	Y
21	Strong	FGF8 threshold	2.1 nM	4 somite lengths	Gradient	Low	Y	Complete	Some	Rough	Y
22	Strong	FGF8 threshold	2.1 nM	4 somite lengths	Gradient	Reference	Y	Good	Some	Rough	Y
23	Strong	FGF8 threshold	2.1 nM	4 somite lengths	Gradient	High	Y	40% fused	N	Very rough	Y
24	Strong	FGF8 threshold	0.35 nM	8 somite lengths	Gradient	Low	Y	Complete	Some	Rough	Y
25	Strong	FGF8 threshold	0.35 nM	8 somite lengths	Gradient	Reference	Y	Good	Some	Rough	Y
26	Strong	FGF8 threshold	0.35 nM	8 somite lengths	Gradient	High	Y	60% fused	N	Very rough	Y
27	Strong	FGF8 threshold	2.1 nM	4 somite lengths	Gradient	Reference	N	Good	Some	Rough	Y

**Ref** indicates the reference simulation, chosen because it most closely resembles somitogenesis *in vivo*.

Adhesion strength: “Weak” refers to the adhesion values for the determined cell types closer to the simulated PSM; “Strong” refers to the adhesion values for the determined cell types as shown in **Table 3**.

Determination-differentiation mechanism: “Time delay” indicates that a cell-autonomous “ticker” was attached to each **cell** and counted down the interval between determination and differentiation; “FGF8 threshold” indicates that a second FGF8 threshold was set after determination, at which **cells** differentiated.

PSM cell motility: “Low” means λ_surf_ = 25 and D_cell_ = 0.86 µm^2^/min; “Reference” means λ_surf_ = 15 and D_cell_ = 1.08 µm^2^/min; and “High” means λ_surf_ = 5 and D_cell_ = 1.76 µm^2^/min.

Intersomitic gap formation: “Complete” means that every pair of somites in the simulation separated completely; “Good” means that somite boundaries are clear, but some **Core cells** persist in some intersomitic gaps; “% fused” gives the percentage by number of fused somites. See **Figure 10** for clarification of the difference between fused somites and persistence of stranded **Core cells**.

Border correction: “N” means that cell sorting does not correct cell mixing across presumptive intersomitic borders; “Y” means that cell sorting corrects any cell mixing across presumptive intersomitic borders; “Some” means that cell sorting corrects some, but not all borders after cell mixing occurs.

Compartment borders: “Smooth” means that the populations of determined cell types are clearly separated with a smooth border between them; “Rough” means that the populations of determined cell types are separated, but the border between them is rough, with some cell mixing; “Very rough” means that the populations of determined cell types were not clearly separated and the border between them is fragmented due to cell mixing.

Traveling Lfng stripes: “Y” means that apparent traveling stripes of Lfng expression were observed in the simulation; “N” means that the traveling stripes were not observed.

Somitogenesis in our simulations is sensitive to moderate (70%) changes in **cell** diffusion constants. Decreased **cell** motility in the **PSM** (*D*_cell_ = 0.86 µm^2^/min) hinders **somite** rounding (**Figure 9** (**C**), top of panel and **Figure S7** (**A–B**)) and prevents the variations in somite shape observed *in vivo* and in our reference simulations (see **Figure 7**, most data not shown). Increased **cell** motility (*D*_cell_ = 1.76 µm^2^/min) leads to over-mixing of **cells**, which can move significant distances in the AP direction, making adhesion-driven **cell** sorting insufficient to reform clean intersomitic boundaries and resulting in fused **somites** (**Figure 9** (**C**), bottom of panel and **Figure S7** (**C**)). Our reference simulations use an intermediate **cell** motility for **PSM cells** (*D*_cell_ = 1.08 µm^2^/min) comparable to the average cell motility measured in the PSM of chick embryos (*D*_cell_∼1.0 µm^2^/min) [25]. This degree of **cell** motility is high enough to allow **cell** sorting at the forming intersomitic borders, **somite** rounding and variation in **somite** shapes, yet not so high that **cell** motion prevents appropriate border determination and **somite** separation (**Figure 7**, **Figure 9** (**C**), middle of panel and **Figure S7** (**B**)). *In vivo* PSM cell motility in chick, as estimated from Figure 2 of [25], ranges between approximately 2.75 µm^2^/min in the extreme posterior PSM to approximately 0.25 µm^2^/min in the extreme anterior PSM, so the range of **cell** motilities used in our simulations is experimentally reasonable.

In experiments, cells involved in somite border formation transiently increase their motility [2]. This momentary increase also occurs in our simulations and arises spontaneously due to adhesion-driven **cell** sorting following differentiation. Measured diffusion constants for **cells** at the forming border can be as high as 3.0 µm^2^/min, as is the case for the **cell** marked with a red dot in **Figure 7** (**G**–**M**), while the diffusion constant is only a third of this for a typical **cell** in the same simulation (*D*_cell_ = 1.08 µm^2^/min for *λ*_surf_ = 15). Their higher motility results from directed migration of the misplaced **cells** to their final positions in response to changes in their local environments, *i.e.*, neighboring **cells**' adhesion properties.

### Somites form *in silico* in the absence of traveling gene expression stripes

Traveling stripes of gene expression are observed in the PSM of many species, including chick, mouse, zebrafish and corn snake [3], [10], [32], [77]. Gibb *et al.* [76] proposed that a Wnt-gradient-based segmentation-clock period gradient and the resulting traveling stripes of high protein concentration are conserved across species, and suggested that traveling stripes may play an important role in somitogenesis. *In vivo*, stripes arrest and stabilize at the position of the next presumptive somite, suggesting that they may be involved in cell-type specification and/or differentiation prior to somite formation. Such a mechanism would be a significant extension of a pure clock-and-wavefront model, which does not require a non-uniform segmentation-clock phase profile in the PSM. Indeed, the original clock-and-wavefront model [11] included intracellular segmentation clocks but neither required nor predicted anteriorly-traveling stripes of high protein concentration.

Simulations of our model with a uniform Wnt3a concentration corresponding to the level of the determination front in our reference simulation did not produce traveling stripes of high Lfng concentration (see **Figure 8** (**D**)) but formed normal **somites** (**Figure 9** (**D**) and **Figure S8**), suggesting that traveling stripes and AP variation in segmentation-clock period are not essential for somitogenesis. However, the stripes may play a role in aspects of somitogenesis or subsequent development that are not accounted for in our model.

### Somites form *in silico* for a wide range of delays between cell-fate determination and cell differentiation for determined cells with intermediate adhesion properties

We chose an interval of four segmentation-clock periods as the reference delay between **cell** fate determination and **cell** differentiation based on Dubrulle and colleagues' experimental observation that in chick cell-type determination occurs approximately four somite lengths in advance of the newest somite border [8]. The duration of the delay varies among organisms [8], [77], [80], but neither the need for a delay nor the reasons for its duration are apparent. For the clock-and-wavefront mechanism to function in multiple species, it must function over a wide range of delay times. We simulated **somite** formation for determination-to-differentiation delays ranging from zero to 8 segmentation-clock periods. Clean **somite** borders formed for all delays simulated (**Figure 9** (**E–F**) and **Figure S9**). To present a more challenging case, we ran simulations with long (eight segmentation-clock periods) determination-to-differentiation delays and determined **cell** types with adhesive properties closer to those of undetermined **PSM cells**. These simulations showed more mixing between **cells** with distinct determined **cell** types than in the reference simulation, but, after differentiation, the **cells** still sorted into distinct populations with well-defined borders, forming **somites** indistinguishable from those in the reference simulation (**Figure 9** (**G**) and **Figure S10**).

### Somite formation *in silico* is sensitive to long intervals between determination and adhesion-property changes

Together, the above results suggest that somitogenesis is relatively insensitive to the length of the determination-differentiation delay and may not require a determination-to-differentiation interval. One simplification included in our model, however, is that immediately after determination **cells** change their adhesion properties slightly, whereas *in vivo* determined cells take some time to express even low concentrations of adhesion molecules at their membranes. In order to more closely model the situation *in vivo*, we introduced an interval between **cell** fate specification and any changes to their adhesion properties.

In one set of simulations, **cells** underwent differentiation immediately after the interval, with no period of intermediate adhesion. For intervals of fewer than two segmentation-clock periods, these simulations formed **somites** that were joined by a greater-than-normal number of **Core cells** stranded in the intersomitic gaps (data not shown). For intervals of two or more segmentation-clock periods, these simulations formed **somites** that were predominantly fused or joined by large numbers of **Core cells** stranded in the intersomitic gap.The severity of the *in silico* phenotype increased with increasing intervals (**Figure 9** (**H–K**) and **Figure S11**).

In a second set of simulations, **cells** had **determined-cell-type** adhesions for the remainder of the standard four-segmentation-clock-period determination-differentiation delay. In these simulations, **cell** sorting during the period of intermediate adhesion partially corrected the *in silico* phenotype, preventing fused **somites** in simulations with intervals of two segmentation-clock periods, decreasing the number of fused **somites** in simulations with intervals of greater than two segmentation-clock periods, and decreasing both the number of stranded **Core cells** and the number of **somites** joined by stranded **Core cells** for all tested intervals (see **Figure 9** (**H–K**) and **Figure S11**, some data not shown).

Results from the previous section (see **Figure 9** (**E–F**) and **Figure S9**) indicate that the difference between the outcomes of these two sets of simulations are not due exclusively to the difference in the duration of the period of intermediate adhesion: in the absence of an interval between **cell**-fate specification and changes to **cells'** adhesion properties, **somites** form normally even for very short periods of intermediate adhesion (**Figure 9** (**E**) and **Figure S9 (A–C)**).

These results suggest that cell sorting during the determination-differentiation delay is functional and enables a required error correction mechanism depending on gradually changing adhesion properties, as approximated in our first set of simulations.

## Discussion

### Integration reveals previously unappreciated features and limitations of individual submodels

Our integrated model of somitogenesis combines submodels operating at different scales: an extended and corrected Goldbeter-Pourquié intracellular segmentation-clock network [16], Lewis-like Delta/Notch cell-cell segmentation-clock synchronization [5], signaling gradients produced by FGF8 mRNA and Wnt3a protein decay [9], [20], and a simplified version of Glazier *et al.*'s 2D model of differential cell adhesion- and motility-driven intersomitic gap formation and intrasomitic compartment maintenance [7], as well as novel models of cell-type determination and differentiation.

Integrating these submodels revealed previously unappreciated features and limitations of those submodels. While the Goldbeter-Pourquié intracellular segmentation-clock network and Lewis-style intercellular Delta/Notch coupling seem reasonable when considered separately, combining them showed a significant limitation of the Goldbeter-Pourquié model: even with the addition of intercellular coupling through the Delta/Notch loop, the model is unable to entrain Wnt and FGF oscillations among **cells**. We resolved this inconsistency in our extended submodel by adding experimentally-supported feedback from the Notch pathway to the FGF pathway [38] (additional juxtacrine signaling between **cells'** FGF and/or Wnt oscillators could also solve the problem but is experimentally unsupported, though not ruled out). Our extended segmentation-clock submodel is able to synchronize neighboring **cells** and maintain synchronization for realistic levels of **cell** motion and neighbor-exchange in the absence of significant additional perturbation, but is limited in its ability to synchronize neighboring **cells** when we introduce additional perturbations like a random initial distribution of clock phases (**somite** formation is robust for variations of about ±5% in initial clock phases, but fails for greater variation [data not shown]).

At the same time, the Goldbeter-Pourquié segmentation-clock model has the previously unreported feature that its oscillation period responds to the degree of Wnt signaling. This property is responsible for the spontaneous emergence in our model of “pseudo-waves” of Lfng expression that resemble the traveling stripe patterns observed *in vivo*. The extended clock submodel also produces a consistent phase relationship among oscillating components, allowing a plausible mechanism for translating internal **cell** states at determination into mechanical properties that distinguish differentiated **cell** types.

While we implemented a particular submodel for **cell** determination based on a set of provocative observations [8], the results of our integrated model do not depend in detail on this particular determination submodel. The network in **Figure 4** (**A**) is only a speculative mechanism connecting the segmentation clock, determination front and the mechanical properties that characterize subpopulations of cells. We currently lack the experimental evidence necessary to construct a realistic model network capable of explaining PSM cell determination. The results we present here do, however, demonstrate that fate determination according to a cell's internal state (*i.e.*, the phase of its segmentation clock) at the time that it encounters a determination front is a plausible mechanism for patterning a dynamic PSM.

In our submodel of the morphogen gradients, we neglect possible non-diffusion mechanisms of molecular transport such as endocytotic transport (discussed in [46], [81], [82], [83]). We also neglect potential feedback between FGF8 and Wnt3a signaling (aside from their interaction within the segmentation-clock network). Signaling in the integrated model is thus close to cell-autonomous for FGF8 and cell-autonomous for Wnt, so that the magnitude of signaling depends strongly on the amount of time that a **cell** has been in the **PSM** rather than on its AP position. Because time spent in the **PSM** and AP position correlate closely, the impact of this simplification is relatively slight, but not completely negligible, particularly in cases of high **cell** motility. Groups of **cells** that enter the simulation at approximately the same time determine and differentiate roughly simultaneously regardless of their spatial separation.

Non-**cell**-autonomous mechanisms play a significant role in producing the model's results. This is seen in simulations in which **cells** retain their **PSM**-like adhesion properties for some time after receiving their somitic fates. Allowing **cells** to mix for some time after receiving their fates, but before they change their adhesion properties, disrupts **somite** formation. Were **cells** acting completely autonomously, the cases with and without this post-determination mixing would be identical, since **cells** would have had determined fates from the moment they entered the simulation.

Our model strengthens the claim made in [7] that differential adhesion is capable of translating patterns of protein expression into tissue morphology in the PSM. While the previous work periodically imposed patterns of the relevant adhesion molecules, induced determination and differentiation in conjunction with **cell** motion results in a more stochastic pattern of adhesion molecules; nevertheless **cell** rearrangements due to differential adhesion still produce dynamic morphologies reminiscent of those *in vivo*.

Because our model does not include mechanisms for border correction beyond adhesion-mediated cell sorting, it is sensitive to over-mixing of **cell** types that can arise either as a consequence of initial mis-determination (which can be due to local segmentation-clock desynchronization or to significantly different levels of FGF8 among neighboring **cells** at the determination front) or from extensive **cell** diffusion in the interval between determination and differentiation. The impact of over-mixing is particularly pronounced in a 2D model, in which **cell** motion is constrained to a single plane. In 2D a **cell** is more likely to become surrounded on all sides by **cells** of an inappropriate type, trapping the mislocated **cell** in a local adhesion-energy minimum and impeding correction of the mistake by adhesion-driven sorting.

Experiments suggest that Eph-ephrin signaling, epithelialization and cell-ECM interactions all play significant roles in forming the intersomitic gap [22], [27], [84], [85]. Our current model omits detailed submodels of these mechanisms, relying solely on differential adhesion between EphA4-, ephrinB2- and N-cadherin-expressing **cells** to form and maintain the gap. These mechanisms likely operate in concert; for instance, localization of EphA4-expressing cells in the anterior somite compartment and anterior tip of the PSM and ephrinB2-expressing cells in the posterior somite compartment arises from mechanisms included in our model and facilitates the Eph-ephrin signaling across the presumptive intersomitic gap that is omitted from our model. The occasional failure of our model to produce perfectly separated **somites** in situations with increased noise due to high cell motility or perturbations in the segmentation clock is almost certainly due to the lack of these additional mechanisms. That our model does reproduce the major events in somitogenesis in a realistic fashion suggests that the mechanisms included in the model are the most significant ones in tissue patterning *in vivo*.

### Testable predictions of the integrated model and suggested future experiments

The emergent behaviors that arise within the integrated model and its response to certain perturbations lead to a series of experimentally testable predictions. In our model the spontaneous transient increase in **cell** motion during **somite** formation results from sorting due to differential **cell**-**cell** adhesion, predicting that experimentally interfering with the mechanics of cell adhesion and effective repulsion will decrease measured cell motility and hamper cell rearrangement along forming somite borders. Although experiments have shown that boundary formation in zebrafish requires Eph/ephrin signaling [22], [40], a detailed study of the effects of EphA4 and ephrinB2 knock-outs on cell-cell adhesion and boundary dynamics is still lacking, particularly in chicken and mouse.

The adhesion-driven cell-sorting submodel predicts the persistence of some misplaced cell types well into somitogenesis; so in experiments which label cells based on anterior and posterior somitic markers, a few misplaced cells (according to their genetic AP identities) should persist in the most recently-formed somites.

Simulating organisms with more cells per somite only required changing the period of the segmentation clock or the **PSM** growth rate. Simulating organisms with fewer cells per somite additionally required decreased **cell** motility due to the narrow width of the stripes of EphA4-/ephrinB2-expressing **cells**. This result suggests that smaller embryos (those with fewer cells per somite) require stricter control on spatial stochasticity, predicting that evolutionary pressures will lead to noisier (more error-prone) differentiation in organisms with big somites than in organisms with smaller somites. Since regulation of **cell** motility (as modeled in this work), the strength of the segmentation-clock synchronization mechanism and the rapidity of expression and accumulation of adhesion molecules at the membrane following **cell**-type determination all affect noise levels, we predict that measurement of these quantities in a variety of species will show stricter control in smaller embryos.

Many mathematical/computational models of somitogenesis have assumed that some external factor such as FGF8 or Wnt3a signaling modulates the period of the segmentation clock [37], [72], [76], [78], [79]. While we did not impose such an assumption, Lfng travelling stripes arose spontaneously in our simulations via an emergent version of this mechanism. Because the period-modulating Wnt3a signal in our model is **cell**-autonomous, the traveling stripes of Lfng expression in our simulations are nearly **cell**-autonomous pseudo-waves. Our model predicts, therefore, that excising segments of the PSM will not disrupt traveling Lfng stripes. Similarly, the model predicts that tissue inversion experiments should result in reversed-direction traveling stripes of mRNA/protein expression in the inverted tissue that closely resemble normal traveling stripes apart from their direction. The model also predicts that imposing uniform Wnt3a signaling across the PSM by knocking out endogenous Wnt3a signaling and applying a uniform concentration of Wnt3a to the tissue will eradicate travelling-stripe expression of segmentation-clock genes, but not their oscillations or the formation of normal somites (see **Figure 8** (**D**), **Figure 9** (**D**) and **Figure S8**).

Even if different mechanisms were responsible for the traveling stripes of gene expression in the PSM, as long as the mechanisms are cell-autonomous, *e.g.*, if the segmentation-clock oscillations gradually slow as a function of the cells residence time in the PSM (as occurs in our model due to the decay of Wnt3a) or if the stripes result from conserved clock phase differences between cells as they enter the posterior PSM, then our predictions for surgical experiments will not distinguish between such mechanisms and those in our model, but our predictions for the effect of Wnt3a manipulation would change. If the expression stripes are propagating waves arising from the physical boundary conditions and segmentation-clock coupling between cells, altering Wnt3a expression will not have the effect our model predicts, and surgically manipulating the PSM would disrupt the waves.

**Text S3** lists the major assumptions, simplifications and experimental features included in the integrated model, and the experimental features reproduced by the model.

### Future directions

The present work primarily tests the ability of an integrated model composed of existing submodels of somitogenesis at different scales to reproduce key dynamical and morphological features of *in vivo* somitogenesis. It does not explore a wide range of alternate mechanisms or submodels at each scale. The groundwork laid here and the modular nature of the integrated model will allow us to extend the model to perform more focused explorations of particular mechanisms important to somitogenesis.

We excluded a detailed submodel of RA signaling and RA-FGF8 interaction from the current integrated model, choosing instead to model the simplest-case scenario involving morphogen-segmentation-clock interaction and threshold-positioned developmental fronts. In addition to other functions, RA probably refines determination and/or differentiation front positioning. Mutually antagonistic RA and FGF8 signaling may lead to a “bistability window” in which cells can abruptly switch between states of high FGF8 and low RA signaling to states of high RA and low FGF8 signaling, possibly leading cohorts of cells to simultaneously undergo determination or differentiation [45],[79]. The next generation of our integrated model will include submodels for RA production and distribution similar in their levels of detail to those for FGF8 in the current integrated model, as well as submodels of the interactions between modeled morphogens, so that we may address such questions as how having groups of cells determine simultaneously affects the necessity of striped gene expression in the anterior PSM and whether RA increases the robustness of determination and differentiation positioning to perturbations due to cell motion and noise in the segmentation clock.

Other extensions of our current integrated model will include submodels of FGF signaling-dependent cell motility, as described by Delfini *et al.* [24]; gradual changes in cell adhesion after determination, which could change the reorganization dynamics of cells after differentiation; and the addition of somite epithelialization, which may improve the realism of simulated somite border dynamics and result in more realistic somite morphologies.

Finally, the most limiting assumption of the current integrated model is that a 2D AP-by-ML slice along the DV midline serves as a proxy for 3D somitogenesis. Extending the model into three dimensions will allow us to address the impact of dimensionality on our key results (*e.g.*, by assessing the relative sensitivity of 2D and 3D models dynamics to changes in **cell** motility and adhesion parameters, and the ability of segmentation clocks in neighboring **cells** to synchronize). A 3D model will also allow us to address DV asymmetries both in boundary conditions and the emergent structure of somites.

Multi-scale modeling of highly conserved developmental processes such as somitogenesis is a necessary first step in developing predictive models of how potential toxins or therapies affect development. Even if the interactions of a chemical agent are predictable on the molecular scale, we need multi-scale models to predict how these molecular perturbations will affect tissue-, organ- and organism-level dynamics and morphologies, *e.g.*, somitogenesis and later segmental development. Ultimately, understanding how perturbations at a single scale propagate to other scales will be essential to evaluating whether the perturbations are likely to have therapeutic or dangerous effects. While such powerful predictive tools are still some way in the future, they will only be possible with the aid of flexible, well-crafted, well-understood multi-scale models.

## Supporting Information

Figure S1 **Schematic: Lewis oscillator.** Lewis' biological pathway submodel for synchronization of negative-feedback Her oscillators in two adjacent cells coupled through juxtacrine Delta signaling (after [5]). For more information see **INTRODUCTION** **: Model of the delta/notch segmentation-clock synchronization**.
(TIF)

Figure S2 **Typical FGF8 evolution of morphogen gradients in simulated PSM.** (**A**) *fgf8* mRNA concentration along the A–P centerline of the simulated PSM at 0, 180, 360, 540 and 720 min. (**B**) FGF8 concentrations at the same times. The color scale is the same as in **Figure 5** (red corresponds to 45 nM and blue to 0 nM). Anterior to left. Direction of **PSM** growth to right (posterior). Scale bar 40 µm. Parameter values: *D*_FGF8_ = 0.6 µm^2^/min; *k*_FGF8_ = 0.2 min^−1^; *mfgf*_0_ = 5.0 nM; *k*_mfgf_ = 0.005 min^−1^; *s*_fgf_ = 1.83 min^−1^; *C*_f2w_ = 0.32; PSM growth rate = 1.63 µm/min. For more information see **METHODS** **: Morphogen gradients**.
(TIF)

Figure S3 **Simulated segmentation-clock behavior.** (**A**) Normalized Lfng, Axin2 and Dusp6 concentrations in a single **cell** for the network shown in **Figure 3**. The **cell** is self-coupled, *i.e.*, its incoming Delta signal is set equal to its outgoing Delta signal, to reproduce the behavior of a **cell** in a neighborhood of **cells** of the same segmentation-clock phase. (**B**) Lfng concentration in nine coupled **cells** with the on-diagonal **cells** initially displaced in phase by 40%. After two segmentation-clock periods, the oscillations phase-lock (the time-averaged standard deviation over each subsequent cycle is less than 6% of the average of the amplitudes after the first two periods). Parameter values used are listed in **Table S1**. For more information see **METHODS** **: Segmentation clock**.
(TIF)

Figure S4 **Effect of PSM growth rate on the Wnt3a profile in the simulated PSM.** Faster (slower) **PSM** growth lengthens (shortens) the **PSM**, leaving the anterior and posterior concentrations of Wnt3a unchanged (inset). When we normalize the AP position by the total **PSM** length, the Wnt3a profiles for different growth rates are nearly identical. The AP position of the anterior of the **PSM** is defined to be zero. **PSM** growth rates: (black line) 0.82 µm/min; (blue line) 1.63 µm/min; (red line) 3.27 µm/min. All other parameters are equal to those in the reference simulation (**Figure 7**). For more information see **RESULTS** **: The segmentation-clock period and PSM growth rate regulate somite size** and **The number of high Lfng concentration stripes in the simulated PSM depends on the segmentation-clock period, PSM growth rate and PSM length**.
(TIF)

Figure S5 **Somite size versus segmentation-clock period.** Decreasing the period of the segmentation clock to 67.5 min shrinks **somites** (**A**) compared to the reference (chick) simulations with a segmentation-clock period of 90 min (**B**). Increasing the period of the segmentation clock to 135 minutes (**C**) or 180 min (**D**) forms proportionally larger **somites**. Well-formed smaller **somites** require decreased **cell** motility (*λ*_surf_ = 20; *D*_cell_ = 0.945 µm^2^/min for **PSM cells** in (**A**)); larger **somites** form using the reference motility parameters (*λ*_surf_ = 15; *D*_cell_ = 1.08 µm^2^/min for **PSM cells** in (**B**–**D**)). In each case, we adjust the ML dimension to produce roughly circular **somites**. Segmentation and **somite** separation, however, succeed both for smaller and larger ML widths (data not shown). We increase or decrease the segmentation-clock frequency by varying how long we integrate the segmentation-clock ODEs during each time step; by doing so, we easily vary the clock frequency relative to other processes in the simulation without altering parameters within the segmentation-clock submodel or changing the clock response to FGF8, Wnt3a or Delta/Notch signaling. Scale bar 40 µm. All other parameters are equal to those in the reference simulation (**Figure 7**). **Cell** colors are the same as in **Figure 5**. Due to non-biological initial conditions, the first **somite** is always defective. For more information see **RESULTS** **: The segmentation-clock period and PSM growth rate regulate somite size**.
(TIF)

Figure S6 **Somite size versus the rate of PSM growth.** (**A**) Decreasing the rate of **PSM** growth to 1.08 µm/min compared to the reference simulation growth rate of 1.63 µm/min shrinks **somites**. (**B**) Reference simulation. (**C**,**D**) Increasing the rate of **PSM** growth to 2.04 µm/min (**C**) or 2.72 µm/min (**D**) forms proportionally larger **somites**. Well formed smaller **somites** require decreased **cell** motility (*λ*_surf_ = 25 in (**A**)); larger **somites** form using the reference **cell** motility parameters (*λ*_surf_ = 15 for (**B**–**D**)). In each case, we adjust the ML dimension to produce roughly circular **somites**. Segmentation and **somite** separation, however, both succeed for smaller and larger ML widths (data not shown). Scale bar 40 µm. All other parameters are equal to those in the reference simulation (**Figure 7**). **Cell** colors are the same as in **Figure 5**. Due to non-biological initial conditions, the first **somite** is always defective. For more information see **RESULTS** **: The segmentation-clock period and PSM growth rate regulate somite size**.
(TIF)

Figure S7 **Somite quality dependence on cell motility** ***in silico*** **.** We regulate **cell** motility by adjusting *λ*_surf_. (**A**) Low **PSM cell** motility (*λ*_surf_ = 25, *D*_cell_ = 0.86 µm^2^/min): **somite** borders form, **somites** round up slowly compared to the reference simulation, and **somite** shape varies less than in reference simulation. (**B**) Reference simulation, moderate **PSM cell** motility (*λ*_surf_ = 15, *D*_cell_ = 1.08 µm^2^/min): **cell** sorting corrects small amounts of initial **cell** mixing across presumptive **somite** borders, **somites** round up within a short time (about one segmentation-clock period after formation) and **somite** shape is variable. (**C**) High **PSM cell** motility compared to the reference simulation (*λ*_surf_ = 5, *D*_cell_ = 1.76 µm^2^/min): excessive mixing of **cell** types across presumptive **somite** borders leads to fused **somites**. Parameters, when not otherwise noted, are equal to those in the reference simulation (**Figure 7**). Anterior at the top. **Cell** colors are the same as in **Figure 5**. Due to non-biological initial conditions, the first **somite** is always defective. Scale bar 40 µm. For more information see **RESULTS** **: Cell motility affects somite border formation and morphology**.
(TIF)

Figure S8 ***In silico*** **somitogenesis with a uniform Wnt3a concentration.** When [Wnt3a] is uniform throughout the **PSM**, traveling Lfng stripes do not form, but segmentation is normal, demonstrating that traveling stripes of high protein concentration are not necessary for somitogenesis in our model. The constant Wnt3a concentration actually improves synchronization between segmentation clocks in adjacent **cells**, reducing anterior-posterior cell misdifferentiation and increasing **somite** accuracy and regularity. Times: (**A**) 0 min, (**B**) 90 min, (**C**) 360 min, (**D**) 720 min, (**E**) 900 min, (**F**) 1080 min and (**G**) 1440 min. [Wnt3a] = 0.5 nM. All other parameters are equal to those in the reference simulation (**Figure 7**). Anterior at the top. Scale bar 40 µm. **Cell** colors are the same as in **Figure 5**. Due to non-biological initial conditions, the first **somite** is always defective. For more information see **RESULTS** **: Somites form** ***in silico*** **in the absence of travelling gene expression stripes**.
(TIF)

Figure S9 ***In silico*** **somite formation dependence on the time interval between determination and differentiation.** Somites form independently of the determination-differentiation delay for reference adhesion values. (**A**–**C**) Snapshots for a shorter than normal determination-differentiation delay of one segmentation-clock period (90 min) taken at (**A**) 450 min, (**B**) 750 min and (**C**) 1050 min. (**D**–**G**) Snapshots for a longer than usual determination-differentiation delay of eight segmentation-clock periods (720 min) taken at (**D**) 750 min, (**E**) 1050 min, (**F**) 1350 min and (**G**) 1860 min. The determination-differentiation delay in the reference simulation is four clock periods (360 min). Parameters, when not otherwise noted, are equal to those in the reference simulation (**Figure 7**). Anterior at top. Scale bar 40 µm. **Cell** colors are the same as in **Figure 5**. Due to non-biological initial conditions, the first **somite** is always defective. For more information see **RESULTS** **: Somites form** ***in silico*** **for a wide range of delays between cell-fate determination and cell differentiation for determined cells with intermediate adhesion properties**.
(TIF)

Figure S10 **Somite formation with increased post-determination cell mixing.** Assigning larger determination-differentiation delay (8 cell cycles) and determined **cell** types adhesion parameters closer to those of **PSM cells** than in the reference simulation increases **cell** mixing among distinct determined **cell** types prior to differentiation. However, **cell** sorting after differentiation corrects their moderate amount of mixing across presumptive **somite** borders and leads to clean **somite** boundaries. Times: (**A**) 750 min, (**B**) 1050 min, (**C**) 1350 min and (**D**) 1860 min. Anterior at top. Scale bar 40 µm. Contact energies: *J_pre_EphA4,pre_EphA4_* = −22; *J_pre_ephrinB2,pre_ephrinB2_* = −22; *J_pre_Core,pre_Core_* = −25; *J_pre_EphA4,EphA4_* = −22; *J_pre_ephrinB2,ephrinB2_* = −22; other contact energies are unchanged from **Table 3**. Parameters, when not otherwise noted, are equal to those in the reference simulation (**Figure 7**). **Cell** colors are the same as in **Figure 5**. Due to non-biological initial conditions, the first **somite** is always defective. For more information see **RESULTS** **: Somites form** ***in silico*** **for a wide range of delays between cell-fate determination and cell differentiation for determined cells with intermediate adhesion properties**.
(TIF)

Figure S11 **Effect of intermediate adhesion levels between determination and differentiation on segregation quality.** For an interval of two or more segmentation-clock periods between **cell** determination and any changes in adhesion and in the absence of a period of intermediate adhesion, the excessive mixing of determined **cell** types across their original borders leads to fused **somites** and a greater-than-normal occurrence of stranded **Core cells** in the intersomitic gaps: (**A–B**) two-clock-period interval after (**A**) 1035 min and (**B**) 1755 min; (**E–F**) two-and-a-half-clock-period interval after (**E**) 1035 min and (**F**) 1755 min. When **cells** have **determined-cell-type** adhesions for the remainder of the standard four-segmentation-clock-period determination-differentiation delay, **cell** over-mixing is partially corrected, preventing formation of fused **somites** and decreasing the occurrence of stranded **Core cells** for an interval of two segmentation-clock periods (**C–D**), and decreasing the frequency of fused **somites** and stranded **Core cells** for longer intervals (**G–H**): (**C–D**) two-clock-period interval followed by intermediate adhesion after (**C**) 1035 min and (**D**) 1755 min; (**G–H**) two-and-a-half-clock-period interval followed by intermediate adhesion after (**G**) 1035 min and (**H**) 1755 min. Except where otherwise stated, parameters are equal to those in the reference simulation (**Figure 7**). **Cell** colors are the same as in **Figure 5**. For more information see **RESULTS** **: Somite formation** ***in silico*** **is sensitive to long intervals between determination and adhesion-property changes**.
(TIF)

Table S1 **Model segmentation clock network parameters and initial conditions.**
(PDF)

Text S1 **Model segmentation clock network equations.**
(PDF)

Text S2 **Instructions for downloading and running simulation source code.**
(PDF)

Text S3 **Assumptions and features of the integrated model.**
(PDF)

Video S1 **Reference simulation visualizing cell types.**
(WMV)

Video S2 **Reference simulation visualizing extracellular FGF8 concentration.**
(WMV)

Video S3 **Reference simulation visualizing intracellular Lfng concentration.**
(WMV)

Video S4 **Reference simulation of long PSM visualizing cell types.**
(WMV)

Video S5 **Simulation of long PSM without intercellular Delta/Notch signaling visualizing intracellular Lfng.**
(WMV)
