# Supplementary figures and images for: A Multi-cell, Multi-scale Model of Vertebrate Segmentation and Somite Formation
Source: PLoS Comput Biol. 2011 Oct 6;7(10):e1002155. doi: 10.1371/journal.pcbi.1002155 (PMC3188485; doi:10.1371/journal.pcbi.1002155)

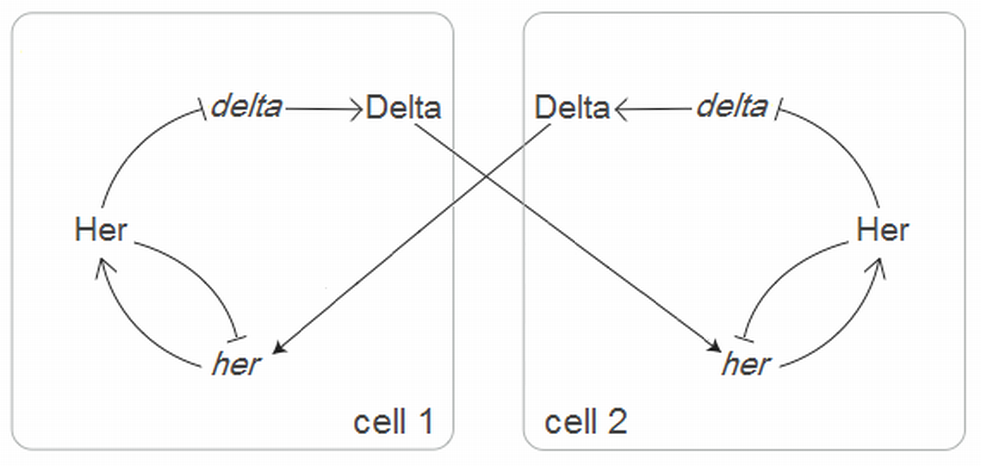

Supplement: Figure S1 — Schematic: Lewis oscillator. Lewis' biological pathway submodel for synchronization of negative-feedback Her oscillators in two adjacent cells coupled through juxtacrine Delta signaling (after [5]). For more information see INTRODUCTION : Model of the delta/notch segmentation-clock synchronization. (TIF) [file pcbi.1002155.s001.tif]

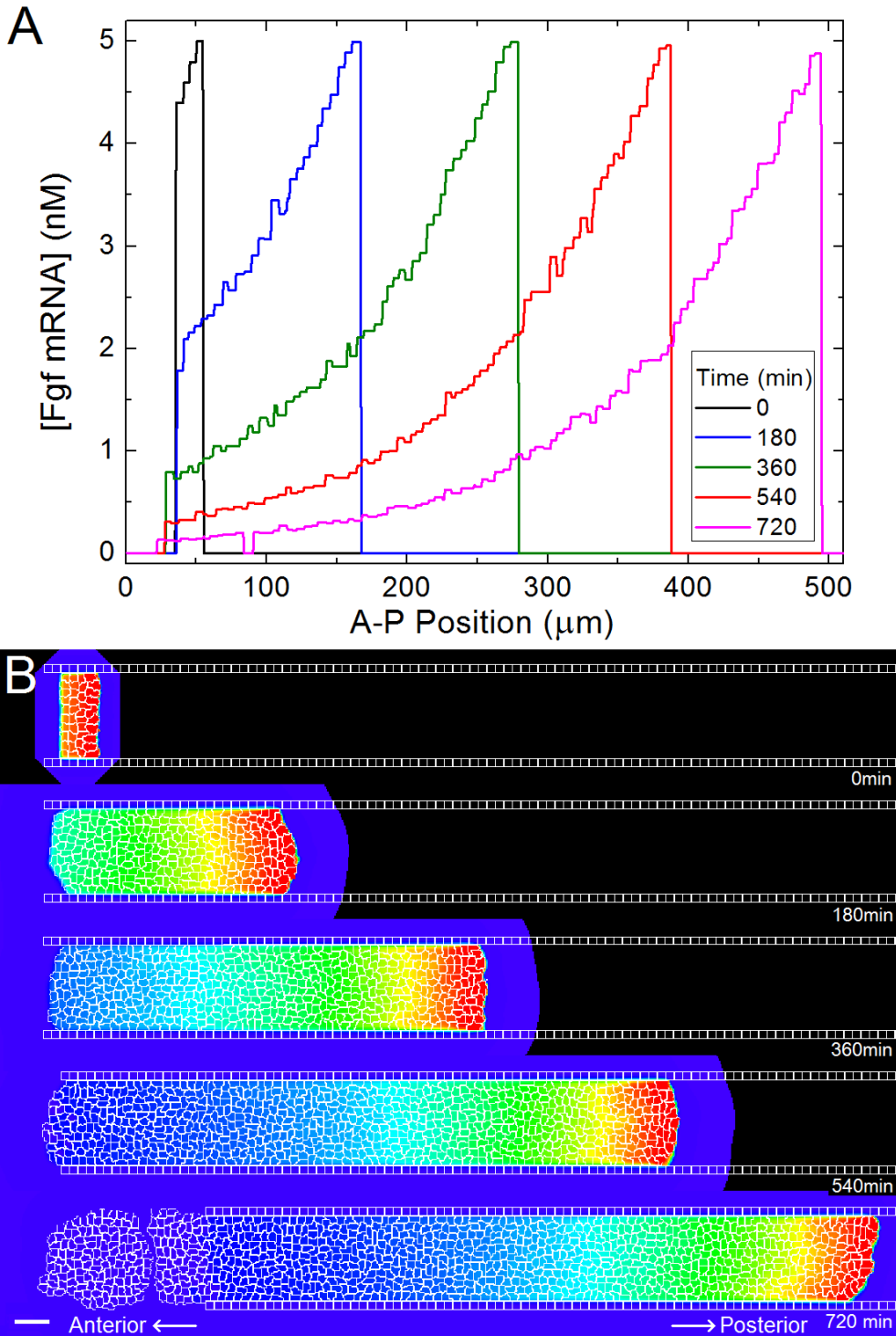

Supplement: Figure S2 — Typical FGF8 evolution of morphogen gradients in simulated PSM. (A) fgf8 mRNA concentration along the A–P centerline of the simulated PSM at 0, 180, 360, 540 and 720 min. (B) FGF8 concentrations at the same times. The color scale is the same as in Figure 5 (red corresponds to 45 nM and blue to 0 nM). Anterior to left. Direction of PSM growth to right (posterior). Scale bar 40 µm. Parameter values: D FGF8 = 0.6 µm2/min; k FGF8 = 0.2 min−1; mfgf 0 = 5.0 nM; k mfgf = 0.005 min−1; s fgf = 1.83 min−1; C f2w = 0.32; PSM growth rate = 1.63 µm/min. For more information see METHODS : Morphogen gradients. (TIF) [file pcbi.1002155.s002.tif]

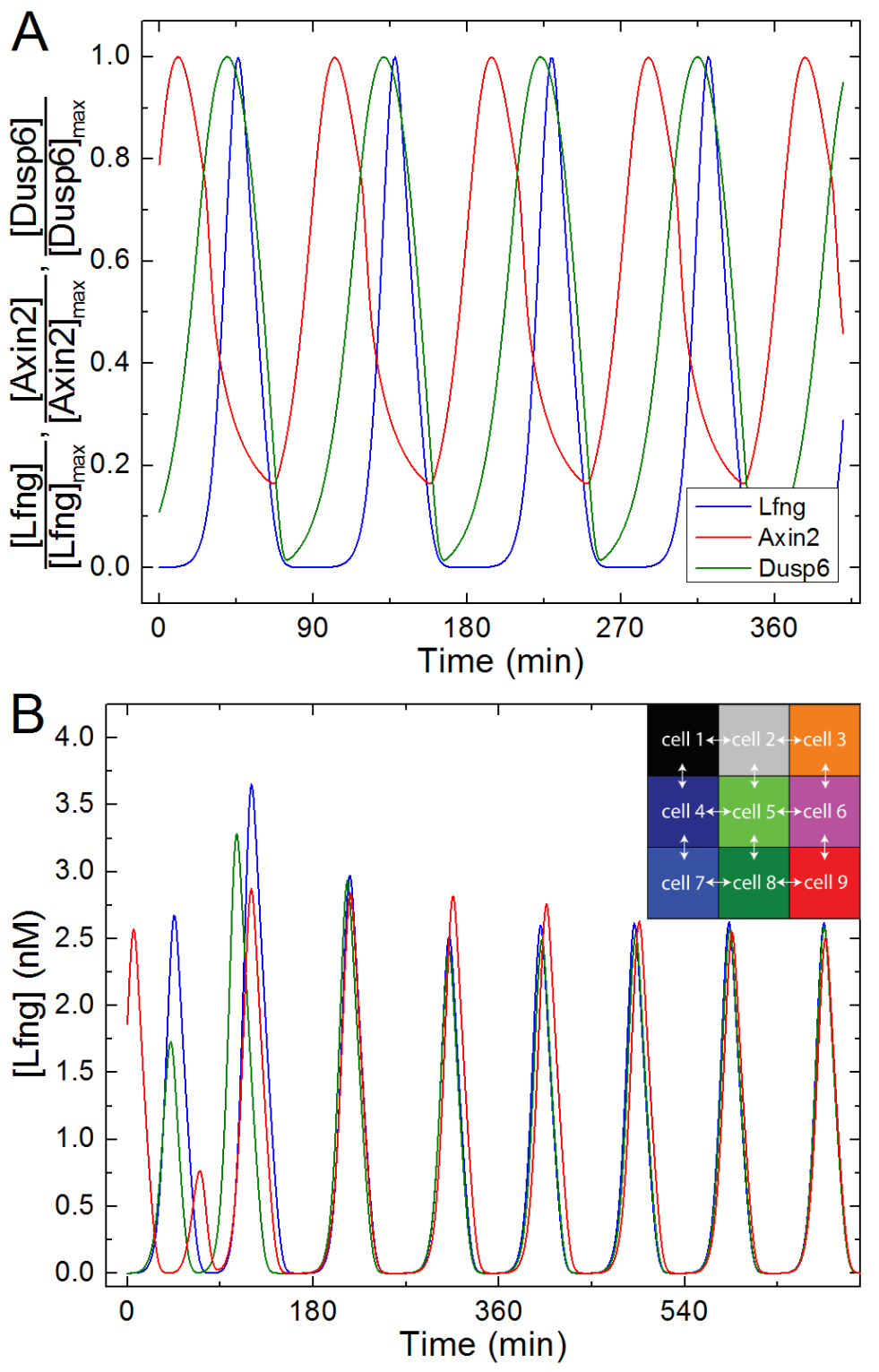

Supplement: Figure S3 — Simulated segmentation-clock behavior. (A) Normalized Lfng, Axin2 and Dusp6 concentrations in a single cell for the network shown in Figure 3 . The cell is self-coupled, i.e., its incoming Delta signal is set equal to its outgoing Delta signal, to reproduce the behavior of a cell in a neighborhood of cells of the same segmentation-clock phase. (B) Lfng concentration in nine coupled cells with the on-diagonal cells initially displaced in phase by 40%. After two segmentation-clock periods, the oscillations phase-lock (the time-averaged standard deviation over each subsequent cycle is less than 6% of the average of the amplitudes after the first two periods). Parameter values used are listed in Table S1. For more information see METHODS : Segmentation clock. (TIF) [file pcbi.1002155.s003.tif]

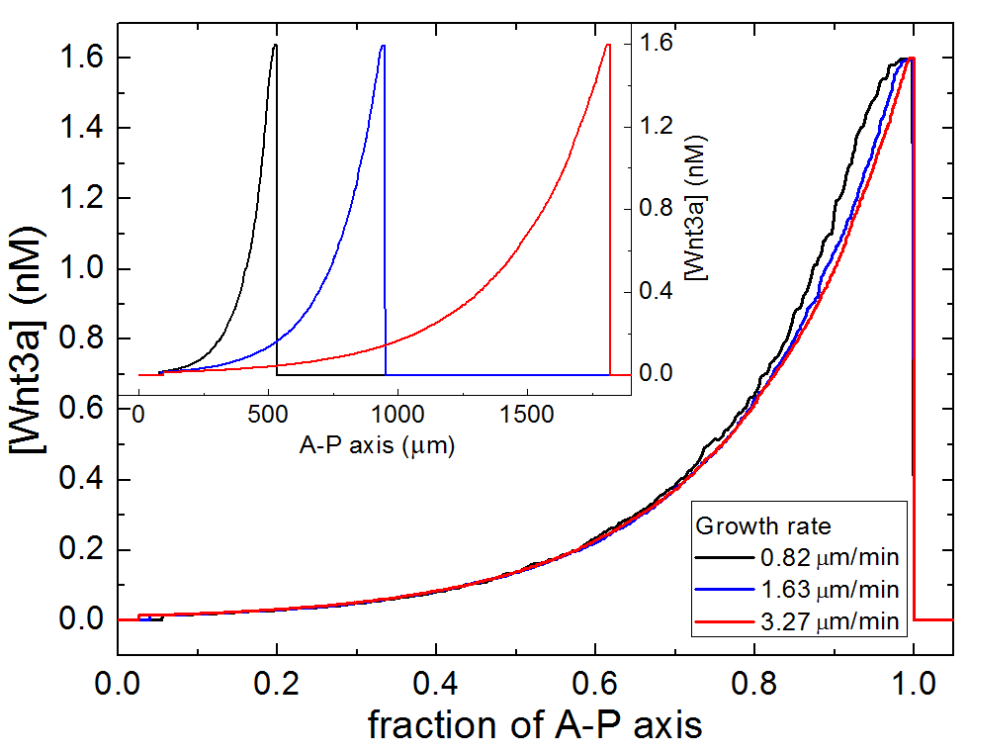

Supplement: Figure S4 — Effect of PSM growth rate on the Wnt3a profile in the simulated PSM. Faster (slower) PSM growth lengthens (shortens) the PSM, leaving the anterior and posterior concentrations of Wnt3a unchanged (inset). When we normalize the AP position by the total PSM length, the Wnt3a profiles for different growth rates are nearly identical. The AP position of the anterior of the PSM is defined to be zero. PSM growth rates: (black line) 0.82 µm/min; (blue line) 1.63 µm/min; (red line) 3.27 µm/min. All other parameters are equal to those in the reference simulation ( Figure 7 ). For more information see RESULTS : The segmentation-clock period and PSM growth rate regulate somite size and The number of high Lfng concentration stripes in the simulated PSM depends on the segmentation-clock period, PSM growth rate and PSM length. (TIF) [file pcbi.1002155.s004.tif]

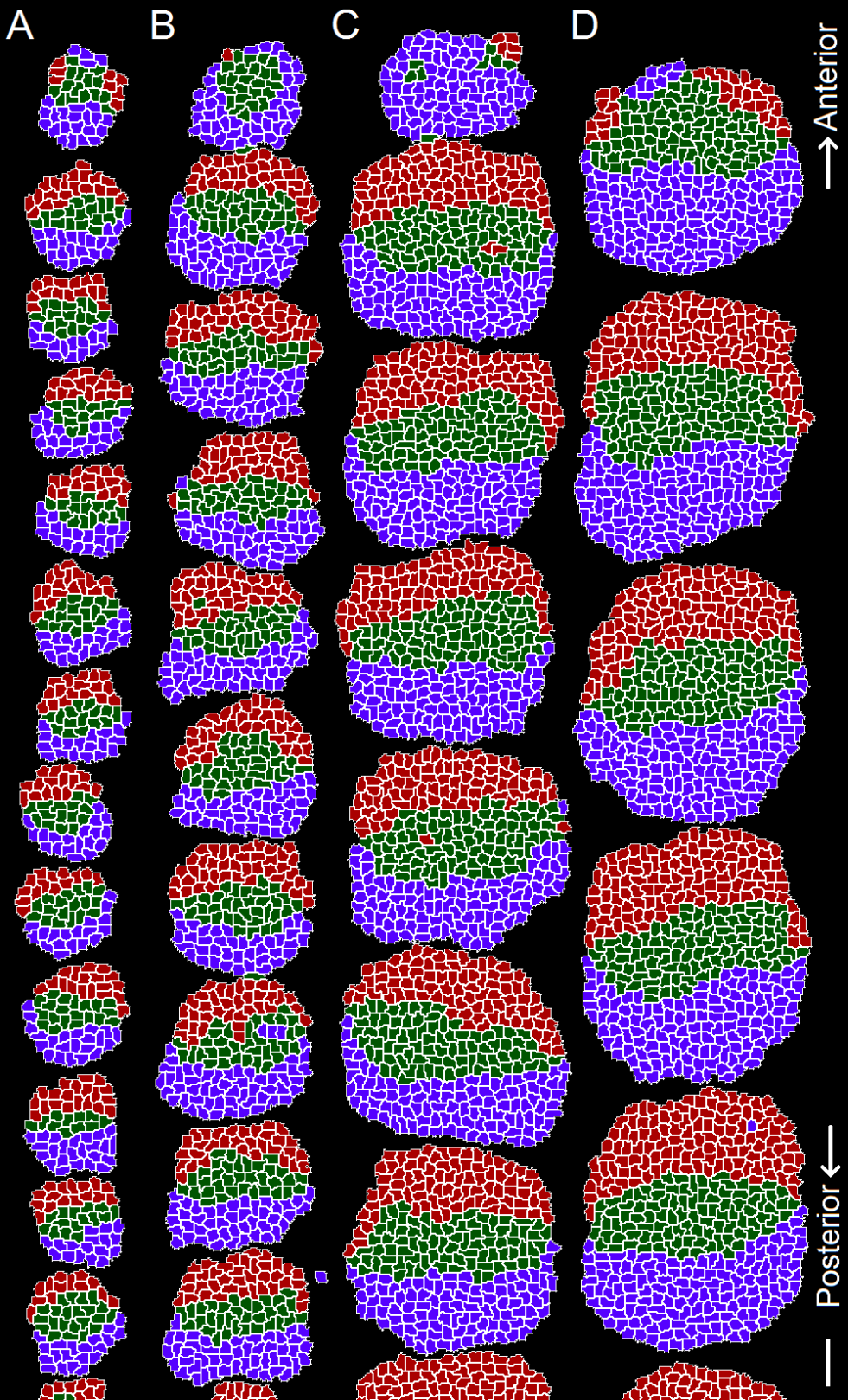

Supplement: Figure S5 — Somite size versus segmentation-clock period. Decreasing the period of the segmentation clock to 67.5 min shrinks somites (A) compared to the reference (chick) simulations with a segmentation-clock period of 90 min (B). Increasing the period of the segmentation clock to 135 minutes (C) or 180 min (D) forms proportionally larger somites. Well-formed smaller somites require decreased cell motility (λ surf = 20; D cell = 0.945 µm2/min for PSM cells in (A)); larger somites form using the reference motility parameters (λ surf = 15; D cell = 1.08 µm2/min for PSM cells in (B–D)). In each case, we adjust the ML dimension to produce roughly circular somites. Segmentation and somite separation, however, succeed both for smaller and larger ML widths (data not shown). We increase or decrease the segmentation-clock frequency by varying how long we integrate the segmentation-clock ODEs during each time step; by doing so, we easily vary the clock frequency relative to other processes in the simulation without altering parameters within the segmentation-clock submodel or changing the clock response to FGF8, Wnt3a or Delta/Notch signaling. Scale bar 40 µm. All other parameters are equal to those in the reference simulation ( Figure 7 ). Cell colors are the same as in Figure 5 . Due to non-biological initial conditions, the first somite is always defective. For more information see RESULTS : The segmentation-clock period and PSM growth rate regulate somite size. (TIF) [file pcbi.1002155.s005.tif]

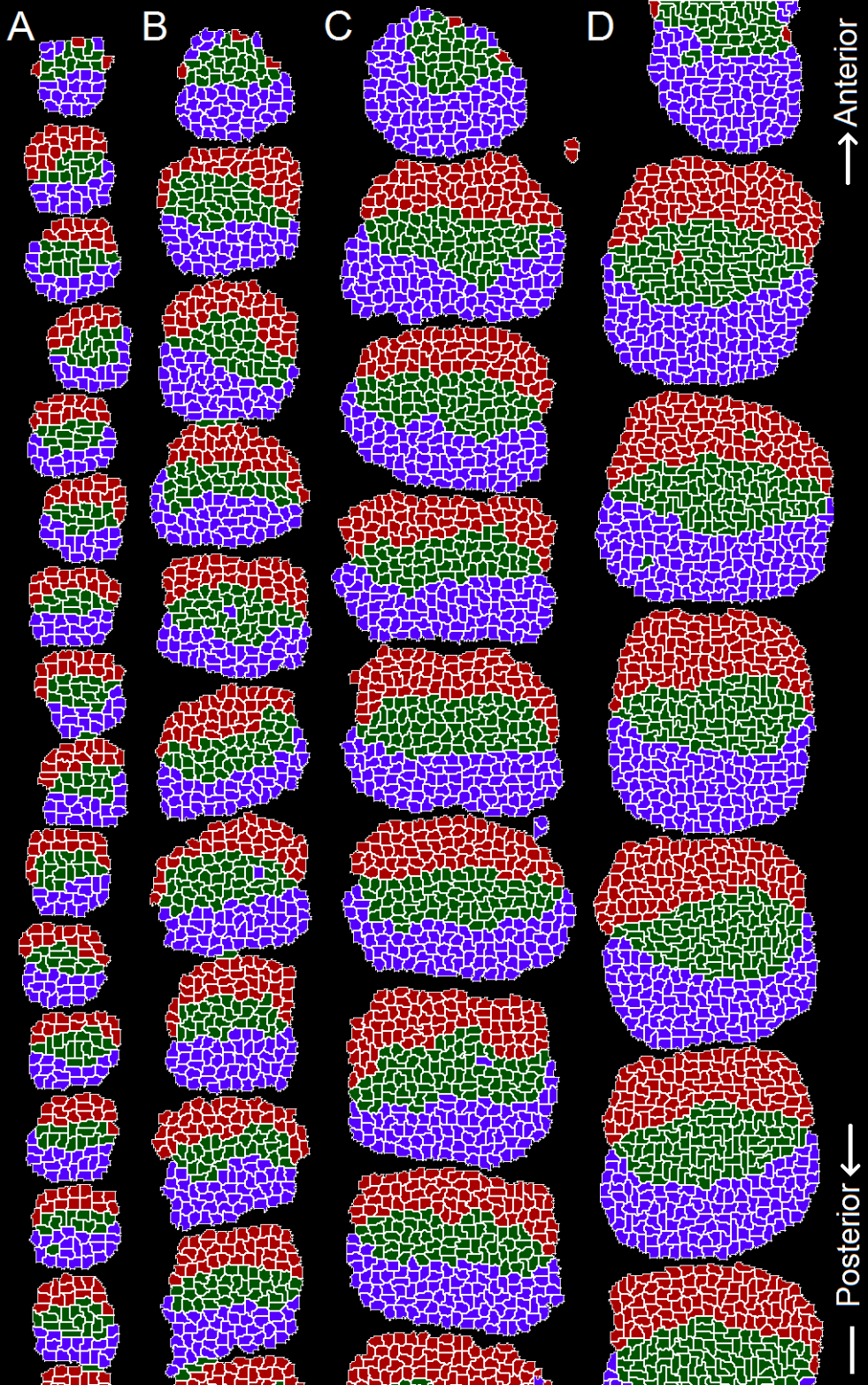

Supplement: Figure S6 — Somite size versus the rate of PSM growth. (A) Decreasing the rate of PSM growth to 1.08 µm/min compared to the reference simulation growth rate of 1.63 µm/min shrinks somites. (B) Reference simulation. (C,D) Increasing the rate of PSM growth to 2.04 µm/min (C) or 2.72 µm/min (D) forms proportionally larger somites. Well formed smaller somites require decreased cell motility (λ surf = 25 in (A)); larger somites form using the reference cell motility parameters (λ surf = 15 for (B–D)). In each case, we adjust the ML dimension to produce roughly circular somites. Segmentation and somite separation, however, both succeed for smaller and larger ML widths (data not shown). Scale bar 40 µm. All other parameters are equal to those in the reference simulation ( Figure 7 ). Cell colors are the same as in Figure 5 . Due to non-biological initial conditions, the first somite is always defective. For more information see RESULTS : The segmentation-clock period and PSM growth rate regulate somite size. (TIF) [file pcbi.1002155.s006.tif]

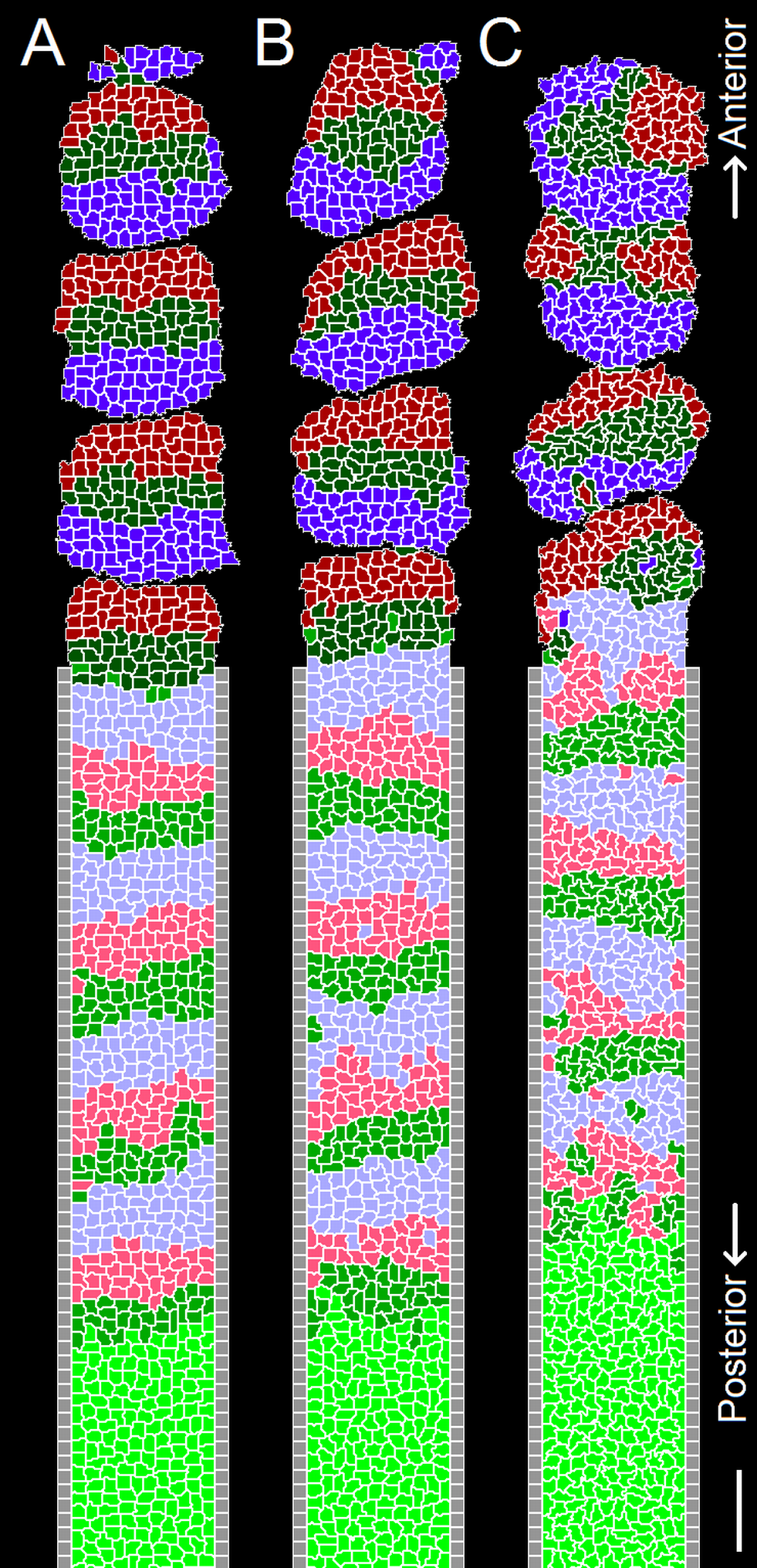

Supplement: Figure S7 — Somite quality dependence on cell motility in silico . We regulate cell motility by adjusting λ surf. (A) Low PSM cell motility (λ surf = 25, D cell = 0.86 µm2/min): somite borders form, somites round up slowly compared to the reference simulation, and somite shape varies less than in reference simulation. (B) Reference simulation, moderate PSM cell motility (λ surf = 15, D cell = 1.08 µm2/min): cell sorting corrects small amounts of initial cell mixing across presumptive somite borders, somites round up within a short time (about one segmentation-clock period after formation) and somite shape is variable. (C) High PSM cell motility compared to the reference simulation (λ surf = 5, D cell = 1.76 µm2/min): excessive mixing of cell types across presumptive somite borders leads to fused somites. Parameters, when not otherwise noted, are equal to those in the reference simulation ( Figure 7 ). Anterior at the top. Cell colors are the same as in Figure 5 . Due to non-biological initial conditions, the first somite is always defective. Scale bar 40 µm. For more information see RESULTS : Cell motility affects somite border formation and morphology. (TIF) [file pcbi.1002155.s007.tif]

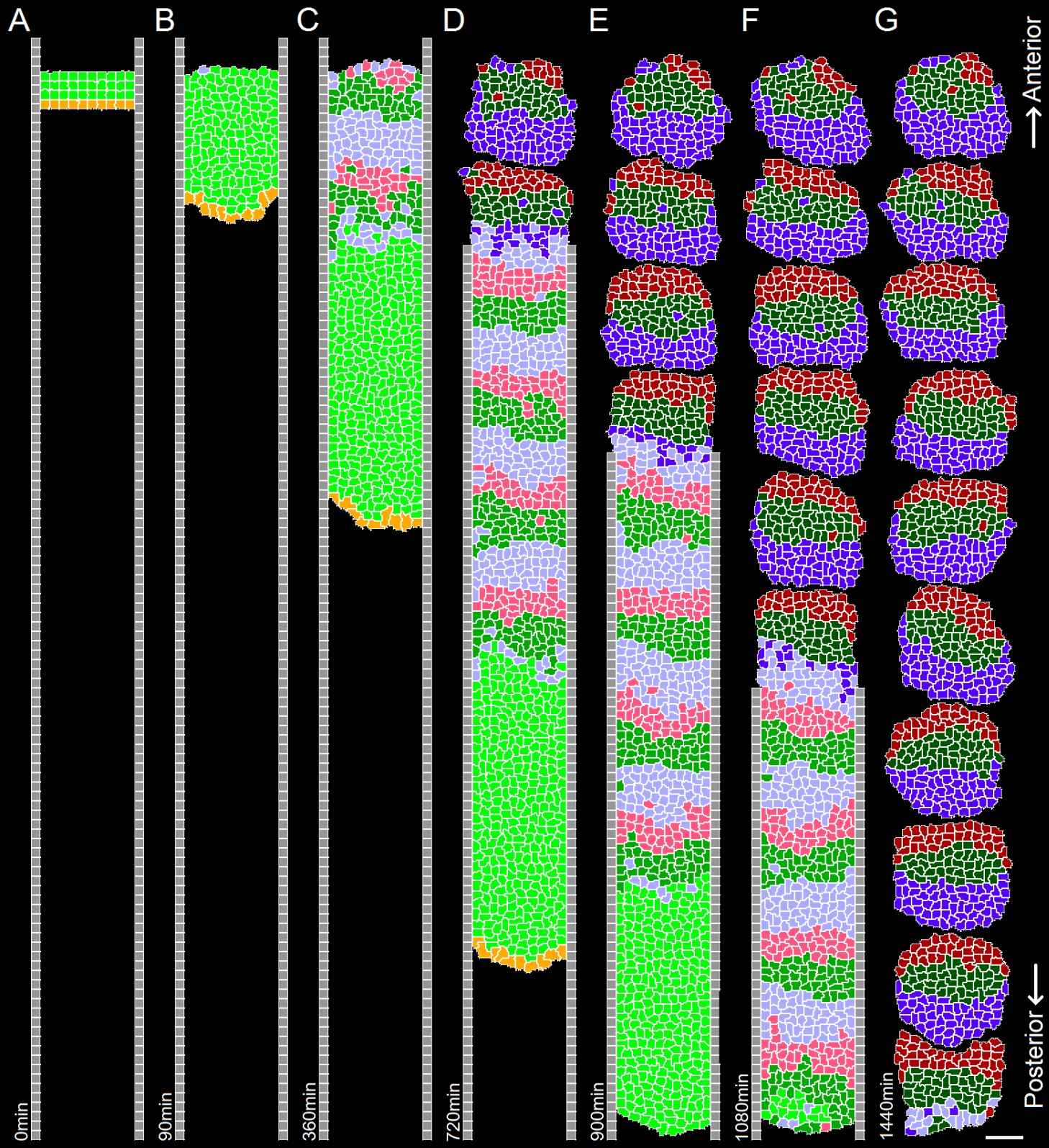

Supplement: Figure S8 — In silico somitogenesis with a uniform Wnt3a concentration. When [Wnt3a] is uniform throughout the PSM, traveling Lfng stripes do not form, but segmentation is normal, demonstrating that traveling stripes of high protein concentration are not necessary for somitogenesis in our model. The constant Wnt3a concentration actually improves synchronization between segmentation clocks in adjacent cells, reducing anterior-posterior cell misdifferentiation and increasing somite accuracy and regularity. Times: (A) 0 min, (B) 90 min, (C) 360 min, (D) 720 min, (E) 900 min, (F) 1080 min and (G) 1440 min. [Wnt3a] = 0.5 nM. All other parameters are equal to those in the reference simulation ( Figure 7 ). Anterior at the top. Scale bar 40 µm. Cell colors are the same as in Figure 5 . Due to non-biological initial conditions, the first somite is always defective. For more information see RESULTS : Somites form in silico in the absence of travelling gene expression stripes. (TIF) [file pcbi.1002155.s008.tif]

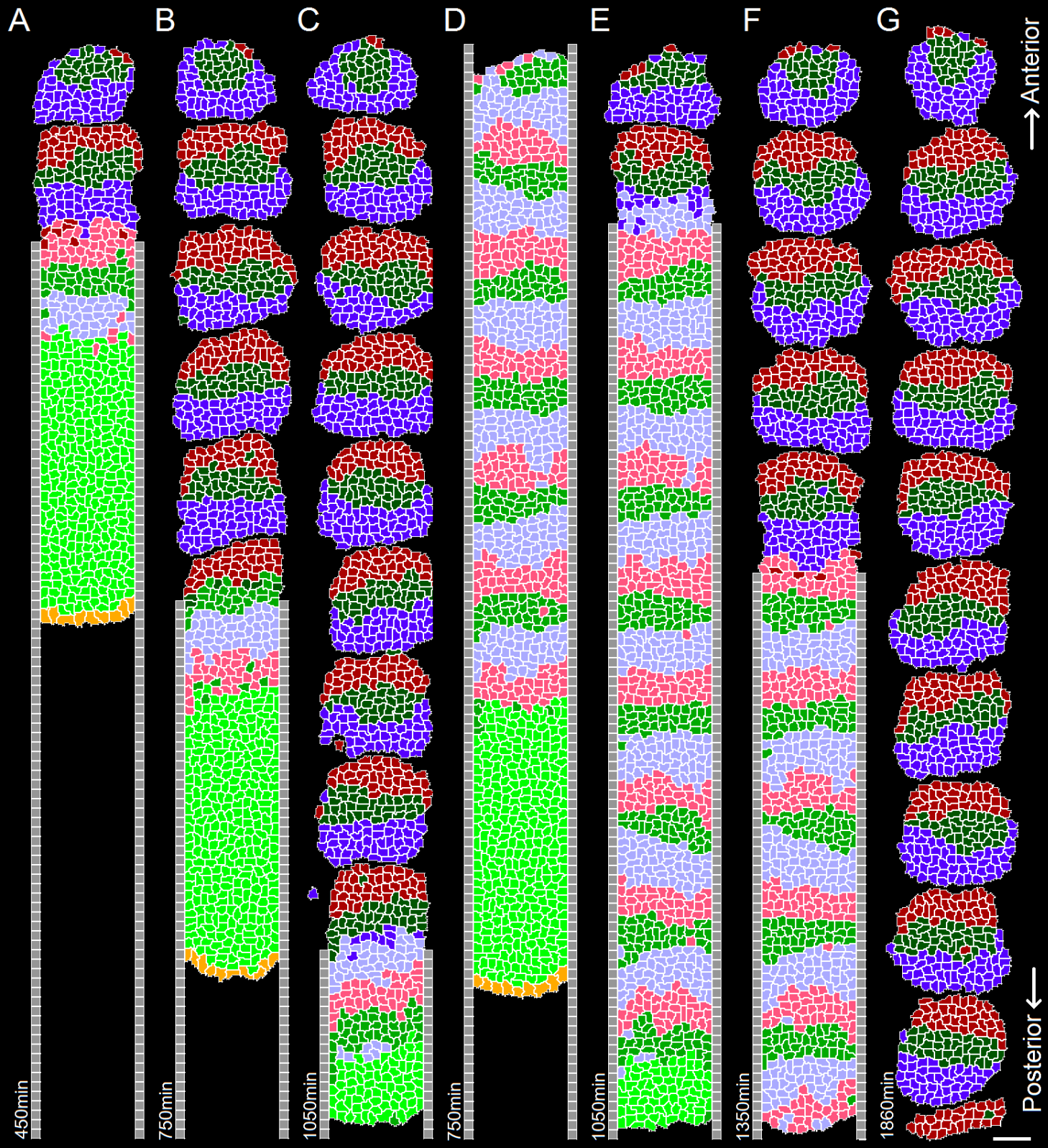

Supplement: Figure S9 — In silico somite formation dependence on the time interval between determination and differentiation. Somites form independently of the determination-differentiation delay for reference adhesion values. (A–C) Snapshots for a shorter than normal determination-differentiation delay of one segmentation-clock period (90 min) taken at (A) 450 min, (B) 750 min and (C) 1050 min. (D–G) Snapshots for a longer than usual determination-differentiation delay of eight segmentation-clock periods (720 min) taken at (D) 750 min, (E) 1050 min, (F) 1350 min and (G) 1860 min. The determination-differentiation delay in the reference simulation is four clock periods (360 min). Parameters, when not otherwise noted, are equal to those in the reference simulation ( Figure 7 ). Anterior at top. Scale bar 40 µm. Cell colors are the same as in Figure 5 . Due to non-biological initial conditions, the first somite is always defective. For more information see RESULTS : Somites form in silico for a wide range of delays between cell-fate determination and cell differentiation for determined cells with intermediate adhesion properties. (TIF) [file pcbi.1002155.s009.tif]

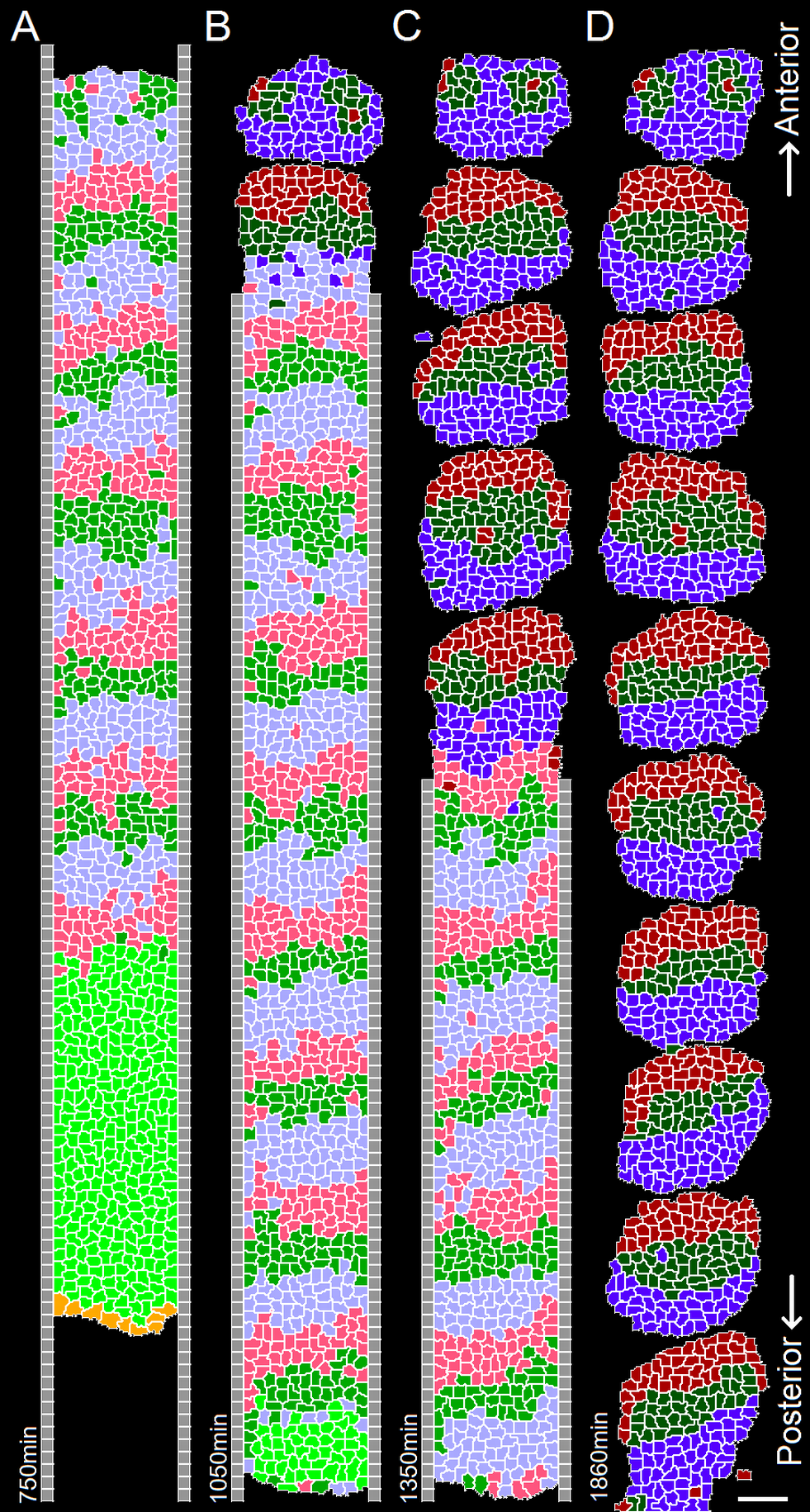

Supplement: Figure S10 — Somite formation with increased post-determination cell mixing. Assigning larger determination-differentiation delay (8 cell cycles) and determined cell types adhesion parameters closer to those of PSM cells than in the reference simulation increases cell mixing among distinct determined cell types prior to differentiation. However, cell sorting after differentiation corrects their moderate amount of mixing across presumptive somite borders and leads to clean somite boundaries. Times: (A) 750 min, (B) 1050 min, (C) 1350 min and (D) 1860 min. Anterior at top. Scale bar 40 µm. Contact energies: Jpre_EphA4,pre_EphA4 = −22; Jpre_ephrinB2,pre_ephrinB2 = −22; Jpre_Core,pre_Core = −25; Jpre_EphA4,EphA4 = −22; Jpre_ephrinB2,ephrinB2 = −22; other contact energies are unchanged from Table 3 . Parameters, when not otherwise noted, are equal to those in the reference simulation ( Figure 7 ). Cell colors are the same as in Figure 5 . Due to non-biological initial conditions, the first somite is always defective. For more information see RESULTS : Somites form in silico for a wide range of delays between cell-fate determination and cell differentiation for determined cells with intermediate adhesion properties. (TIF) [file pcbi.1002155.s010.tif]

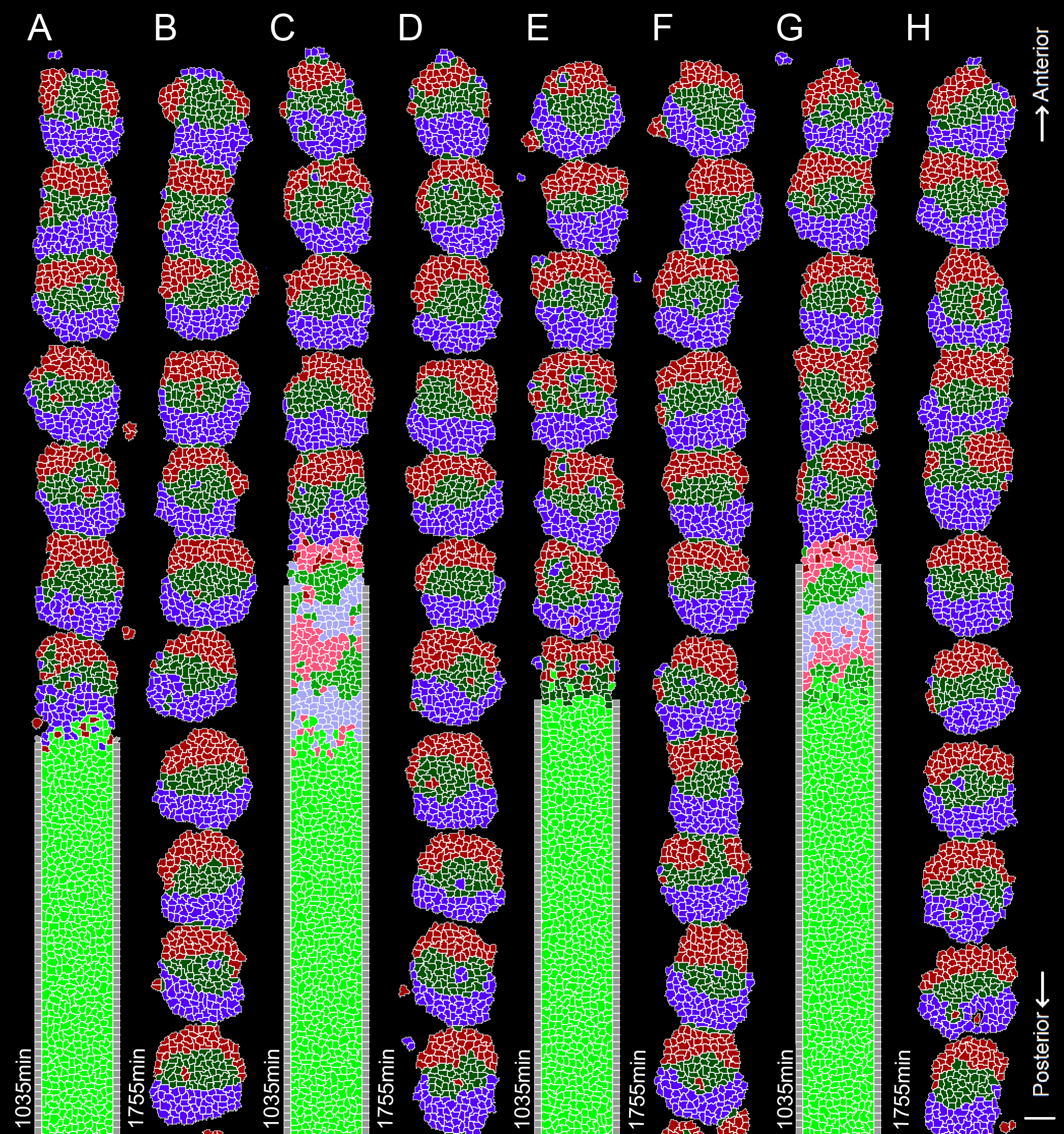

Supplement: Figure S11 — Effect of intermediate adhesion levels between determination and differentiation on segregation quality. For an interval of two or more segmentation-clock periods between cell determination and any changes in adhesion and in the absence of a period of intermediate adhesion, the excessive mixing of determined cell types across their original borders leads to fused somites and a greater-than-normal occurrence of stranded Core cells in the intersomitic gaps: (A–B) two-clock-period interval after (A) 1035 min and (B) 1755 min; (E–F) two-and-a-half-clock-period interval after (E) 1035 min and (F) 1755 min. When cells have determined-cell-type adhesions for the remainder of the standard four-segmentation-clock-period determination-differentiation delay, cell over-mixing is partially corrected, preventing formation of fused somites and decreasing the occurrence of stranded Core cells for an interval of two segmentation-clock periods (C–D), and decreasing the frequency of fused somites and stranded Core cells for longer intervals (G–H): (C–D) two-clock-period interval followed by intermediate adhesion after (C) 1035 min and (D) 1755 min; (G–H) two-and-a-half-clock-period interval followed by intermediate adhesion after (G) 1035 min and (H) 1755 min. Except where otherwise stated, parameters are equal to those in the reference simulation ( Figure 7 ). Cell colors are the same as in Figure 5 . For more information see RESULTS : Somite formation in silico is sensitive to long intervals between determination and adhesion-property changes. (TIF) [file pcbi.1002155.s011.tif]
