# Supplementary material for: A Multi-cell, Multi-scale Model of Vertebrate Segmentation and Somite Formation
Source: PLoS Comput Biol. 2011 Oct 6;7(10):e1002155. doi: 10.1371/journal.pcbi.1002155 (PMC3188485; doi:10.1371/journal.pcbi.1002155)
Supplement: Table S1 — Model segmentation clock network parameters and initial conditions. (PDF) [file pcbi.1002155.s012.pdf]

MODEL SEGMENTATION CLOCK NETWORK PARAMETERS AND INITIAL CONDITIONS

Parameters:

Notch loop

Parameter	Description	Value
v_{sN}	Maximum rate of Notch synthesis	0.23 nM/min
v_{dN}	Maximum rate of Notch degradation	2.82 nM/min
K_{dN}	Michaelis constant for Notch degradation	1.4 nM
k_c	Rate constant for Notch cleavage	3.45 min^{-1}
v_{dNa}	Maximum rate of cytoplasmic NICD degradation	0.01 nM/min
K_{dNa}	Michaelis constant for cytoplasmic NICD degradation	0.001 nM
v_{dNan}	Maximum rate of nuclear NICD degradation	0.1 nM/min
K_{dNan}	Michaelis constant for nuclear NICD degradation	0.001 nM
K_{IF}	Threshold constant for inhibition of Notch cleavage by Lfng	0.45 nM
K_{aDL}	Delta signaling threshold constant for Notch cleavage	0.035 nM
k_{t1}	Rate constant for NICD entry into nucleus	0.1 min^{-1}
k_{t2}	Rate constant for NICD exit from the nucleus	0.1 min^{-1}
**v_{Nap}**	**Maximum fraction of NICD phosphorylated**	**1.0**
**K_{pN}**	**Threshold constant for Gsk3β-mediated NICD phosphorylation**	**2.5 nM**
v_{sF}	Maximum rate of Lfng transcription	3.24 nM/min
K_A	Threshold constant for activation of Lfng gene by NICD	0.05 nM
v_{mF}	Maximum rate of Lfng mRNA degradation	1.92 nM/min
K_{dmF}	Michaelis constant for Lfng mRNA degradation	0.768 nM
k_{sF}	Rate constant for Lfng protein synthesis	0.3 min^{-1}
v_{dF}	Maximum rate of Lfng protein degradation	0.39 nM/min
K_{dF}	Michaelis constant for Lfng protein degradation	0.37 nM
**v_{s0MDMF}**	**Maximum rate of DMF transcription**	**0.497 nM/min**
**K_{aNDMF}**	**Michaelis constant for Notch activation of DMF**	**0.05 nM**
**v_{dmDMF}**	**Maximum rate of DMF mRNA degradation**	**0.314 nM/min**
**K_{dmDMF}**	**Michaelis constant for DMF mRNA degradation**	**0.4 nM**
**K_{sDMF}**	**Rate constant for translation of DMF**	**0.1047 min^{-1}**
**v_{dDMF}**	**Maximum rate of DMF protein degradation**	**0.209 nM/min**
**K_{dDMF}**	**Michaelis constant for DMF protein degradation**	**0.5 nM**
**k_{sDL}**	**Rate constant for Delta protein synthesis**	**0.75 min^{-1}**
**MDL**	**Concentration of Delta mRNA**	**0.5 nM**
**K_{Nan}**	**Michaelis constant for NICD stimulation of Delta translation**	**0.04 nM**
**k_{tDL}**	**Rate constant for Delta protein transport to membrane**	**0.5 min^{-1}**
**v_{dDLc}**	**Maximum rate of Delta protein degradation in cytoplasm**	**0.5 nM/min**
**K_{dDLc}**	**Michaelis constant for Delta protein degradation in cytoplasm**	**0.5 nM**
**v_{dDLm}**	**Maximum rate of Delta protein degradation on membrane**	**0.5 nM/min**
**K_{dDLm}**	**Michaelis constant for Delta protein degradation on membrane**	**0.5 nM**
**K_{DL}**	**Threshold constant for Delta signaling**	**0.08 nM**
ε	Scaling factor for Notch loop	0.43

Parameters in **bold** are additions to the Goldbeter-Pourquié model.

Wnt loop

Parameter	Description	Value
a_1	Rate constant for Gsk3β-Axin2 binding	1.8 $nM^{-1} min^{-1}$
d_1	Rate constant for Gsk3β-Axin2 dissociation	0.1 min^{-1}
v_{sB}	Maximum rate of β-catenin synthesis	0.087 nM/min
k_{t3}	Rate constant for β-catenin entry into the nucleus	0.7 min^{-1}
k_{t4}	Rate constant for β-catenin exit from the nucleus	1.5 min^{-1}
V_{MK}	Maximum rate of Gsk3β-mediated β-catenin phosphorylation	4.5 nM/min
K_t	Total Gsk3β concentration	3.0 nM
K_{ID}	Threshold constant for Dsh inhibition of β-catenin phosphorylation	0.5 nM
K_1	Michaelis constant for β-catenin phosphorylation	0.28 nM
V_{MP}	Maximum rate of β-catenin dephosphorylation	1.0 nM/min
K_2	Michaelis constant for β-catenin dephosphorylation	0.03 nM
k_{d1}	Rate constant for unphosphorylated β-catenin degradation	0.0 min^{-1}
k_{d2}	Rate constant for phosphorylated β-catenin degradation	7.062 min^{-1}
v_0	Basal rate of Axin2 transcription	0.06 nM/min
v_{MB}	Maximum rate of β-catenin activated Axin2 transcription	1.64 nM/min
K_{aB}	Threshold constant for β-catenin activation of Axin2	0.7 nM
v_{md}	Maximum rate of Axin2 mRNA degradation	0.8 nM/min
K_{md}	Michaelis constant for Axin2 mRNA degradation	0.48 nM
v_{MXa}	Maximum rate of Xa activated Axin2 transcription	0.5 nM/min
K_{aXa}	Threshold constant for Xa activation of Axin2	0.05 nM
K_{sAx}	Rate constant for Axin2 translation	0.02 min^{-1}
v_{dAx}	Maximum rate of Axin2 protein degradation	0.6 nM/min
K_{dAx}	Michaelis constant for Axin2 protein degradation	0.63 nM
θ	Scaling factor for Wnt loop	1.12

FGF loop

Parameter	Description	Value
Ras_t	Total concentration of Ras protein	2.0 nM
V_{MaRas}	Maximum rate of Ras activation	4.968 nM/min
K_{aFgf}	FGF8 threshold constant for activation of Ras	0.5 nM
K_{aRas}	Inactive Ras threshold constant for activation of Ras	0.103 nM
V_{MdRas}	Maximum rate of Ras activation	0.41 nM/min
K_{dRas}	Michaelis constant for Ras inactivation	0.1 nM
ERK_t	Total concentration of ERK protein kinase	2.0 nM
V_{MaErk}	Maximum rate of Ras-mediated ERK activation	3.3 nM/min
K_{aErk}	Inactive ERK threshold constant for ERK activation	0.05 nM
K_{cDusp}	Rate constant for inactivation of ERK	1.35 min^{-1}
K_{dErk}	Michaelis constant for inactivation of ERK	0.05 nM
X_t	Total concentration of factor X	2.0 nM
V_{MaX}	Maximum rate of ERK-mediated X activation	1.6 nM/min
K_{aX}	Threshold constant for ERK-mediated X activation	0.05 nM
V_{MdX}	Maximum rate of X inactivation	0.5 nM/min
K_{dX}	Michaelis constant for X inactivation	0.05 nM
$V_{MsMDusp}$	Maximum rate of Dusp6 transcription	0.9 nM/min
K_{aMDusp}	Threshold constant for X-activated Dusp6 transcription	0.5 nM
$V_{MdMDusp}$	Maximum rate of Dusp6 mRNA degradation	0.5 nM/min
K_{dMDusp}	Michaelis constant for Dusp6 mRNA degradation	0.5 nM
k_{sDusp}	Rate constant for Dusp6 translation	0.5 min^{-1}

V_{dDusp}	Maximum rate of Dusp6 protein degradation	2.0 nM/min
K_{dDusp}	Michaelis constant for Dusp6 protein degradation	0.5 nM
$\mathbf{v_{DuspX}}$	**Fraction of Dusp6 transcription solely regulated by X**	**0.2**
$\mathbf{v_{DuspDMF}}$	**Fraction of Dusp6 transcription under regulation by DMF**	**0.8**
$\mathbf{K_{IMDusp}}$	**Threshold constant for DMF-mediated inhibition of Dusp6**	**0.3 nM**
v	Scaling factor for FGF8 loop	0.328

Parameters in **bold** are additions to the Goldbeter-Pourquié model.

Initial variable values:

Notch loop

Variable	Description	Value
N	Notch in the membrane	0.0552 nM
N_a	Activated Notch	0.8095 nM
N_{an}	Activated Notch in the nucleus	0.0039 nM
$\mathbf{N_{ap}}$	**Phosphorylated Notch**	0.0013 **nM**
MF	Lunatic Fringe mRNA	0.003 nM
F	Lunatic Fringe protein	0.0006 nM
MDMF	**Delta Modification Factor mRNA**	0.0011 **nM**
DMF	**Delta Modification Factor protein**	0.0003 **nM**
$\mathbf{DL_c}$	**Delta protein in the cytoplasm**	0.0138 **nM**
$\mathbf{DL_m}$	**Delta protein on the membrane**	0.0058 **nM**

Variables in **bold** are additions to the Goldbeter-Pourquié model.

Wnt loop

Variable	Description	Value
K	Free Gsk3 protein	1.2252 nM
B	β-catenin	0.9118 nM
B_p	Phosphorylated β-catenin	0.0171 nM
B_N	Nuclear β-catenin	0.4324 nM
Max	Axin2 mRNA	7.7560 nM
A	Axin2 protein	0.1076 nM
AK	Axin2-Gsk3 complex	1.7747 nM

FGF loop

Variable	Description	Value
Ras_a	Activated Ras protein	1.9912 nM
ERK_a	Activated ERK protein	1.9780 nM
X_a	Activated transcription factor X	1.9777 nM
MDusp	Dusp mRNA	2.9054 nM
Dusp	Dusp protein	0.7692 nM
