# Supplementary material for: A Multi-cell, Multi-scale Model of Vertebrate Segmentation and Somite Formation
Source: PLoS Comput Biol. 2011 Oct 6;7(10):e1002155. doi: 10.1371/journal.pcbi.1002155 (PMC3188485; doi:10.1371/journal.pcbi.1002155)
Supplement: Text S1 — Model segmentation clock network equations. (PDF) [file pcbi.1002155.s013.pdf]

MODEL SEGMENTATION CLOCK NETWORK EQUATIONS

The ordinary differential equations used to model the segmentation clock are modified from those used by Goldbeter and Pourquié [12] with a few additions, mainly on the Delta-Notch pathway, to account for cell-cell synchronization [5] and increased connection between the internal pathways.

Notch loop:

$$\frac{dN}{dt} = \varepsilon\left(v_{\mathrm{sN}} - v_{\mathrm{dN}} \cdot \frac{N}{K_{\mathrm{dN}} + N} - N_{\mathrm{Sig}} \right) \tag{S1}$$

$$\frac{dN_{\mathrm{a}}}{dt} = \varepsilon\left(N_{\mathrm{Sig}} - v_{\mathrm{dNa}} \cdot \frac{N_{\mathrm{a}}}{K_{\mathrm{dNa}} + N_{\mathrm{a}}} - V_{\mathrm{tr}} \right) \tag{S2}$$

$$\frac{dN_{\mathrm{an}}}{dt} = \varepsilon\left(V_{\mathrm{tr}} - v_{\mathrm{dNan}} \cdot \frac{N_{\mathrm{an}}}{K_{\mathrm{dNan}} + N_{\mathrm{an}}} \right) \tag{S3}$$

$$\frac{dMF}{dt} = \varepsilon\left(v_{\mathrm{sF}} \cdot \frac{(N_{\mathrm{an}} - N_{\mathrm{ap}})^2}{K\mathrm{A}^2 + (N_{\mathrm{an}} - N_{\mathrm{ap}})^2} - v_{\mathrm{mF}} \cdot \frac{MF}{K_{dmF} + MF} \right) \tag{S4}$$

$$\frac{dF}{dt} = \varepsilon\left(k_{\mathrm{sF}} \cdot MF - v_{\mathrm{dF}} \cdot \frac{F}{K_{\mathrm{dF}} + F} \right) \tag{S5}$$

$$\frac{dMDMF}{dt} = \varepsilon\left(v_{\mathrm{s0MDMF}} \cdot \frac{(N_{\mathrm{an}} - N_{\mathrm{ap}})^2}{K_{\mathrm{aNDMF}}^2 + (N_{\mathrm{an}} - N_{\mathrm{ap}})^2} - v_{\mathrm{dmDMF}} \cdot \frac{MDMF}{K_{\mathrm{dmDMF}} + MDMF} \right) \tag{S6}$$

$$\frac{dDMF}{dt} = \varepsilon\left(k_{\mathrm{sDMF}} \cdot MDMF - v_{\mathrm{dDMF}} \cdot \frac{DMF}{K_{\mathrm{dDMF}} + DMF} \right) \tag{S7}$$

$$\frac{dDL_{\mathrm{c}}}{dt} = \varepsilon\left(k_{\mathrm{sDL}} \cdot MDL \cdot \frac{(N_{\mathrm{an}} - N_{\mathrm{ap}})}{K_{\mathrm{Nan}} + (N_{\mathrm{an}} - N_{\mathrm{ap}})} - k_{\mathrm{tDL}} \cdot DL_{\mathrm{c}} - v_{\mathrm{dDLc}} \cdot \frac{DL_{\mathrm{c}}}{K_{\mathrm{dDLc}} + DL_{\mathrm{c}}} \right) \tag{S8}$$

$$\frac{dDL_{\mathrm{m}}}{dt} = \varepsilon\left(k_{\mathrm{tDL}} \cdot DL_{\mathrm{c}} - v_{\mathrm{dDLm}} \cdot \frac{DL_{\mathrm{m}}}{K_{\mathrm{dDLm}} + DL_{\mathrm{m}}} \right) \tag{S9}$$

$$N_{\mathrm{Sig}} = k_{\mathrm{c}} \cdot N \cdot \frac{K_{\mathrm{IF}}^2}{K_{\mathrm{IF}}^2 + F^2} \cdot \frac{DL_{\mathrm{mSig}}}{K_{\mathrm{aDL}} + DL_{\mathrm{mSig}}} \tag{S10}$$

$$DL_{\mathrm{mSig}} = \frac{DL_{\mathrm{mExt}}}{K_{\mathrm{DL}} + DL_{\mathrm{mExt}}} \tag{S11}$$

$$V_{\mathrm{tr}} = k_{\mathrm{t1}} \cdot N_{\mathrm{a}} - k_{\mathrm{t2}} \cdot N_{\mathrm{an}} \tag{S12}$$

$$N_{\mathrm{ap}} = v_{\mathrm{Nap}} \cdot N_{\mathrm{an}} \cdot \frac{K}{K_{\mathrm{pN}} + K} \tag{S13}$$

Wnt loop:

$$\frac{dK}{dt} = \theta \cdot V_1 \tag{S14}$$

$$\frac{dB}{dt} = \theta\left(v_{\mathrm{sB}} - V_{\mathrm{K}} \cdot \frac{AK}{K_{\mathrm{t}}} + V_{\mathrm{P}} + V_2 - k_{\mathrm{d1}} \cdot B\right) \quad \text{(S15)}$$

$$\frac{dB_{\mathrm{p}}}{dt} = \theta\left(V_{\mathrm{K}} \cdot \frac{AK}{K_{\mathrm{t}}} - V_{\mathrm{P}} - k_{\mathrm{d2}} \cdot B_{\mathrm{p}}\right) \quad \text{(S16)}$$

$$\frac{dB_{\mathrm{N}}}{dt} = -\theta \cdot V_2 \quad \text{(S17)}$$

$$\frac{dMAx}{dt} = \theta\left(v_0 + v_{\mathrm{MB}} \cdot \frac{B_{\mathrm{N}}^2}{K_{\mathrm{aB}}^2 + B_{\mathrm{N}}^2} + v_{\mathrm{MXa}} \cdot \frac{X_{\mathrm{a}}^2}{K_{\mathrm{aXa}}^2 + X_{\mathrm{a}}^2} - v_{\mathrm{md}} \cdot \frac{MAx}{K_{\mathrm{md}} + MAx}\right) \quad \text{(S18)}$$

$$\frac{dA}{dt} = \theta\left(k_{\mathrm{sAx}} \cdot MAx - v_{\mathrm{dAx}} \cdot \frac{A}{K_{\mathrm{dAx}} + A} + V_1\right) \quad \text{(S19)}$$

$$V_1 = d_1 \cdot AK - a_1 \cdot A \cdot K \quad \text{(S20)}$$

$$AK = K_{\mathrm{t}} - K \quad \text{(S21)}$$

$$V_{\mathrm{K}} = V_{\mathrm{MK}} \cdot \frac{K_{\mathrm{ID}}}{K_{\mathrm{ID}} + D} \cdot \frac{B}{K_1 + B} \quad \text{(S22)}$$

$$V_{\mathrm{P}} = V_{\mathrm{MP}} \cdot \frac{B_{\mathrm{p}}}{K_2 + B_{\mathrm{p}}} \quad \text{(S23)}$$

$$V_2 = k_{\mathrm{t4}} \cdot B_{\mathrm{N}} - k_{\mathrm{t3}} \cdot B \quad \text{(S24)}$$

FGF loop:

$$\frac{dRas_{\mathrm{a}}}{dt} = \eta\left(V_{\mathrm{MaRas}} \cdot \frac{Fgf^2}{K_{\mathrm{aFgf}}^2 + Fgf^2} \cdot \frac{Ras_{\mathrm{i}}}{K_{\mathrm{aRas}} + Ras_{\mathrm{i}}} - V_{\mathrm{MdRas}} \cdot \frac{Ras_{\mathrm{a}}}{K_{\mathrm{dRas}} + Ras_{\mathrm{a}}}\right) \quad \text{(S25)}$$

$$\frac{dERK_{\mathrm{a}}}{dt} = \eta\left(V_{\mathrm{MaErk}} \cdot \frac{Ras_{\mathrm{a}}}{Ras_{\mathrm{t}}} \cdot \frac{ERK_{\mathrm{i}}}{K_{\mathrm{aErk}} + ERK_{\mathrm{i}}} - k_{\mathrm{cDusp}} \cdot Dusp \cdot \frac{ERK_{\mathrm{a}}}{K_{\mathrm{dErk}} + ERK_{\mathrm{a}}}\right) \quad \text{(S26)}$$

$$\frac{dX_{\mathrm{a}}}{dt} = \eta\left(V_{\mathrm{MaX}} \cdot \frac{ERK_{\mathrm{a}}}{ERK_{\mathrm{t}}} \cdot \frac{X_{\mathrm{i}}}{K_{\mathrm{aX}} + X_{\mathrm{i}}} - V_{\mathrm{MdX}} \cdot \frac{X_{\mathrm{a}}}{K_{\mathrm{dX}} + X_{\mathrm{a}}}\right) \quad \text{(S27)}$$

$$\frac{dMDusp}{dt} = \eta\left(V_{\mathrm{MsMDusp}} \cdot \frac{X_a^2}{K_{\mathrm{aMDusp}}^2 + X_a^2}\left(v_{\mathrm{DuspDMF}} \cdot \frac{K_{\mathrm{IMDusp}}}{K_{\mathrm{IMDusp}} + DMF} + v_{\mathrm{DuspX}}\right) - V_{\mathrm{MdMDusp}} \cdot \frac{MDusp}{K_{\mathrm{dMDusp}} + MDusp}\right) \quad \text{(S28)}$$

$$\frac{dDusp}{dt} = \eta\left(k_{\mathrm{sDusp}} \cdot MDusp - V_{\mathrm{dDusp}} \cdot \frac{Dusp}{K_{\mathrm{dDusp}} + Dusp}\right) \quad \text{(S29)}$$

$$Ras_{\mathrm{i}} = Ras_{\mathrm{t}} - Ras_{\mathrm{a}} \quad \text{(S30)}$$

$$ERK_{\mathrm{i}} = ERK_{\mathrm{t}} - ERK_{\mathrm{a}} \quad \text{(S31)}$$

$$X_{\mathrm{i}} = X_{\mathrm{t}} - X_{\mathrm{a}} \quad \text{(S32)}$$
