# Supplementary material for: A Multi-cell, Multi-scale Model of Vertebrate Segmentation and Somite Formation
Source: PLoS Comput Biol. 2011 Oct 6;7(10):e1002155. doi: 10.1371/journal.pcbi.1002155 (PMC3188485; doi:10.1371/journal.pcbi.1002155)
Supplement: Text S2 — Instructions for downloading and running simulation source code. (PDF) [file pcbi.1002155.s014.pdf]

Source Code Instructions:

To run the reference simulation presented in this paper the reader needs to download the source code and a couple of pieces of software. The reader will need to download and install Python (http://www.python.org) and CompuCell3D (http://www.compucell3d.org) on their computer. Additionally, if the reader is using a Linux or Mac OS X machine they must download and install CMake. All programs are open source, and available for free.

The simulation source code is available in a zip file (*BelmonteHesterSomite.zip*) from http://www.compucell3d.org/BelmonteHesterSomite.

It consists of several files; the following are the most relevant:

1) *Somites.py* (The main CC3D/Python file with the model parameters)
2) *Somites_Step.py* (The CC3D/Python file were all calculations are implemented)
3) *SegClock.cpp* (C++ code implementing the segmentation-clock)
4) *SegClock.h* (C++ header file)
5) *somite_demo_setup.exe* (Windows setup program)
6) *CMakeLists.txt* (Setup file for Linux/OS X users)

This .zip file should be decompressed in a convenient location for the reader.

To prepare to run the simulation on CompuCell3D:

For **Windows** users:

1) In the folder where the .zip file was decompressed, double-click on: *somite_demo_setup.exe*
2) When prompted for an installation destination, select the folder CompuCell3D was installed in (Typically: C:\Program Files (x86)\CompuCell3D\)

For **Linux** or **Mac** users:

1) Start the CMake GUI, and click on Browse Source
2) Browse to the folder where the .zip file was decompressed.
3) Click on Browse Build and select the same folder as step 2
4) Click on Configure, when prompted select: "Unix Makefiles," and "Use default native compilers," and click Done.
5) Click on "COMPUCELL3D_PATH" and enter the location where CompuCell3D was installed, and click Configure.
6) Click on Generate, and then exit CMake
7) From a Terminal window, browse to the folder where the .zip file was decompressed.
8) Type "make" and press return. When it has completed type "make install" and press return.

To run the simulation:

1) Start CompuCell3D.
2) From the file menu select: Open Simulation File.
3) Browse to the CompuCell3D Demos folder.
4) Inside the folder BelmonteHesterSomite, select *Somites.py*, and click Open
5) Click the Play button in the upper left hand side of the CompuCell3D window

The simulation with the **cells**' initial condition should appear and the **PSM** should start to grow. To watch the FGF8/Wnt3a gradients, and the proteins concentrations the reader must go to the Main Graphics Window and, on the upper right, click where it says "Cell_Field" and select the desired visualization.

The files are configured with the reference simulation parameters. As an example of how to modify the simulation, the reader needs to open the main python file (*Somites2010.py*) and on line 37 change the **PSM cells**' growth rate from 0.120 to 0.240. Save the file and load it again on CompuCell3D. Because the **PSM** grows twice faster than before, there will be a higher amount of cell material coming in and the simulation will yield bigger somites similar to the ones shown in **Figure 13** (**C**). The ML size of the **PSM** can be adjusted on line 23 (change it from 10*cd to 20*cd to get more circular **somites**).
