# Supplementary material for: A Multi-cell, Multi-scale Model of Vertebrate Segmentation and Somite Formation
Source: PLoS Comput Biol. 2011 Oct 6;7(10):e1002155. doi: 10.1371/journal.pcbi.1002155 (PMC3188485; doi:10.1371/journal.pcbi.1002155)
Supplement: Text S3 — Assumptions and features of the integrated model. (PDF) [file pcbi.1002155.s015.pdf]

Major assumptions and simplifications of the integrated model

(1) A 2D AP-by-ML slice along the DV midline can serve as a proxy for 3D somitogenesis;
(2) PSM growth in the AP direction is primarily due to the posterior addition of cells from the tail bud, rather than proliferation of cells within and throughout the PSM;
(3) Cell growth and proliferation can be neglected in the simulated PSM region;
(4) Cells enter the middle of the PSM with uniform intracellular concentrations of FGF8, Wnt3a and *fgf8* mRNA;
(5) Cells enter the middle of the PSM with their segmentation clocks synchronized to their immediate medio-lateral neighbors;
(6) Differences in random motility among PSM cells can be neglected in the simulated region;
(7) Gradual changes in adhesion properties can be approximated by two abrupt changes occurring at determination and differentiation, respectively;
(8) Segmentation and somite formation dynamics can be separated from epithelialization and non-adhesion-related Eph-ephrin signaling;
(9) Details of ECM-PSM interaction can be neglected at the level of detail of the model;
(10) The effect on the morphogen gradients of endocytosis-assisted passage of Wnt3a and FGF8 through cells can be neglected at the level of detail of the model;
(11) At the level of detail of the model, the most important role of the external environment is to physically constrain the PSM in medial, lateral and dorsal and ventral directions;
(12) Each cell has an identical internal three-oscillator segmentation-clock network, the behavior of which can be approximated by a deterministic ODE reaction-kinetics submodel in the simulated region;
(13) The phases of Paraxis and cMeso1 expression closely resemble those of Axin2 and Lfng at the time of determination;
(14) A sub-threshold concentration of FGF8 induces cell determination;
(15) The adhesion of determined and differentiated cells depends only on four transmembrane proteins (N-CAM, N-cadherin, EphA4 and ephrinB2) according to **Table 3**.

Experimental features included and reproduced by the model

Included:

(1) Cells occupy volume and have deformable shapes;

(2) Cells have intrinsic random motilities distinct from directed cell motion due to, *e.g.*, sorting at the forming somite borders;

(3) Realistic extracellular FGF8 concentration gradient;

Reproduced:

(4) An extended stripe of PSM tissue of constant length;

(5) Realistic Wnt3a governed variation in segmentation-clock period;

(6) Medio-lateral stripes of high Lfng concentration that travel from the posterior to the anterior of the PSM;

(7) Lfng stripes narrow in the anterior-posterior direction as they move anteriorly;

(8) Segmentation clocks between neighbor cells phase lock;

(9) An unlimited number of regular somites of controlled size;

(10) Misplaced cell types in the forming somite occur at a rate consistent with experiments;

(11) Spontaneous intrasomitic border correction of misplaced cell types after differentiation;

(12) Somite-to-somite shape variation is consistent with experiments;

(13) Parameter variation leads to interspecies-like variability in somites:

a. Somite size depends on the segmentation-clock period;
b. Somite size depends on the rate of the PSM growth;
c. The number of Lfng stripes depends on the segmentation-clock period;
d. The number of Lfng stripes depends on the ratio of the PSM length to the PSM growth rate;
e. Lfng stripe width depends on the rate of Wnt3a decay (*i.e.,* on the slope of the Wnt3a gradient);
